# Multiple *Sox *genes are expressed in stem cells or in differentiating neuro-sensory cells in the hydrozoan *Clytia hemisphaerica*

**DOI:** 10.1186/2041-9139-2-12

**Published:** 2011-06-01

**Authors:** Muriel Jager, Eric Quéinnec, Hervé Le Guyader, Michaël Manuel

**Affiliations:** 1 UPMC Univ Paris 06, UMR 7138 CNRS UPMC MNHN IRD, Case 05, 7 quai St Bernard, 75005 Paris, France

## Abstract

**Background:**

The *Sox *genes are important regulators of animal development belonging to the HMG domain-containing class of transcription factors. Studies in bilaterian models have notably highlighted their pivotal role in controlling progression along cell lineages, various *Sox *family members being involved at one side or the other of the critical balance between self-renewing stem cells/proliferating progenitors, and cells undergoing differentiation.

**Results:**

We have investigated the expression of 10 *Sox *genes in the cnidarian *Clytia hemisphaerica*. Our phylogenetic analyses allocated most of these *Clytia *genes to previously-identified Sox groups: SoxB (*CheSox2, CheSox3, CheSox10, CheSox13, CheSox14*), SoxC (*CheSox12*), SoxE (*CheSox1, CheSox5*) and SoxF (*CheSox11*), one gene (*CheSox15*) remaining unclassified. In the planula larva and in the medusa, the *SoxF *orthologue was expressed throughout the endoderm. The other genes were expressed either in stem cells/undifferentiated progenitors, or in differentiating (-ed) cells with a neuro-sensory identity (nematocytes or neurons). In addition, most of them were expressed in the female germline, with their maternal transcripts either localised to the animal region of the egg, or homogeneously distributed.

**Conclusions:**

Comparison with other cnidarians, ctenophores and bilaterians suggest ancient evolutionary conservation of some aspects of gene expression/function at the *Sox *family level: (i) many *Sox *genes are expressed in stem cells and/or undifferentiated progenitors; (ii) other genes, or the same under different contexts, are associated with neuro-sensory cell differentiation; (iii) *Sox *genes are commonly expressed in the germline; (iv) *SoxF *group genes are associated with endodermal derivatives. Strikingly, total lack of correlation between a given *Sox *orthology group and expression/function in stem cells/progenitors *vs*. in differentiating cells implies that *Sox *genes can easily switch from one side to the other of the balance between these fundamental cellular states in the course of evolution.

## Background

The *Sox *genes, a metazoan-specific family of HMG-domain containing transcription factors, are important regulators of animal development. In mammals and in classical non-vertebrate models (*Drosophila melanogaster, Caenorhabditis elegans*), studies of *Sox *gene expression and function have highlighted their crucial involvement in a great diversity of developmental contexts, for example, in neurogenesis, cardiogenesis, angiogenesis, chondrogenesis, and endoderm development [1-3]. *Sox *genes are also involved in adult tissue homeostasis and in disease, notably cancer [2-4]. At the molecular level, *Sox *genes activate, repress or modulate transcription of target genes through physical interaction with a variety of partner proteins. The mechanisms whereby this transcriptional regulation is mediated are remarkably diverse [5,6].

There is significant diversity within the *Sox *multigenic family, with, for example, 20 paralogues in the mammalian genome and 8 in the fly genome [7]. Previous gene phylogenies have identified five major *Sox *groups (named B, C, D, E and F) [8-12]. With the exception of *SoxD*, all of them are represented in the genomes of bilaterians as well as non-bilaterian eumetazoans (cnidarians and ctenophores) [8-10]. Several more artificial "groups" (for example, group A, G, H, I, J) have been created to accommodate single genes that are difficult to position in the *Sox *tree. The presence of putative members of families B, C and F in sponges furthermore suggests that *Sox *genes started to duplicate before the last common ancestor of Metazoa [9,11,12].

A recurrent theme in functional studies of *Sox *functions at the cellular level in bilaterian models is the involvement of various members of the family in the critical balance between self-renewing stem cells/proliferating progenitors, and cells undergoing differentiation, and their pivotal role in the regulation of this equilibrium in numerous developmental contexts [2,3,13,14]. For example, the vertebrate *Sox2 *gene is widely known as a key factor for maintenance of mammalian ES cell pluripotency [13-17]. Its forced expression (together with *Oct4, Klf4 *and *c-Myc*) in differentiated fibroblasts leads to their re-programming into ES-like pluripotent cells [18]. In central nervous system development, the same *Sox2 *gene acts in synergy with other *SoxB *group genes (*Sox1 *and *Sox3*) to maintain neural stem cells and to repress neuronal differentiation, whereas yet other *SoxB *genes (*Sox21 *and *Sox14*) promote cell cycle exit and neuronal differentiation under the control of the proneural genes [14,17]. The same *Sox *protein can sometimes act on one side or the other of the balance between proliferating and differentiating cells depending on the developmental context, as is the case of the vertebrate *Sox2 *gene, involved in the terminal differentiation of some neuronal subtypes [13,14,17], in addition to its earlier function in neural stem cell maintenance.

Data from non-vertebrate bilaterians such as insects, *Caenorhabditis elegans *(nematode), sea limpet (mollusc), *Platynereis *(annelid) and sea urchin (echinoderm) suggest evolutionary conservation of at least some aspects of *Sox *gene functions, notably in neurogenesis and in gametogenesis [19,20]. In the annelid *Platynereis dumerilii*, a *SoxB *group gene was found to be expressed in the neurectoderm before the formation of committed neural precursors, while the expression of a *SoxC *group gene evoked a role in neuronal differentiation [19]. These data are consistent with involvement of these two genes at different sides of the balance between cell proliferation and differentiation along the neuronal cell lineage. However, for most invertebrate *Sox *genes (including *Drosophila *genes), expression and function have not been precisely characterised in terms of stages and progression along cell lineages. Therefore, it remains unclear whether *Sox *family genes have evolutionarily conserved roles in these processes, and if it is the case, whether each particular *Sox *orthology group was ancestrally associated with one particular side of the balance, that is, either with stem cells/progenitors, or with differentiating cells.

Studies in animal lineages that branch outside bilaterians are expected to be informative about the early stages of animal evolution. Phylogenetic relationships between the early-diverging animal phyla remain contentious [21-24], but a critical re-analysis of data sets used in recent phylogenomic studies suggests that apparent conflicts between them disappear when errors are corrected and appropriate taxon sampling and models are used [25]. Currently the best-supported phylogenomic estimate of basal metazoan relationships implies the monophyly of animals with nerve cells and muscle cells (Eumetazoa: cnidarians, ctenophores and bilaterians) in line with classical views, and the grouping of cnidarians and ctenophores in a coelenterate clade sister-group to the Bilateria [22]. Previously-published data on *Sox *gene expression in two anthozoan cnidarians (the sea anemone *Nematostella vectensis *[8], and the coral *Acropora millepora *[26] and in a ctenophore [10] have started to unveil conserved features of *Sox *gene expression at the eumetazoan level. In particular, these three studies all concluded that involvement of some *Sox *genes in neuro-sensory cell specification and differentiation probably dates back to the common eumetazoan ancestor. In addition, two of the ctenophore *Sox *genes were found expressed in the germ line as well as in several somatic territories recently characterised as reservoirs of somatic stem cells [27]. It was therefore proposed that *Sox *roles in the balance between stem cells/progenitors and differentiating cells might be conserved at the eumetazoan scale [10]. However, there is currently no data from cnidarians to fuel this hypothesis, notably because stem cells and progenitors have not been characterised in the larvae and adults of the two anthozoans in which *Sox *gene expression has been investigated [8,26].

To gain insight into evolutionary conservation and divergence of *Sox *gene expression characteristics in relation to progression along cell lineages, we investigated the expression of *Sox *genes in the hydrozoan cnidarian *Clytia hemisphaerica *[28]. Hydrozoan cnidarians have multipotent stem cells, called interstitial stem cells, whose progeny comprises neuro-sensory cells (including the stinging cells or nematocytes), gland cells, and germ cells [29-34]. These interstitial cells appear in the endoderm after gastrulation [29]. The planula larva has endodermal patches of interstitial stem cells already providing larval nematoblasts, nerve cells and gland cells [29]. Upon metamorphosis, interstitial cells migrate to the ectoderm, where they remain localised in the adults [29,34]. The *C. hemisphaerica *life cycle comprises two alternating adult forms: the asexual benthic colony of polyps, and the sexual pelagic medusa [28]. In a previous work [35], it was shown that the medusa contains localised populations of somatic stem cells, notably two symmetrical patches of stem cells positioned in the proximal region of each tentacle bulb. Tentacle bulbs are specialised basal swellings of the tentacles, in which tentacle nematocytes are generated all life long. There is a gradient of nematogenesis stages from the proximal to the distal pole of the tentacle bulb axis [35]. Thanks to these features, genes expressed during nematogenesis in the medusa can be easily characterised either as stem cell/progenitor genes or as early or late differentiation genes, based on the spatial position of their expression zone along the tentacle bulb axis.

Here, we present detailed expression analyses of 10 *Sox *genes (five members of group B, one group C gene, 2 members of group E, one group F gene and one unclassified *Sox *gene) in the *Clytia hemisphaerica *planula larvae, medusae and eggs. The results suggest conservation at a deep evolutionary level of the general features of *Sox *gene expression: the *SoxF *orthologue has endodermal expression in *Clytia *like in other non-bilaterian animals investigated so far, whereas for all other orthology groups, the genes are expressed either in somatic stem cells and in the germ line, or in differentiating/differentiated cells with a neuro-sensory identity (either nematocytes or nerve cells). However, comparison with gene expression data from ctenophore and bilaterians reveals total lack of correlation between any particular *Sox *group and expression/function in stem cells/progenitors *vs*. in differentiating cells, thus indicating that the roles of individual *Sox *genes can easily switch from one side to the other of the balance, in different developmental and evolutionary contexts.

## Results

### Phylogenetic relationships of *Clytia Sox *genes

**Figure 1:**
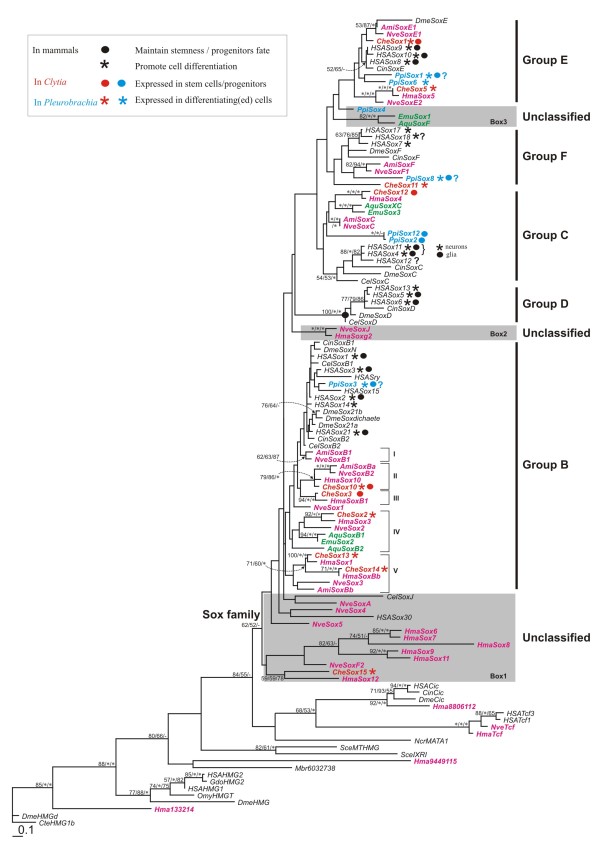
**Phylogenetic analyses of *Sox *HMG domains**. The tree was computed using Maximum Likelihood (ML) from an amino-acid alignment of complete HMG domain sequences (79 amino-acids; except for *CheSox1, PpiSox2, PpiSox3, PpiSox12, EmuSox1, EmuSox2 *and *EmuSox3*, for which only the 68 C-terminal amino-acids were included). The tree likelihood was logL = -8933.874657. Numbers associated with the branches correspond to ML bootstrap proportions (100 replicates)/NJ bootstrap values (1.000 replicates)/Bayesian posterior probabilities. Support values below 50% are indicated by a dash; maximal support values by a star. Abbreviated species names as follows: *Acropora millepora, Ami; Amphimedon queenslandica, Aqu; Caenorhabditis elegans, Cel; Ciona intestinalis, Cin; Clytia hemisphaerica, Che; Drosophila melanogaster, Dme; Ephydatia muelleri, Emu; Gallus domesticus, Gdo; Globodera rostochiensis, Gro; Hydra magnipapillata, Hma; Homo sapiens, HSA; Monosiga brevicollis, Mbr; Nematostella vectensis, Nve; Oncorhynchus mykis, Omy; Pleurobrachia pileus, Ppi; Neurospora crassa, Ncr; Saccharomyces cerevisiae, Sce*. Genes from *Clytia hemisphaerica *investigated in the present expression study are in red. Other *Sox *cnidarian sequences are indicated in pink; sponge sequences are in green, ctenophore sequences in blue and bilaterian sequences in black. The main *Sox *groups are indicated on the right. Unclassified sequences are highlighted using grey boxes. Symbols were used to label genes associated with either undifferentiated state and proliferation (circle) or cell differentiation (star) in three animal taxa: mammals [2,3,48,57], the ctenophore *P. pileus *[10] and *C. hemisphaerica *(this study).

Until now, 15 members of the *Sox *family have been identified in *Clytia hemisphaerica*, of which 10 have complete or almost complete HMG domain sequences and were included in the phylogenetic analyses (Figure 1). Expression data are reported here for these 10 genes (*CheSox1, 2, 3, 5, 10, 11, 12, 13, 14*, and *15*). Among them, five are new with respect to a previously published survey of *Clytia Sox *genes [9].

Maximum-Likelihood (ML) and distance Neighbour-Joining (NJ) analyses recovered the monophyly of the metazoan-specific *Sox *family and of most previously identified groups (Group B, Group C, Group E, Group F and the bilaterian-specific group D) (Figure 1 and [10]). However, some sequences cannot be classified into any of these groups (grey boxes in Figure 1), including one ctenophore sequence (*PpiSox4*), two sponge sequences (*EmuSox1 *and *AquSoxF*) and several cnidarian *Sox *genes from various species (*Hydra magnipapillata, Nematostella vectensis *and *Clytia hemisphaerica*). In particular, genes highlighted in Box 1 (Figure 1) are problematic because they fall in a basal and divergent position in the *Sox *family. They comprise mostly cnidarian genes, but also the nematode *CelSoxJ *gene and the human *HsaSox30 *gene.

The remaining *Clytia Sox *genes are allocated to groups B, C, E, F in accordance with previous gene assignments [9]. As in previous studies based on extensive gene repertoires [8-11], these groups are not supported by statistical indices (support was obtained only in studies using partial gene samplings, *for example*, [12,26]). Like in our previous works on *Clytia *and *Pleurobrachia *Sox genes [9,10], genes are named in a neutral way using numbers that are without any implication in terms of gene orthology within the Sox family. According to the tree topology, *CheSox1 *and *CheSox5 *belong to group E, *CheSox2, 3, 10, 13 *and *14 *to group B, while *CheSox11 *branches within group F and *CheSox1*2 within group C. Most *Clytia *genes have clear orthologues from *Hydra magnipapillata*, supported by ML BS values between 50% and 100%, except for the two genes *CheSox1 *and *CheSox11 *(without identified *Hydra *orthologues). Most *Clytia *genes also have recognisable anthozoan orthologues (Figure 1).

Within group B, all paralogues from bilaterian species group in a clade also containing the ctenophore *PpiSox3 *gene, while the highly diversified cnidarian genes fall in a basal paraphyletic assemblage. Within this assemblage, we have labelled five distinct genes sets for clarity (I to V on Figure 1). Three of these gene sets (II, IV and V) contain genes from both anthozoans and hydrozoans, thereby implying that the ancestral cnidarian genome comprised at least three *SoxB *paralogues. The number of ancestral cnidarian *SoxB *genes might even have been higher, since we cannot exclude the possibility of undetectable orthologies among the remaining hydrozoan (gene set III) and anthozoan (gene set I and *NveSox1*) sequences. There is one instance of a clear hydrozoan-specific duplication, within gene set V, with *Clytia *and *Hydra *having two paralogues whereas anthozoans have a single orthologue. Cnidarian sequences of gene sets II and V as well as sponge sequences of gene set IV share a unique insertion within the HMG domain (see red star at position 320 in Additional file 1). Among the SoxB-specific motives previously identified outside the HMG domain [26], only the "group B motif" located just downstream of the HMG domain is conserved in all members of the SoxB clade including the *Clytia *genes (Additional file 1). Other conserved motives, particularly the C-terminal motifs I and II shared by "subgroup B1" genes [26] (indicated in blue in additional file 1) are absent from *Clytia *SoxB genes.

The global phylogenetic arrangement of *SoxB *genes implies that the bilaterian paralogues originated from duplication events independent from those that produced the cnidarian paralogues. An important consequence is that no simple orthology relationship exists between individual cnidarian and bilaterian *SoxB *genes. For example, the vertebrate stemness master gene *Sox2 *has no specific orthologue in cnidarians, or in other terms, all cnidarian *SoxB *genes are equally orthologous to the vertebrate *Sox2 *gene and to any other vertebrate *SoxB *genes. The paraphyletic arrangement of cnidarian *SoxB *genes might indicate their origin from ancient duplications with subsequent losses of all but one paralogue in the bilaterian lineage, an interpretation also supported by the presence of sponge orthologues in gene set IV (Figure 1). The presence of the ctenophore gene *PpiSox3 *(for which expression data were described in [10]) nested within the clade of bilaterian *SoxB *paralogues is puzzling as it would imply that the diversification of bilaterian genes predated the ctenophore/bilaterian ancestor, with most ctenophore orthologues and all cnidarian ones having been lost. Alternatively, this strange position of *PpiSox3 *could be due to phylogenetic reconstruction artefacts or to sequence convergence.

Within group C, bilaterian and non-bilaterian genes segregated in two sister clades. Except for vertebrates and the ctenophore *Pleurobrachia pileus*, each species is represented by a single gene. The phylogenetic position of the two closely-related ctenophore genes (*PpiSox2 *and *12*) is unstable. They are found either sister-group to the other non-bilaterian *SoxC *genes (ML analysis; Figure 1), sister-group to all other group C genes (NJ analysis, not shown), or unclassified (not shown Bayesian analyses and [10]). Shinzato *et al. *[26] identified two SoxC-specific motives in the C-terminal region of the protein (in blue in additional file 2). The presence of these signatures cannot be assessed for *Clytia CheSox12 *because the sequence is too short. However, both motives are detectable in the other non-bilaterian sequences (from *Pleurobrachia, Acropora, Nematostella *and *Hydra*) falling into the SoxC clade in our phylogenetic analysis of the HMG domain (Figure 1; Additional file 2). This provides support for orthology between bilaterian and non-bilaterian SoxC genes.

A clade of bilaterian group F genes was recovered in all analyses but relationships of the non bilaterian *SoxF *genes are unclear. In particular, the sponge genes *EmuSox1 *and *AquSoxF *cluster with the *SoxF *clade in the NJ analysis (data not shown) but not in the ML (Figure 1) and Bayesian analyses (data not shown). Cnidarian genes do not form a monophyletic group within *SoxF*. No *SoxF *gene was detected in the hydrozoan *Hydra magnipapillata*, suggesting *Hydra*-specific loss of the *SoxF *group. A conserved SoxF motif (transcriptional activator domain) in the C-terminal region of the protein is clearly present in anthozoans [26], and is also detectable but only weakly conserved in *Clytia CheSox11 *(Additional file 3), reminiscent of the situation seen in *Ciona *(note furthermore that this motif is not detectable in *Drosophila *SoxF).

The topology within group E suggests that the common ancestor of eumetazoans had two paralogues, that one of them was lost independently in bilaterians and in ctenophores (according to the phylogeny of [22]), and that the other one was lost in *Hydra*. Indeed, group E is subdivided into two sister-clades, one containing only cnidarian genes (*CheSox5, HmaSox5 *and *NveSoxE2*), and the other one including two ctenophore paralogues, all bilaterian *SoxE *genes (*HSASox8, 9 *and *10, DmeSoxE *and *CinSoxE*), the *Clytia *gene *CheSox1*, two anthozoan genes (*NveSoxE1 *and *AmiSoxE1*) but no *Hydra *gene. Three conserved motifs were previously identified as specific for group E, although in fact they are shared only by a subgroup of SoxE sequences ("subgroup E1" in [26]). Of these, motif III is clearly identified in *CheSox1 *(as well as in the anthozoan proteins *Nve SoxE1 *and *AmiSoxE1*), but not in the ctenophore SoxE sequences (Additional file 4). Motif II is only weakly conserved at the metazoan level and its occurrence in *Clytia *(and other non-bilaterian) SoxE proteins is not obvious. Motif I is highly conserved in anthozoan SoxE proteins, and more weakly in ctenophore *PpiSox1 *and in the hydrozoan proteins *HmaSox5 *and *CheSox5*.

### Four *Clytia Sox *genes belonging to three distinct subgroups are expressed in stem cells of the medusa tentacle bulbs

**Figure 2:**
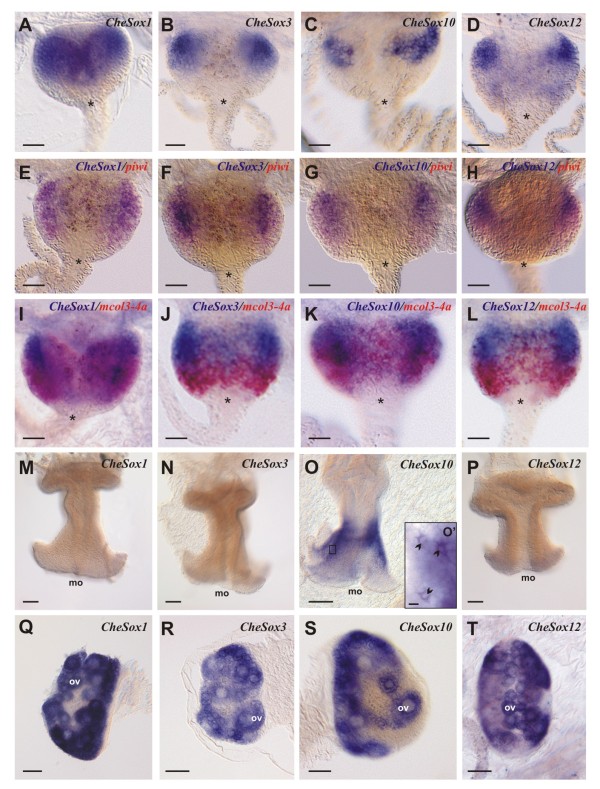
**Expression patterns of *CheSox1, CheSox3, CheSox10 *and *CheSox12 *in the medusa**. **(A-D) **Single *in situ *hybridisation in the medusa tentacle bulbs for *CheSox1 *(A), *CheSox3 *(B), *CheSox10 *(C) and *CheSox12 *(D). These four genes are expressed in two symmetrical patches at the proximal pole of the tentacle bulb. **(E-H) **Double *in situ *hybridisations of these four *Sox *genes (in blue) with the stem cell marker *Piwi *(in red) in the tentacle bulb. In the four cases, staining is homogeneously purple, indicating co-expression of the genes. **(I-L) **Double *in situ *hybridisations of the four *Sox *genes (in blue) with *Minicollagene 3-4 *(*mcol3-4a*) (in red), a marker of nematocyte differentiation. Most *CheSox1, CheSox3, CheSox10 *and *CheSox12 *positive cells do not express *mcol3-4a*. In A-L, tentacle bulbs are oriented with their proximal pole on the top; the star indicates the base of the tentacle. **(M-P) **Gene expression in the manubrium (lateral orientation, with mouth on the bottom, mo) for the same four *Sox *genes. No signal was detected for *CheSox1 *(M), *CheSox3 *(N) and *CheSox12 *(P) whereas *CheSox10 *(O) is strongly expressed in the ectoderm of the four longitudinal ridges of the manubrium. **(O') **detailed view of the area boxed in O. Black arrowheads point to stained cells, showing a typical neuronal morphology. **(Q-T) **Expression in the female gonad of *CheSox1 *(Q), *CheSox3 *(R), *CheSox10 *(S) and *CheSox12 *(T). These four genes are expressed in maturating oocytes (ov). Scale bars: A-L, 20 μm; M-T, 50 μm; O', 2 μm.

The four *Sox *genes *CheSox1 *(Group E), *CheSox3 *(Group B), *CheSox10 *(Group B), and *CheSox12 *(Group C) have highly similar expression patterns at the medusa stage in the interstitial stem cells of the tentacle bulbs (Figure 2A-L). These four *Sox *trancripts were detected in the proximal region of the bulb, near the bell margin, a region identified as a stem cell niche in previous work [31]. Their expression domains were restricted to two symmetrical patches at the tentacle bulb base (Figure 2A-D) and mimicked the expression pattern previously described for the stem cell marker *Piwi *[31]. Double *in situ *hybridisations were performed using two different marker genes, to gain more detailed indications concerning mRNA distribution along the axis of the tentacle bulb. Co-expression with *Piwi *was found for *CheSox1, CheSox3, CheSox10 *and *CheSox12 *(purple colour in Figure 2E-H). The *Minicollagen 3-4 *(*mcol3-4a*) gene encodes a component of the nematocyst capsule and is expressed in differentiating nematoblasts, the dominant cell type in tentacle bulb ectoderm (Figure 2I-L in red; [35]). Double *in situ *hybridisations with *mcol3-4a *(Figure 2A-D) indicate that the proximal domain where *CheSox1, 3, 10 *and *12 *are expressed (in blue) is clearly distinct from the more distal *mcol3-4a *expression domain (in red), with co-expression limited in each case to very few cells (like for *Piwi *and *mcol3-4a *in [35]).

### Other expression sites for *CheSox1, CheSox3, CheSox10 *and *CheSox12*

In the medusa, *CheSox10 *transcripts were also abundant in the distal third of the manubrium ectoderm, particularly along each of the four ridges (Figure 2O; higher magnification in Figure 2O'). *CheSox10 *expression in this area appears salt-and-pepper. The stained cells lack a capsule and thereby they are not nematocytes, their polygonal cell body bearing outgrowths evokes nerve cells (Figure 2O'), and their distribution along the four manubrium ridges closely matches a dense population of nerve cells revealed by anti-FMRFamide antibody staining (Additional file 5). Therefore, *CheSox10 *expression in the manubrium of the medusa is likely to be associated with a neuronal cell type. No signal could be detected for *CheSox1, 3 *and *12 *in the manubrium (Figure 2M, N, P). Finally, all four *So*x genes were strongly expressed in oocytes in the gonads of female medusae (Figure 2Q-T).

**Figure 3:**
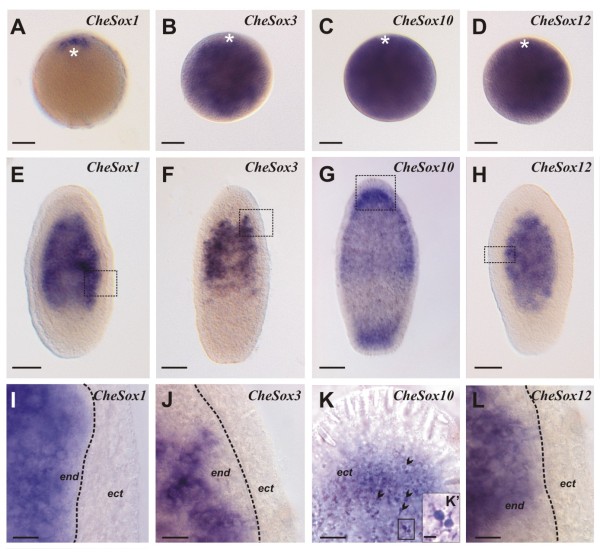
**Expression of *CheSox1, CheSox3, CheSox10 *and *CheSox12 *in non-fertilised eggs and two-day planulae**. **(A-D) **In non-fertilised eggs (animal pole on the top indicated by a white star), *CheSox1 *transcripts (A) are detected around the nucleus at the animal pole whereas *CheSox3 *(B), *CheSox10 *(C), *CheSox12 *(D) transcripts are homogeneously distributed throughout the egg cytoplasm. **(E-H) **Gene expression in two-day planula larvae. The stained cells are endodermal and their aspect is similar for *CheSox1 *(E), *CheSox3 *(F) and *CheSox12 *(H). In contrast, *CheSox10 *(G) is expressed in ectodermal basi-epithelial cells distributed throughout the oral-aboral axis but particularly concentrated at both poles. All planulae have the same orientation, with oral pole (= posterior pole) on the top. **(I-L) **Higher magnification views corresponding to the boxed areas in E to H. Stained cells in I, J and L have the typical aspect of interstitial cells. *CheSox10 *is expressed in small cells forming a dense basi-epithelial population (black arrowheads). The boundary between ectoderm (ect) and endoderm (end) is indicated by a dotted line in I, J, L. This boundary cannot be placed in K because the optical plane is more superficial (ectoderm throughout the picture). **(K') **Higher magnification picture corresponding of the boxed area in K, showing two neighbouring stained cells, with typical neuronal morphology. Scale bars: A-H, 50 μm; I-L, 10 μm; K', 2 μm.

Transcripts of these four *Sox *genes were also detected in the cytoplasm of the unfertilised eggs, indicating that these mRNA are maternally inherited (Figure 3A-D). *CheSox1 *transcripts (Figure 3A) appeared localised to the region around the nucleus at the animal pole, whereas the three other maternal transcripts were detected uniformly through the egg cytoplasm (Figure 3B-D).

In the planula larva, three genes *CheSox1 *(Figure 3E), *CheSox3 *(Figure 3F) and *CheSox12 *(Figure 3H) displayed similar expression patterns with transcripts detected in cells scattered throughout the endoderm (but not in the aboral-most region). Observation of these stained cells at higher magnification (respectively 3I to 3J and 3L) failed to reveal any particular characteristic that would point to differentiated cell types of the larva. These expression patterns closely resemble that of *ChePiwi *(Additional file 6) suggesting that *CheSox1, CheSox3 *and *CheSox12 *are expressed in larval interstitial stem cells. In contrast, *CheSox10 *larval expression was detected at the base of the ectoderm, particularly concentrated at the two poles of the larva (Figure 3G), which strongly evokes the planula nerve net, known to be basiepithelial and to extend throughout the ectoderm with higher nerve cell concentration at both poles (see immunostaining of the larval nerve net in Additional file 7). Additional arguments for a neuronal identity of the *CheSox10 *expressing cells in the planula are (i) transmission electron microscopy data indicating that no cell type other than nerve cells occur in the basal part of larval ectoderm (except scattered migrating nematoblasts) [29]; (ii) small size (about 2 to 4 μm) of these *CheSox10 *positive cells (diameter of neuronal cell bodies about 3 to 6 μm in immunostained preparations, Additional file 7); (iii) presence of thin extensions (neurites) on the *CheSox10 *expressing cells (visible at high magnification: Figure 3K').

### Five *Clytia Sox *genes belonging to distinct subgroups are implicated in nematogenesis in the medusa tentacle bulbs

**Figure 4:**
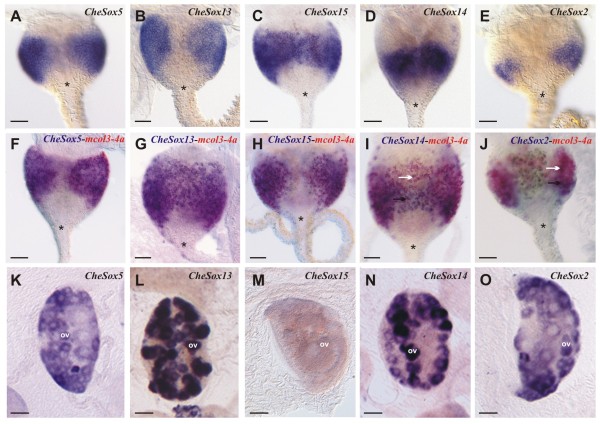
**Expression patterns of *CheSox5, CheSox13, CheSox15, CheSox14 *and *CheSox2 *in the medusa**. **(A-E) **Single *in situ *hybridisations showing that the five genes have crescent-shaped expression patterns in the ectodermal layer of the tentacle bulb. Staining is continuous on the inner face of the bulb but interrupted on the outer face, as typical for nematogenesis genes [35]. **(F-J) **Double *in situ *hybridisations with *Minicollagene 3-4 *(*mcol3-4a*) riboprobe reveals extensive co-expression (purple staining) with *CheSox5 *(F), *CheSox13 *(G) and *CheSox15 *(H) but only partial co-expression, in the most distal area of the bulb, with *CheSox14 *(I) and *CheSox2 *(J). All tentacle bulbs have the same orientation, with their proximal pole on the top and tentacle on the bottom (star: tentacle base). White arrows in I and J indicate cells expressing only *mcol3-4a *(red staining); black arrows in I and J indicate cells expressing both genes (purple staining). **(K-O) **Gene expression in the female gonads. Strong staining is observed in maturing oocytes (ov) for *CheSox5 *(K), *CheSox13 *(L), *CheSox14 *(N) and *CheSox2 *(O), while no expression was detected for *CheSox15 *(M). Scale bars: A-J, 20 μm; K-O, 50 μm.

Five *Clytia *genes *CheSox5 *(group E), *CheSox13 *(group B), *CheSox15 *(unclassified), *CheSox14 *(group B) and *CheSox2 *(group B) are expressed in the nematogenic ectoderm of the medusa tentacle bulbs (Figure 4). Their expression patterns are crescent-shaped and interrupted on the external side of the bulb (Figure 4A-E), as for all the nematogenesis genes described by [35]. The exact position of this crescent along the bulb proximo-distal axis differed among the five genes. *CheSox5 *(Figure 4A), *CheSox13 *(Figure 4B) and *CheSox15 *(Figure 4C) are expressed in a wide median zone spanning most of the bulb axis except the most proximal and distal regions, similar to *CheMcol3-4a *expression (see Figure 2I- L in red, and [35]). *CheSox14 *(Figure 4D) and *CheSox2 *(Figure 4E) expression was detected in a more restricted and distal area. Double *in situ *hybridisation with the minicollagen *CheMcol3-4a *ripoprobe (Figure 4F-J) revealed its extensive co-expression with *CheSox5 *(Figure 4F), *CheSox13 *(Figure 4G) and *CheSox15 *(Figure 4H) but only partial co-expression in the most distal part of the bulb with the two other genes *CheSox14 *(Figure 4I) and *CheSox2 *(Figure 4J). According to the model presented in [35], these data indicate that these five *Sox *genes are expressed in differentiating nematoblasts, with *CheSox5, CheSox13 *and *CheSox15 *expressed during a large time window, and *CheSox14 *and *CheSox2 *only expressed during the latest phase of nematogenesis. Transcripts of *CheSox5, CheSox2, CheSox13 *and *CheSox14*, but not *CheSox 15*, were also detected in developing oocytes in the gonad (Figure 4K-O).

### Expression of *CheSox5, CheSox13, CheSox15, CheSox14 *and *CheSox2 *in eggs and planulae

**Figure 5:**
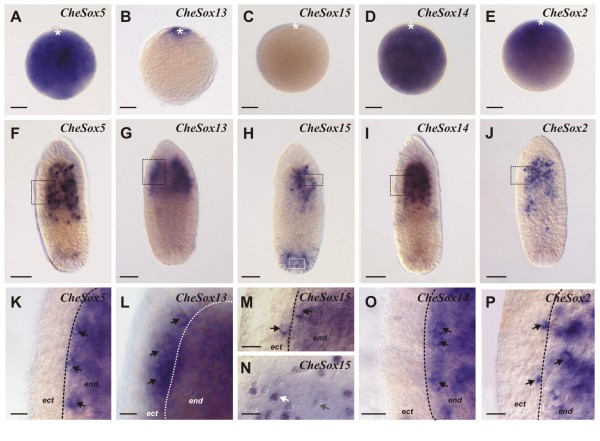
**Expression of *CheSox5, CheSox13, CheSox15, CheSox14*, and *CheSox2 *in non-fertilised eggs and two-day planulae**. **(A-E) **In non-fertilised eggs (animal pole on the top indicated by a white star), *CheSox5 *(A), *CheSox14 *(D) and *CheSox2 *(E) are homogeneously expressed throughout the egg cytoplasm. Restricted expression around the nucleus at the animal pole was obtained for *CheSox13 *(B), while no transcripts were detected for *CheSox15 *(C). **(F-J) **Expression in 2-day planula (oral pole on the top). *CheSox5 *(F), *CheSox14 *(I) and *CheSox2 *(J) are expressed in endodermal cells concentrated in the oral half of the larva. *CheSox13 *transcripts (G) are present in basi-epithelial ectodermal cells, essentially in the oral half of the planula. *CheSox15 *(H) is expressed at two different sites: in ectodermal cells at the aboral pole, and in ectodermal and endodermal cells of the oral half of the larva. **(K, L, M, O, P) **Higher magnification views of the areas boxed in black in F to J reveal the presence of a round non-stained capsule within the cytoplasm of positive cells, for each of the five *Sox *genes (black arrows), indicating that these cells correspond to maturating nematoblasts. **(N) **Higher magnification view of the area boxed in white in H. This area is rich in cells whose cytoplasm is densely filled with granules, some of these cells being stained with the *CheSox15 *riboprobe (white arrow) while others are deprived of any signal (grey arrow). The boundary between the ectodermal (ect) and endodermal (end) layers is indicated by a dotted line in K, L, N, O, P. Scale bars: A-J, 50 μm; K-P, 10 μm.

In unfertilised eggs (Figure 5A-5E), an *in situ *hybridisation signal was detected throughout the cytoplasm for *CheSox5 *(Figure 5A), *CheSox14 *(Figure 5D) and *CheSox2 *(Figure 5E), and was apparently homogeneous except for *CheSox2*, whose transcripts appeared to be distributed in an animal-vegetal gradient. In contrast, *CheSox13 *transcripts (Figure 5B) were only detected at the animal pole around the nucleus (similar to *CheSox1*; Figure 3A), and no maternal transcripts were detected for *CheSox15 *(Figure 5C).

At the planula stage, *CheSox5 *(Figure 5F), *CheSox14 *(Figure 5I) and *CheSox2 *(Figure 5J) exhibited similar expression patterns. They were mainly expressed in endodermal cells concentrated in the posterior (oral) half of the larva, with a few positive ectodermal cells also detected. The stained cells are maturating nematoblasts, as indicated by the presence in their cytoplasm of a rounded capsule, observed at high magnification (Figure 5K, O, P, black arrows). The identification of these unstained circles as capsules (and not nuclei) was confirmed by their lack of fluorescence after DAPI staining of the cell nuclei (Additional file 8 A-A", D-D", E-E"). Some of these stained cells are arranged in clusters (Figure 5O) and others are isolated (see for example Figure 5P). *CheSox13 *transcripts (Figure 5G) are essentially present in the posterior (oral) part of the planula, in basi-epithelial ectodermal cells. Observation at high magnification revealed that transcripts are distributed around a capsule, again suggesting expression of the gene in differentiating nematoblasts (Figure 5L, and 5see absence of DAPI fluorescence in these capsules in additional file 8 B-B"). *CheSox15 *was expressed at two different sites within the planula (Figure 5H). In the posterior half of the larva, *CheSox15 *was expressed in ectodermal (basi-epithelial) and endodermal nematoblasts (Figure 5M, and 5see absence of DAPI fluorescence in these capsules in additional file 8 C-C"). At the anterior pole, *CheSox15 *transcripts were detected in ectodermal glandular cells containing dense granules in their cytoplasm (Figure 5N).

### *CheSox11 *(Group F) is a marker of endodermal cells

**Figure 6:**
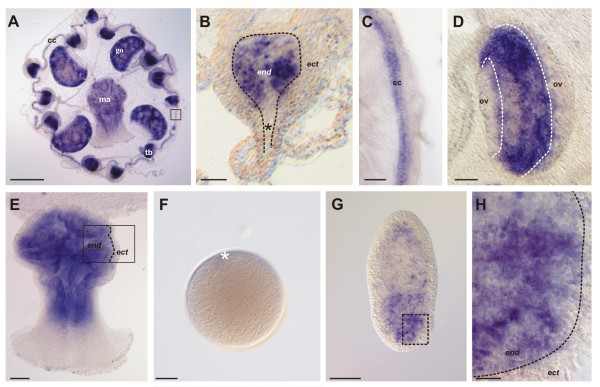
**Expression of *CheSox11 *(Group F) in the medusa, non-fertilised eggs and two-day planulae**. (**A**) General view of a mature female medusa showing endodermal distribution of *CheSox11 *transcripts. (**B**) *CheSox11 *is expressed in a subpopulation of endodermal cells of the tentacular bulbs (limit between ectoderm and endoderm indicated by the dotted line). (**C**) Higher magnification view of the area boxed in A, showing *CheSox11 *expression in the endodermal circular canal (cc). (**D**) Endodermal cells of the radial canal (delineated by the white dotted line) which crosses the gonad also express *CheSox11*. Note that the gene is not expressed in the gonad itself (maturating oocytes, ov, are unstained). (**E**) *CheSox11 *expression in the manubrium endoderm (limit between ectoderm and endoderm indicated by the dotted line in the rectangle). (**F**) No expression was detected in unfertilised eggs (animal pole on the top, white star). (**G**) Endodermal staining in a two-day planula particularly concentrated in the anterior half. (**H**) Higher magnification view of the area boxed in G reveals the diffuse aspect of the staining. cc: circular canal, ect: ectoderm, end: endoderm, go: gonad, ma: manubrium, ov: maturing oocytes, tb: tentacle bulb. Scale bars: A, 50 μm; B, 20 μm; C, 10 μm; D to F, 50 μm, G, 50 μm; H, 10 μm.

*CheSox11 *is the only *Sox *gene expressed in the endoderm of the medusa (Figure 6A). Expression of this gene was observed in endodermal cells of the tentacle bulbs (Figure 6B), in the endodermal circular canal (cc in Figure 6A, B) as well as in radial canals, particularly at the level of the gonads (but not in germ line cells) (Figure 6D) and in endodermal cells of the manubrium (Figure 6E). No expression was detected in unfertilised eggs (Figure 6F). At the planula stage, expression was scattered throughout the endoderm but with higher intensity in the anterior half of the larva (Figure 6G). We could not identify precisely in which cell type *CheSox11 *is expressed. An expression of this gene in interstitial cells cannot be excluded, although this seems unlikely given that *Piwi *(marker of interstitial cells) is mainly expressed in the posterior half of the planula (see Additional file 6), whereas *CheSox11 *expression is maximal in the anterior half. Given the absence of any particular characteristic (for example, capsule, neurites...) of the *CheSox11*-positive cells, they most probably correspond to banal epithelial cells of the endoderm.

**Figure 7:**
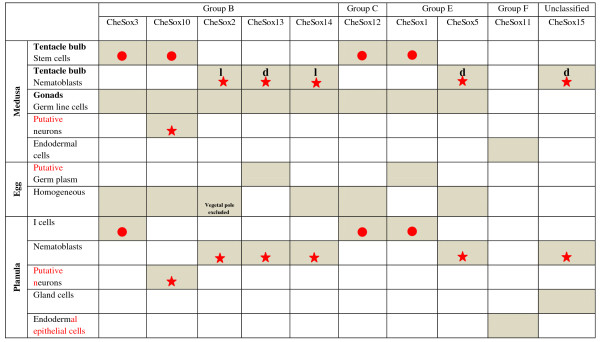
**Summary of *Clytia Sox *expression patterns**. Expression is indicated by grey boxes. When possible, symbols were used to label genes associated with either undifferentiated state (red circle) or cell differentiation (red star). l: late differentiation, d: throughout differentiation.

All expression patterns are summarised in Figure 7.

## Discussion

### The general characteristics of *Clytia Sox *gene expression suggest evolutionary conservation at the gene family level

*Clytia Sox *genes can be classified in three expression groups (see Figure 7 summarising all expression data from this study): (i) endodermal expression (only for the group F gene *CheSox11*; see below); (ii) localised expression in stem cells / undifferentiated progenitors (of the medusa and/or the planula larva: *CheSox1, CheSox3, CheSox10, CheSox12*); (iii) expression in differentiating or differentiated cells with neuro-sensory identity (putative larval and medusa neurons for *CheSox10*, nematoblasts for *CheSox2, CheSox13, CheSox14, CheSox5 *and *CheSox15*). The neuro-sensory nature of cnidarian nematocytes is supported by arguments from cytology (presence of synapses, neurites, and of an apical mechanosensory complex, reviewed in [36]), physiology (electrical recording of action potential-like transmembrane currents, [37]) and developmental biology (expression in nematoblasts of conserved neurogenic genes, reviewed in [36]). Our results indicate that along the nematocyte cell lineage in the medusa tentacle bulbs, multiple *Sox *genes act at one side or the other of the stem cell/progenitor vs. differentiating cell equilibrium. Interesting perspectives for future experimental studies in *Clytia *include the identification of their molecular partners, as well as functional assays to determine the roles played by these various *Sox *genes in stem cell maintenance, progenitor proliferation, and cell differentiation.

These general features of *Sox *gene expression in *Clytia *are likely to reflect ancestral properties of the *Sox *family at the level of the eumetazoan clade. This includes the recurrent association of *Sox *genes with stem cells and with neuro-sensory cell differentiation, well documented in vertebrates [3,13]. In the ctenophore *Pleurobrachia*, two *Sox *genes (*PpiSox2 *and *PpiSox12*) are expressed at various body locations [10] in cell populations that have been recently characterised as pools of somatic stem cells expressing genes like *Piwi *and *Vasa *[27]. It is important to underline that these *PpiSox2/PpiSox12*-expressing stem cells give rise to a variety of cell types, and not exclusively to neural or sensory cells (*for example*, muscle cells for the stem cells of the tentacle root median ridge). All the remaining *Sox *genes for which expression was characterised in the adult ctenophore (except the *SoxF *orthologue) were expressed in ectodermal differentiated cells with a clear or probable neural or sensory identity (for example, *PpiSox3 *in ganglion-like structures of the polar fields called the Z bodies; *PpiSox6 *in the ciliated "polster cells" of the comb rows, which are known to have mechanosensory properties; see [10]). There is also good evidence in the ctenophore that at least in some contexts, several *Sox *genes are differentially expressed along the same cell lineage in stem cells and in their differentiated progeny (*PpiSox2/PpiSox12 *vs. *PpiSox3 *in the polar fields; *PpiSox2/PpiSox12 *vs. *PpiSox6 *in the comb rows). *Sox *gene expression in other non-bilaterian models has been less precisely characterised in terms of progression along cell lineages, but several *Sox *genes in *Nematostella *and *Acropora *have "salt-and-pepper" expression patterns thought to correspond to neuro-sensory cells [8,26].

The germline probably represents an additional context in which *Sox *family genes have been involved since the eumetazoan common ancestor. Eight of the *Sox *genes investigated here in *Clytia*, belonging to groups B, C and E, were found expressed in germline cells of the female medusa gonad (no data on male medusae). In the ctenophore *Pleurobrachia pileus, PpiSox2 *and *PpiSox12 *(but not the other *Sox *genes) are expressed in the female germline [10]. In bilaterians, several *Sox *genes have important function in the germline. For example, in vertebrates *Sox3 *(group B) is crucial for oocyte development and spermatogenesis [38]. Other instances of *Sox *genes expressed in the germline include *Sox30 *(unclassified) in human [39,40] and *Dichaete *(group B) in *Drosophila *oogenesis [41]. In the honeybee (*Apis mellifera*), four of the eight *Sox *genes (*AmSoxB1, AmSox21b, AmSoxF, AmSoxD*) are expressed in nurse cells and/or in oocytes, whereas *AmSoxE1 *and *AmSoxE2 *expression is testis-specific (RT-PCR analyses; [42]).

The *SoxF *group seems to represent a special case among the *Sox *subfamilies as its members do not have the expression characteristics outlined above, but instead are consistently and widely expressed in endoderm derivatives, in all non-bilaterian animals where they have been investigated so far. Thus, the *Clytia SoxF *orthologue *CheSox11 *is expressed in the planula endoderm, and in most endodermal structures of the medusa (tentacle bulb endoderm, canals of the gastrovascular system, manubrium endoderm). Likewise, *SoxF *genes have an exclusively endodermal expression in the ctenophore *Pleurobrachia *(throughout the gastro-vascular system; [10]) and in the anthozoan cnidarians *Nematostella *[8] and *Acropora *[26]. The case of *Hydra *is particular since the *SoxF *group has apparently been lost in this lineage. In vertebrates, important functions in endoderm development are documented for *SoxF *genes [43]. Therefore, *SoxF *endodermal expression probably dates back to a common ancestor of eumetazoans, although the cellular functions of these genes are unclear. Additional functions of the vertebrate *SoxF *genes (for example, in cardiogenesis and angiogenesis), and the involvement of *Drosophila SoxF *in peripheral nervous system and wing development, but not in endoderm development [42] are most easily interpreted as derived situations.

### Comparison of *Sox *gene expression across *Clytia *life stages

In *Clytia, Sox *genes tend to have consistent expression properties across life stages, in terms of cell type or cell lineage stage, but with some notable exceptions. Among the four *Sox *genes expressed in stem cells of the medusa tentacle bulb, three were expressed in the planula larva in endodermal cell patches interpreted as groups of interstitial stem cells. These three *Sox *genes (*CheSox3, CheSox12 *and *CheSox1*) thereby seem to behave as stem cell markers across life stages in *Clytia *(although data are lacking for the polyp stage, whose stem cells are located in the stolons of the colony). The five genes (*CheSox5, CheSox13, CheSox15, CheSox14 *and *CheSox2*) expressed in medusa tentacle bulb nematoblasts were all expressed in larval nematoblasts as well, and the *CheSox10 *gene, which seems to be expressed in a subpopulation of medusa neurons, also had expression associated with neural cells in the planula larva. However, expression of *CheSox10 *was detected in stem cells of the medusa but not of the planula larva.

Recently, we found that a series of RNA regulatory genes (*Piwi, Vasa, PL10, Nanos*) all expressed in stem cells of the *Clytia *medusa and planula have maternally-inherited transcripts localised in a germ-plasm-like structure closely associated with the oocyte nucleus ([44]; Leclère *et al. *submitted). These transcripts appear to be inherited through cleavage stages by a subset of blastomeres that migrate into the endodermal region upon gastrulation. Subsequently, the expression of these genes was detected in larval interstitial cells. This suggests that interstitial cells are specified by a mechanism akin to "preformation" of the germline by inheritance of maternal determinants as known for many bilaterians [45-47].

Contrary to RNA regulatory proteins like Piwi and Vasa and their mRNAs, the *Sox *are not known as germ plasm components in bilaterians. It was therefore surprising to see that mRNAs of two *Clytia Sox *genes (*CheSox13 *and *CheSox1*) were concentrated in a restricted area of the egg cytoplasm around the nucleus, closely resembling the mRNA distribution of *Piwi, Vasa *and other typical "germ plasm genes" ([44]; Leclère *et al. *submitted). However, for *Sox *genes there is no correlation between expression in stem cells of the medusa and planula, and association of mRNAs with the putative germ plasm in the egg. The stem cell markers *CheSox3 *and *CheSox12 *are expressed maternally but their mRNA are homogeneously distributed in the egg cytoplasm, and conversely *CheSox13 *has germ plasm-like expression in the egg, but in the larva and in the medusa it is expressed in nematoblasts, not in stem cells. Only *CheSox1 *cumulates expression in the putative germ plasm of the egg and expression in stem cells of the planula larva and of the medusa. A possible interpretation of these observations could be that only part of the regulatory genes acting in interstitial cells has their mRNAs present in the egg putative germ plasm, and/or that the later might have functions others than the specification of interstitial stem cells during development.

### Strong variability of expression characteristics between cnidarian *Sox *orthologues

While orthology relationships of *Sox *genes at the metazoan level are generally poorly supported and sometimes confused, relationships between genes from different cnidarian species are much clearer. In addition, expression data at a comparable life stage (planula larva) are now available for three different cnidarian species.

Except for *SoxF *genes (discussed above), the expression domains in the planula larva of orthologous *Sox *genes between different cnidarian taxa are strikingly different. For example, while the expression of *CheSox10 *(group B, gene set II, see Figure 1) is restricted to ectodermal nerve cells concentrated at both poles of the *Clytia *planula, its orthologue in *Nematostella *(*NveSoxB2*) is expressed in both germ layers, ectoderm and endoderm (Figure 3I in [8]), without any particular concentration of the transcripts towards the planula poles, and the *CheSox10 *orthologue in the coral *Acropora *(*AmiSoxBa*) has its transcripts restricted to the ectoderm at the aboral pole [26]. However, "salt-and-pepper" expression observed for the *Nematostella *and *Acropora *orthologues of *CheSox10 *suggests that these genes might be expressed in neural cells like in *C. hemisphaerica*, and thereby different spatial distribution of the transcripts in the planulae of the different species might simply reflect different architectures of the nervous system. Transcript distribution across the planula larva similarly differs for the two other cnidarian *SoxB *group: *CheSox2 *(group B "gene set IV", Figure 1) is expressed in the endoderm throughout the oral half of the *C. hemisphaerica *planula, while the expression of its orthologue *NvSox2 *(Figure 3HH in [8]) in *N. vectensis *is restricted to the ectoderm at the oral pole, and the same holds true for "gene set V" of the B group between *C. hemisphaerica *(*CheSox 13 *and *CheSox14*) and *N. vectensis *(*NveSox3*, Figure 3OO in [8]; no data for these genes in *Acropora*). All three *C. hemisphaerica *genes of sets IV and V are expressed in the larval nematoblasts, and the sea anemone patterns are perhaps comparable at this cellular level, but the cell types where *NveSox2 *and *NveSox3 *are expressed were not described in [8].

In the case of *SoxC *genes, there is clear disparity of expression characteristics at the cellular level, the *C. hemisphaerica CheSox12 *gene being expressed in larval interstitial stem cells, whereas in both anthozoans its orthologues (*NveSoxC *for *N. vectensis *and *AmiSoxC *for *A. millepora*) have salt-and-pepper expression in the planula ectoderm, and [26] provided convincing evidence that *AmiSoxC *is expressed in differentiated sensory neurons. *SoxE *orthologues have endodermal expression in the planula larva of the three compared cnidarian species, but while in *C. hemisphaerica *this expression localises to interstitial stem cells, there is no information about the *SoxE*-expressing cell type in *N. vectensis *and *A. millepora*. In conclusion, there is strong variability of orthologous *Sox *gene expression between cnidarian species. Notably, in at least one case (group C), the same gene is expressed in stem cell/progenitors in the hydrozoan *C. hemisphaerica*, but in differentiated cells in the anthozoans *N. vectensis *and *A. millepora*.

### Inconsistent distribution of gene expression/function characteristics across the *Sox *gene phylogeny

Published data on *Sox *gene expression and function in various animal taxa are more or less easy to interpret in terms of cellular state along cell lineages, depending on the characteristics of each animal model and on the main focus of each particular study. In Figure 1, we used symbols to label genes associated with either undifferentiated state or cell differentiation. We decided to highlight only genes from the three animal taxa for which relevant data is available for a substantial diversity of *Sox *genes: mammals (expression and function data reviewed in [2,3]; see [48] for mammalian *Sox18*); the ctenophore *Pleurobrachia pileus *(expression data from [10]) and *Clytia hemisphaerica *(expression data from this study). Data from other experimental models such as *Drosophila melanogaster *and *Caenorhabditis elegans *have not been mapped on the tree, because in most cases it was unclear whether the genes were associated with stem cells/undifferentiated progenitors, or with differentiated or differentiating cells.

Even when considering this limited species sampling, the distribution of character states across the tree (Figure 1) clearly indicates that gene expression/function characteristics with respect to cellular state are totally inconsistent with the gene phylogeny. Non-bilaterian *Sox *genes known to be expressed in stem cells or progenitors, and mammalian *Sox *genes known to maintain cells in undifferentiated state, are scattered throughout the *Sox *family tree. The same holds true for non-bilaterian *Sox *genes expressed in differentiating or differentiated cells and mammalian *Sox *genes promoting cell differentiation.

Not only each of the main *Sox *group (other than *SoxF*) contains genes from both categories, but also many individual genes belong to one category or the other in the same species, depending on the developmental context. For example, the mammalian *Sox1, Sox2, Sox3 *genes (members of the *SoxB *group) have fundamental roles in the maintenance of neural progenitors in the neural plate, but they also act in the terminal differentiation of several neuronal subtypes later on during development of the mammalian embryo ([2,3]; see [48] for mammalian *Sox18*). The *C. hemisphaerica CheSox10 *gene is expressed in stem cells / undifferentiated progenitors of the medusa tentacle bulbs, but also in differentiated neurons of the medusa manubrium and of the planula larva. Similar ambivalent involvement at both sides of the equilibrium in different contexts can also be suspected for some of the ctenophore *Sox *genes, for example *PpiSox1 *and *PpiSox3*, both expressed in differentiated neuro-sensory cells (respectively, of the apical organ floor and of the polar fields) but also in particular cell populations of the tentacle root (the aboral external cell masses) thought to be stem cells [27].

## Conclusions

A contrasted picture of the evolutionary conservation of *Sox *gene expression and function emerges from comparative data on bilaterian and non-bilaterian animals. At the global scale of the *Sox *family, genes tend to be involved either in maintenance of undifferentiated state in stem cells or progenitors, or in the differentiation of various types of neuro-sensory cells. This does not apply to the *SoxF *group, whose expression was probably associated with the endoderm in the common ancestor of eumetazoans. A high degree of evolutionary plasticity with respect to involvement in either stem cells/progenitors or differentiating(ed) cells is observed for the other *Sox *groups when comparing gene expression data (i) across *Sox *groups at the metazoan scale; (ii) between orthologous genes at a comparable life stage (planula larva) in different cnidarian species; (iii) for the same gene in a given species under different embryological, life stage or histological contexts.

This apparently paradoxical situation, with evolutionary conservation of a stereotyped set of functions at the gene family scale, but no conservation at the scale of the orthology groups, might reflect constraints acting at the levels of the transcriptional regulation networks, and of the physical interactions between *Sox *proteins and their partners. Indeed, it is the pair formed by a *Sox *protein and its partner transcription factor that determines the set of target genes for transcriptional regulation [6]. Under some circumstances, the *Sox *component of such a pair might happen to be exchanged with another *Sox *protein (possibly from another *Sox *group) without affecting the set of target genes (and thereby, the cellular state) controlled by the pair. This model could explain at the same time evolutionary stability of a set of functions at the gene family level, and frequent functional switches affecting individual *Sox *genes during evolution. A switch in the role could also come from changing from a repressor to an activator activity whilst keeping the same targets. More data on the expression, function and interactions of *Sox *genes and their partners in a wide sampling of non-bilaterian and bilaterian metazoans are required for testing these hypotheses.

## Materials and methods

### Survey for *Clytia hemisphaerica *Sox genes

In a previous work [9], nine partial *Sox *gene sequences (*CheSox1 *to *CheSox9*) were identified by RT-PCR amplification in *Clytia hemisphaerica *and one additional sequence (*CheSox10*) was detected in a preliminary survey of about 10,000 *Clytia hemisphaerica *ESTs. Recently, a larger data set of *Clytia hemisphaerica *ESTs (about 90,000) and full-length cDNAs (about 8,000) was sequenced at the Genoscope (Evry, France) (see [28]). These transcriptome data were searched by TBLASTN using the HMG domain of *CheSox1*. Some of the sequences previously identified by PCR (*CheSox2, CheSox3, CheSox5*) were retrieved in these searches. In addition, we could recover five new *Sox *genes, named *CheSox11 *to *CheSox15*. The *CheSox1 *sequence was extended by 3'RACE-PCR as described in [10].

### Alignment and phylogenetic analyses

The dataset was built using complete *Sox *gene repertoires of selected bilaterian species (*Homo sapiens, Ciona intestinalis, Caenorhabditis elegans *and *Drosophila melanogaster*) as well as sequences from non-bilaterian lineages. Anthozoan cnidarians were represented by the full *Sox *gene repertoire of the sea anemone *Nematostella vectensis *(14 *Sox *genes identified by [8]) and 6 published *Sox *genes sequences from the coral *Acropora millipora *[26]. To the *Sox *gene sequences available in GenBank for the hydrozoan *Hydra magnipapillata *(*HmaSox10 *XM-002154334, *HmaBb *XM-002160022 and *HmaB1 *XM-002161342), we added eleven additional *Sox *genes (named here *HmaSox1 *to *HmaSox9 *and *HmaSox11-HmaSox12; *note that the name *HmaSox10 *was already attributed) recovered from blast search (TBLASTN) against the hydra genome http://www.ncbi.nlm.nih.gov/genome/seq/BlastGen/BlastGen.cgi?pid=12875[49]. Our alignment thus comprises a total of 14 *Sox *genes for *Hydra magnipapillata*.

For *Clytia hemisphaerica*, only sequences containing a complete or almost complete HMG domain were included in the alignment (*CheSox1, 2, 3*, 5, *10, 11, 12, 13, 14 *and *15*) and shorter sequences were left apart. To complete this gene sampling, we added ctenophore *Sox *genes from *Pleurobrachia pileus *(*PpiSox1, 2, 3, 4, 6, 8 *and *12 *[10]) as well as available *Sox *gene sequences from demosponges: four sequences from *Amphimedon queenslandica *[11,12] and three from *Ephydatia muelleri *(also elongated by 3' RACE-PCR). A representative sampling of non-*Sox *HMG domains was selected as an outgroup [9,10].

The alignment was done automatically using MUSCLE [50] and then slightly corrected manually in BioEdit [51]. The identification of conserved blocks outside from the HMG domain (Additional files 1 to 4) was based on Shinzato *et al. *[26]. For phylogenetic analyses, only the HMG domain (79 aa) was taken into account (see alignment used for the analyses in Additional file 9). There were no missing data, except for *Clytia hemisphaerica CheSox1, Ephydatia muelleri EmuSox1-3 *and three *Pleurobrachia pileus *sequences (*PpiSox2, 3 *and *12*), for which the 11 N-ter amino-acids are lacking and were scored as missing data.

Phylogenetic analyses were carried out from the amino-acid alignment by Maximum-Likelihood (ML) using the PhyML program [52] with the JTT model of amino-acid substitution and the same parameters as in [9,10]. Distance Neighbour-Joining (NJ) analysis was also performed on the same alignment with 1,000 bootstrap replicates using PAUP.4.b3 [53]. We also performed a Bayesian phylogenetic analysis with Mr Bayes [54] under the JTT model, with 5,000,000 generations sampled every 100 generations and four chains. Convergence was reached before 2,000,000 generations; a majority rule consensus of 30,000 trees was produced and posterior probabilities were calculated from this consensus. ML and NJ bootstrap values higher than 50% and Bayesian posterior probabilities are indicated on the ML tree (Figure 1).

### Animal collection and *in situ *hybridisation

Medusae, eggs and larvae of *Clytia hemisphaerica *were obtained in the laboratory by culture of *Clytia hemisphaerica *colonies established from polyps provided by Evelyn Houliston (Villefranche-sur-mer) as previously described [55], except that artificial seawater (Reef Crystals^®^) was used. Medusae were left unfed during two days before fixation.

All stages were fixed for one hour at 4°C in 3.7% formaldehyde, 0.2% glutaraldehyde, PBT 1X (10 mM Na2HPO4, 150 mM NaCl, pH7.5, 0.1% Tween 20). DIG-labelled antisense RNA probe synthesis and *in situ *hybridisation were carried out as previously described [55] with some modifications. The Proteinase K treatment was extended (30 mn instead of 10 mn) and the hybridisation step lasted 48 to 72 hours instead of overnight. After postfixation and DAPI staining [56], samples were mounted in Citifluor^®^. Double *in situ *hybridisation was performed as described in [35]. DIC images were obtained with an Olympus BX61 microscope using Q-imaging Camera with Image Pro plus^® ^software (Mediacybernetics).

### Immunofluorescence

Medusae and planulae were incubated in 4% paraformaldehyde in phosphate-buffered saline (PBS) (10 mM Na_2 _HPO_4_, 150 mM NaCl, pH 7.5). After fixation for 30 minutes at 4°C, samples were washed several times in PBS, dehydrated through a graded series of ethanol and stored in methanol at -20°C. Immunofluorescence experiments were done as described previously [10]. Two primary antibodies were used to visualise the nerve net: a rat monoclonal anti-tyrosylated α-tubulin or YL1/2 antibody (1:1000 dilution, Serotec) for the planula larva, and a rabbit polyclonal anti-FMRFamide (1:1000 dilution, Abcam) for the medusa. Samples were incubated overnight at 4°C with the appropriate secondary antibodies: Alexa Fluor^® ^568 goat anti-rat IgG or Alexa Fluor^® ^488 goat anti-rabbit IgG (Molecular probes). Dilutions of primary and secondary antibodies were made using 1X PBS containing 0.01% Triton-X100 (PBST). All samples were finally incubated with DAPI (1 μg/ml) for 15 mn for DNA staining, and then washed three times for 15 mn in PBST.

## Abbreviations

BS: bootstrap support; cDNA: complementary desoxyribonucleic acid; DAPI: 4',6-diamidino-2-phenylindole; EST: expressed sequence tag; HMG: high-mobility group; JTT: a matrix of amino-acid substitution rates proposed by Jones, Taylor and Thornton; ML: maximum likelihood; mRNA: messenger ribonucleic acid; NJ: neighbour-joining; PBS: phosphate buffer alkaline; PCR: polymerase chain reaction; RACE-PCR: rapid amplification of cDNA-ends by polymerase chain reaction; RT-PCR: polymerase chain reaction on retro-transcribed DNA fragments.

## Competing interests

The authors declare that they have no competing interests.

## Authors' contributions

MJ isolated the genes, did the phylogenetic analyses, peformed the expression analyses, and drafted the manuscript. MM designed the study, took part in the genome searches, and participated in manuscript preparation. EQ helped to design the study and helped in manuscript preparation. All authors read and approved the final manuscript.

## Supplementary Material

Additional file 1 **Alignment of group B Sox amino-acid sequences**. The HMG domain is underlined in red. The red star indicates the insertion (at position 75 in the HMG domain) which characterises some of the sponge and cnidarian genes (see text). The "group B motif" and conserved regions I and II identified by Schinzato *et al. *[26] are underlined in blue. Species names are abbreviated as follows: *Acropora millepora, Ami; Amphimedon queenslandica, Aqu; Caenorhabditis elegans, Cel; Ciona intestinalis, Cin; Clytia hemisphaerica, Che; Drosophila melanogaster, Dme; Ephydatia muelleri, Emu; Hydra magnipapillata, Hma; Homo sapiens, HSA; Nematostella vectensis, Nve; Pleurobrachia pileus, Ppi*. Click here for file

Additional file 2 **Alignment of group C Sox amino-acid sequences**. Legend as for Additional file 1. Click here for file

Additional file 3 **Alignment of group F Sox amino-acid sequences**. Legend as for Additional file 1. Click here for file

Additional file 4 **Alignment of group E Sox amino-acid sequences**. Legend as for additional file 1. Click here for file

Additional file 5 **Dense nerve net on the manubrium ridges revealed by FMRFamide immuno-staining**. **(A) **FMRFamide immunofluorescence staining of a manubrium showing marked/strong condensation of the nerve net along the four longitudinal ridges. **(B) **Higher magnification view of the region indicated by the box in A, showing the aspect of the nerve net. Scale bars: A, 50 μm; B-C, 20 μm. Click here for file

Additional file 6 ***Piwi *expression in the planula larva**. **(A) **Expression pattern of *Piwi *in a two-day-old planula (oral pole on the top). **(B) **Higher magnification view showing the distribution and aspect of the interstitial stem cells. ect: ectoderm; end: endoderm. Scale bars: A, 50 μm; B, 10 μm. Click here for file

Additional file 7 **Distribution of the nerve net in two-day-old planula detected using YL1/2 antibody**. **(A) **Superficial view (optical plane on the basal part of the ectodermal epithelium). **(B) **Deeper view of the same specimen (optical plane crossing the larval endoderm and cavity). (**C**) Higher magnification view of the YL1/2 staining (in red) with Dapi counter-staining (in blue) showing the distribution and aspect of nerve cell bodies (white arrowheads) and neurites. Oral pole is on the top for all pictures. Scale bars: A-B, 50 μm; C, 10 μm. Click here for file

Additional file 8 **Detailed views of the capsules and DAPI-stained nuclei in *CheSox5, CheSox13, CheSox15, CheSox14 *and *CheSox2 *expressing nematoblasts**. (**A-E**) Transcript distribution after ISH, viewed at high magnification, for the five *Sox *genes cited above. The signal is concentrated around unstained maturating capsules (black arrows). (**A'-E'**) Dapi counter-staining of (A-E). The white stars indicate DAPI-stained nuclei of the cells containing ISH signal in (A-E). (A"-E") Merged pictures combining the ISH signal (in black and white, pictures A-E) and the DAPI signal (in red, pictures A'-E'). Maturating nematoblast capsules are indicated by white arrows and nuclei of the corresponding cells by white stars. Scale bars: A-E": 2 μm. Click here for file

Additional file 9 **Alignment of *Sox *HMG domain sequences used for the phylogenetic analyses (in Fasta format)**. Click here for file
