# Supplementary material for: Multiple Sox genes are expressed in stem cells or in differentiating neuro-sensory cells in the hydrozoan Clytia hemisphaerica
Source: EvoDevo. 2011 Jun 1;2:12. doi: 10.1186/2041-9139-2-12 (PMC3120710; doi:10.1186/2041-9139-2-12)
Supplement: Additional file 1 — Alignment of group B Sox amino-acid sequences. The HMG domain is underlined in red. The red star indicates the insertion (at position 75 in the HMG domain) which characterises some of the sponge and cnidarian genes (see text). The "group B motif" and conserved regions I and II identified by Schinzato et al. [26] are underlined in blue. Species names are abbreviated as follows: Acropora millepora, Ami; Amphimedon queenslandica, Aqu; Caenorhabditis elegans, Cel; Ciona intestinalis, Cin; Clytia hemisphaerica, Che; Drosophila melanogaster, Dme; Ephydatia muelleri, Emu; Hydra magnipapillata, Hma; Homo sapiens, HSA; Nematostella vectensis, Nve; Pleurobrachia pileus, Ppi. [file 2041-9139-2-12-S1.DOC]

Additional file 1

**10 20 30 40 50 60 70 80 90**

**CinSoxB1**  **----------** **----------** **----------** **----------** **----------** **----------** **----------** **----------** **----------**

**DmeSoxN**  **----------** **-----LTMES** **DMKGSLLHAT** **MPPHHTSAAL** **HGHAASPYSA** **LAPLMNLGQS** **HLTHSQLSHH** **NHHHHHMSAH** **IAASQSPNPL**

**HSASox1**  **----------** **----------** **----------** **----------** **----------** **----------** **----------** **----------** **----------**

**CelSoxB1**  **----------** **----------** **----------** **----------** **----------** **----------** **----------** **----------** **----------**

**HSASox3**  **----------** **----------** **----------** **----------** **--MRPVRENS** **SGARSPRVPA** **DLARSILISL** **PFPPDSLAHR** **PPSSAPTESQ**

**HSASry**  **----------** **----------** **----------** **----------** **----------** **----------** **----------** **----------** **----------**

**PpiSox3**  **----------** **----------** **----------** **----------** **----------** **----------** **----------** **----------** **----------**

**HSASox15**  **----------** **----------** **----------** **----------** **----------** **----------** **----------** **----------** **----------**

**HSASox2**  **----------** **----------** **----------** **----------** **----------** **----------** **----------** **----------** **----------**

**HSASox14**  **----------** **----------** **----------** **----------** **----------** **----------** **----------** **----------** **----------**

**DmeSox21b**  **MMERLSTHLS** **LTGLSLAHPL** **QTHLGSGGGT** **HHLGQLAAAV** **HQQQQQQQQS** **DQGLGHSHHL** **HGQQLSLNHQ** **HHHPHQSPQH** **QQQHQHHPCS**

**DmeSoxdich** **----------** **----------** **----------** **----------** **---------M** **ATLSTHPNYG** **FHLGQAQGLE** **DYAPQSQLQL** **SPGMDMDIKR**

**DmeSox21a**  **----------** **----------** **----------** **----------** **----------** **----------** **MTSISALLHR** **SHSHSNGYTS** **SGSSNHSHSS**

**HSASox21**  **----------** **----------** **----------** **----------** **----------** **----------** **----------** **----------** **----------**

**CinSoxB2**  **----------** **----------** **----------** **----------** **----------** **----------** **----------** **----------** **----------**

**CelSoxB2**  **----------** **----------** **----------** **----------** **----------** **----------** **----------** **----------** **----------**

**AmiSoxB1**  **----------** **----------** **----------** **----------** **----------** **----------** **----------** **----------** **----------**

**NveSoxB1**  **----------** **----------** **----------** **----------** **----------** **----------** **----------** **----------** **----------**

**AmiSoxBa**  **----------** **----------** **----------** **----------** **----------** **----------** **----------** **----------** **----------**

**NveSoxB2**  **----------** **----------** **----------** **----------** **----------** **----------** **----------** **----------** **----------**

**HmaSox10**  **----------** **----------** **----------** **----------** **----------** **----------** **----------** **----------** **----------**

**CheSox10**  **----------** **----------** **----------** **----------** **----------** **----------** **----------** **----------** **----------**

**CheSox3**  **----------** **----------** **----------** **----------** **----------** **----------** **----------** **----------** **----------**

**HmaSoxB1**  **----------** **----------** **----------** **----------** **----------** **----------** **----------** **----------** **----------**

**NveSox1**  **----------** **----------** **----------** **----------** **----------** **----------** **----------** **----------** **----------**

**CheSox2**  **----------** **----------** **----------** **----------** **----------** **----------** **----------** **----------** **----------**

**HmaSox3**  **----------** **----------** **----------** **----------** **----------** **----------** **----------** **----------** **----------**

**NveSox2**  **----------** **----------** **----------** **----------** **----------** **----------** **----------** **----------** **----------**

**AquSOXB1**  **----------** **----------** **----------** **----------** **----------** **----------** **----------** **----------** **----------**

**EmuSox2**  **----------** **----------** **----------** **----------** **----------** **----------** **----------** **----------** **----------**

**AquSoxB2**  **----------** **----------** **----------** **----------** **----------** **----------** **----------** **----------** **----------**

**CheSox13**  **----------** **----------** **----------** **----------** **----------** **----------** **---------H** **SNSKEPKTFT** **KMPIMYEKVI**

**HmaSox1**  **----------** **----------** **----------** **----------** **----------** **----------** **----------** **----------** **----------**

**CheSox14**  **----------** **----------** **----------** **----------** **----------** **----------** **----------** **----------** **----------**

**HmaSoxBb**  **----------** **----------** **----------** **----------** **----------** **----------** **----------** **----------** **----------**

**NveSox3**  **----------** **----------** **----------** **----------** **----------** **----------** **----------** **----------** **----------**

**AmiSoxBb**  **----------** **----------** **----------** **----------** **----------** **----------** **----------** **----------** **----------**

**100 110 120 130 140 150 160 170 180**

**CinSoxB1**  **----------** **----------** **----------** **----------** **----------** **------MTHL** **SPPMMNHVQG** **SHNQNNNNSS** **ANNK------**

**DmeSoxN**  **SSLQSSMANT** **LNGSQVGQQQ** **QQQQQQQSSP** **LHSSSELSPT** **QSSIGSHHMT** **SPVSHQQHTQ** **QQQHGQQQHL** **GAGSALSSLT** **GGSSNNNNNS**

**HSASox1**  **----------** **----------** **----------** **----------** **-MYSMMMETD** **LHSPGGAQAP** **TNLSGPAGAG** **GGGGGGGGGG** **GGGGA-----**

**CelSoxB1**  **----------** **----------** **----------** **-MMMDPDSAK** **MQDYSGWSFG** **FHMPPTSSTN** **LLPQSMPDDL** **SNGPDSPDST** **GKDG------**

**HSASox3**  **GLFTVAAPAP** **GAPSPPATLA** **HLLPAPAMYS** **LLETELKNPV** **GTPTQAAGTG** **GPAAPGGAGK** **SSANAAGGAN** **SGGGSSGGAS** **GGGG------**

**HSASry**  **----------** **----------** **----------** **-MQSYASAML** **SVFNSDDYSP** **AVQENIPALR** **RSSSFLCTES** **CNSKYQCETG** **ENSK------**

**PpiSox3**  **----------** **----------** **----------** **----------** **----------** **----------** **----------** **----------** **----------**

**HSASox15**  **----------** **----------** **----------** **----------** **--MALPGSSQ** **DQAWSLEPPA** **ATAAASSSSG** **PQEREGAGSP** **AAPG------**

**HSASox2**  **----------** **----------** **----------** **----------** **------HSAR** **MYNMMETELK** **PPGPQQTSGG** **GGGNSTAAAA** **GGNQ------**

**HSASox14**  **----------** **----------** **----------** **----------** **----------** **----------** **----------** **----------** **---M------**

**DmeSox21b**  **SNSSNGSPNG** **STGLGLHPHS** **ALHLAHHHSQ** **LHQHHPAGQQ** **QQHSQPPVSS** **SSTSHHSQSQ** **ALSHQTHSNG** **SSQLGGGSAS** **GAGSVAGGAA**

**DmeSoxdich** **VLHYSQSLAA** **MGGSPNGPAG** **QGVNGSSGMG** **HHMSSHMTPH** **HMHQAVSAQQ** **TLSPNSSIGS** **AGSLGSQSSL** **GSNGSGLNSS** **SGHQSAGMHS**

**DmeSox21a**  **SLSPQLPGIN** **LGLGMVGGLG** **MGMSVGVGSG** **SGNTTPPPMP** **PADLAVPPAA** **PTPVAPKMHQ** **HTHHHGNSHH** **NAPTSHSNSN** **TGSH------**

**HSASox21**  **----------** **----------** **----------** **----------** **----------** **----------** **----------** **----------** **---M------**

**CinSoxB2**  **----------** **----------** **----------** **----------** **----------** **----------** **----------** **---------M** **TLKS------**

**CelSoxB2**  **----------** **----------** **----------** **----------** **---MTDLSCL** **YPSLLCTEAA** **KTSYDEDTTS** **VSSGLSPPGS** **PVDL------**

**AmiSoxB1**  **----------** **----------** **----------** **----------** **----------** **--------MS** **TTTILTSSPN** **AGSNSNAESN** **G---------**

**NveSoxB1**  **----------** **----------** **----------** **----------** **----------** **--------MS** **ATTILTPSPN** **PISGNGTNGN** **ESTK------**

**AmiSoxBa**  **----------** **----------** **----------** **----------** **----------** **----------** **----------** **----------** **--MG------**

**NveSoxB2**  **----------** **----------** **----------** **----------** **----------** **----------** **----------** **----------** **--MG------**

**HmaSox10**  **----------** **----------** **----------** **----------** **---MNAMVTS** **QAKPEFMTPH** **TTQLGLHQSQ** **SPAPTVAPTS** **PNPL------**

**CheSox10**  **----------** **----------** **----------** **--MYNTMNTM** **VVQAKPEMMT** **HIPHPHHQLK** **PGVGGPGSPA** **MQHPGLVAHP** **GLVG------**

**CheSox3**  **----------** **----------** **----------** **----------** **---------M** **VTTTYDPNVH** **HAAIDISNSM** **HAFATELSVD** **PTKA------**

**HmaSoxB1**  **----------** **----------** **--------MA** **TAINVIQHPS** **DANNDTHFKQ** **VVGYNTSQSM** **LVPQNLNLSN** **RVSNSLVSDH** **ETSK------**

**NveSox1**  **----------** **----------** **----------** **----------** **----------** **----------** **----------** **----------** **-MSL------**

**CheSox2**  **----------** **----------** **----------** **----------** **----------** **----------** **----------** **---MEMSNLE** **PAGH------**

**HmaSox3**  **----------** **----------** **----------** **----------** **----------** **----------** **CNYFFSKTTK** **ICTYKNFVKM** **ASDL------**

**NveSox2**  **----------** **----------** **----------** **----------** **----------** **----------** **----------** **----------** **----------**

**AquSOXB1**  **----------** **----------** **MLERESTMHI** **MSLPNMPGYP** **PHYAMPGPTG** **STSIVPPQAS** **ELEHSVSPPL** **VGGQTGNGGP** **HGSNPSD---**

**EmuSox2**  **----------** **----------** **----------** **----------** **----------** **----------** **----------** **----------** **----------**

**AquSoxB2**  **----------** **----------** **----------** **----------** **----------** **----------** **----------** **----------** **----------**

**CheSox13**  **PKIEPLELGG** **NMTSFHGHPG** **NAQMMTAHHG** **TPMHILPSHQ** **MIGLHSHHHQ** **NMSPGPQMNG** **MQHHDENSPI** **SMTTVQTASP** **VTTNQSESD-**

**HmaSox1**  **----------** **----------** **------MYEK** **SIKCEPIEML** **HSYNHGVMQQ** **LHSNNNSMSP** **NEQINSPSPH** **QSGVQINSVT** **NISE------**

**CheSox14**  **----------** **----------** **----MLSTSA** **MSQPEDHGPT** **AGQMVSAVNV** **NSSLSNGQTN** **GQFNDPISHH** **PGHDHPVDHK** **DEKK------**

**HmaSoxBb**  **----------** **----------** **----------** **MSSMQHKQLA** **NEHVTSQSAS** **AINQTSALQQ** **NGHYENRTQV** **TLESNDIKSE** **PDNKIK----**

**NveSox3**  **----------** **----------** **----------** **----------** **----------** **----------** **----------** **----------** **--MS------**

**AmiSoxBb**  **----------** **----------** **----------** **----------** **----------** **----------** **----------** **----------** **--MS------**

**190 200 210 220 230 240 250 260 270**

**CinSoxB1**  **----------** **----------** **----------** **----------** **----------** **----------** **-VPQEQRVKR** **PMNAFMVWSR** **GQRRKMAQDN**

**DmeSoxN**  **ATANKN----** **----------** **----------** **----------** **----------** **----------** **-QQHADRVKR** **PMNAFMVWSR** **GQRRKMASDN**

**HSASox1**  **----------** **----------** **----------** **----------** **----------** **----------** **-KANQDRVKR** **PMNAFMVWSR** **GQRRKMAQEN**

**CelSoxB1**  **----------** **----------** **----------** **----------** **----------** **----------** **-KKNDDRVKR** **PMNAFMVWSR** **GQRKKMALEN**

**HSASox3**  **----------** **----------** **----------** **----------** **----------** **----------** **-GTDQDRVKR** **PMNAFMVWSR** **GQRRKMALEN**

**HSASry**  **----------** **----------** **----------** **----------** **----------** **----------** **-GNVQDRVKR** **PMNAFIVWSR** **DQRRKMALEN**

**PpiSox3**  **----------** **----------** **----------** **----------** **----------** **----------** **----------** **------VWSR** **GQRRKMAQEN**

**HSASox15**  **----------** **----------** **----------** **----------** **----------** **----------** **-TLPLEKVKR** **PMNAFMVWSS** **AQRRQMAQQN**

**HSASox2**  **----------** **----------** **----------** **----------** **----------** **----------** **-KNSPDRVKR** **PMNAFMVWSR** **GQRRKMAQEN**

**HSASox14**  **----------** **----------** **----------** **----------** **----------** **----------** **-SKPSDHIKR** **PMNAFMVWSR** **GQRRKMAQEN**

**DmeSox21b**  **NSHHSAPASS** **SVMAAAAAAH** **LHHNSQASPI** **SNLHQNMGSL** **MNSGSSASDV** **FFSLMIQNTT** **KRQNEEHIKR** **PMNAFMVWSR** **LQRRKIAQDN**

**DmeSoxdich** **LATS------** **----------** **----------** **----------** **----------** **----------** **-PGQEGHIKR** **PMNAFMVWSR** **LQRRQIAKDN**

**DmeSox21a**  **----------** **----------** **----------** **----------** **----------** **----------** **-HNSHDHIKR** **PMNAFMVWSR** **GQRRKMAQDN**

**HSASox21**  **----------** **----------** **----------** **----------** **----------** **----------** **-SKPVDHVKR** **PMNAFMVWSR** **AQRRKMAQEN**

**CinSoxB2**  **----------** **----------** **----------** **----------** **----------** **----------** **-GIDTGHVKR** **PMNAFMVWSR** **GQRRKMAQEN**

**CelSoxB2**  **----------** **----------** **----------** **----------** **----------** **----------** **-QNSLDHVKR** **PMNAFMVWSR** **GQRRKMAQDN**

**AmiSoxB1**  **----------** **----------** **----------** **----------** **----------** **----------** **-KCSPDRVKR** **PMNAFMVWSR** **ERRRRMAQEN**

**NveSoxB1**  **----------** **----------** **----------** **----------** **----------** **----------** **-ANSADRVKR** **PMNAFMVWSR** **ERRRKMAQDN**

**AmiSoxBa**  **----------** **----------** **----------** **----------** **----------** **----------** **-KQEEGHIKR** **PMNAFMVWSR** **GKRKQYAAIN**

**NveSoxB2**  **----------** **----------** **----------** **----------** **----------** **----------** **-KQEDGHVKR** **PMNAFMVWSR** **GKRKHYASIN**

**HmaSox10**  **----------** **----------** **----------** **----------** **----------** **----------** **-NGDMSHVKR** **PMNAFMVWSR** **GKRRQMAQDN**

**CheSox10**  **----------** **----------** **----------** **----------** **----------** **----------** **-LNGDNHVKR** **PMNAFMVWSR** **GKRRQMAQEH**

**CheSox3**  **----------** **----------** **----------** **----------** **----------** **----------** **-GDDPDRIKR** **PMNSFMVWSR** **EKRRKLAQEN**

**HmaSoxB1**  **----------** **----------** **----------** **----------** **----------** **----------** **-QEDFDKVKR** **PMNSFMVWSR** **EKRRRLAHEN**

**NveSox1**  **----------** **----------** **----------** **----------** **----------** **----------** **-VKPVEHVKR** **PMNAFMVWSR** **EERRKIAQEN**

**CheSox2**  **----------** **----------** **----------** **----------** **----------** **----------** **-HAKQNHIKR** **PMNSFMVWSR** **MERKRISEEN**

**HmaSox3**  **----------** **----------** **----------** **----------** **----------** **----------** **-DKPKGHIKR** **PMNSFMVWSR** **MERKRISEAN**

**NveSox2**  **----------** **----------** **----------** **----------** **----------** **----------** **MTKPGDHIKR** **PMNAYMVWSR** **KERRRIAEEC**

**AquSOXB1**  **----------** **----------** **----------** **----------** **----------** **----------** **-MEEDDKVKR** **PMNAFMVWSR** **KMRKKIADEN**

**EmuSox2**  **----------** **----------** **----------** **----------** **----------** **----------** **----------** **------VWSR** **KMRKKIADEN**

**AquSoxB2**  **----------** **----------** **----------** **----------** **----------** **----------** **-----DHIKR** **PMNAFMVWSK** **ERRKELAQEN**

**CheSox13**  **----------** **----------** **----------** **----------** **----------** **----------** **-KKARDHVKR** **PMNAFMVWSR** **EKRKKMSQIN**

**HmaSox1**  **----------** **----------** **----------** **----------** **----------** **----------** **-QKKPDHVKR** **PMNAFMVWSR** **EKRRKMAQIN**

**CheSox14**  **----------** **----------** **----------** **----------** **----------** **----------** **-SDEPPKIKR** **PMNPFMIFGC** **EKRRKLAQVH**

**HmaSoxBb**  **----------** **----------** **----------** **----------** **----------** **----------** **-NDESTKIKR** **PMNPFMIFGC** **EKRRKLAQVH**

**NveSox3**  **----------** **----------** **----------** **----------** **----------** **----------** **-TKDANHVKR** **PMNAFMVWSK** **ERRRIKSQEC**

**AmiSoxBb**  **----------** **----------** **----------** **----------** **----------** **----------** **-GKDPDHIKR** **PMNAFMVWSK** **EKRRTMSQKN**

**HMG domain**

*****

**280 290 300 310 320 330 340 350 360**

**CinSoxB1**  **PKMHNSEISK** **RLGAEWKTLS** **DSDKRPFIDE** **AKRLRALHMK** **EHPDYKYRP-** **RRKTKAILKK** **ENK-------** **FGMGPGGIMG** **GGAGPQMSAS**

**DmeSoxN**  **PKMHNSEISK** **RLGAQWKDLS** **ESEKRPFIDE** **AKRLRAVHMK** **EHPDYKYRP-** **RRKTKTLTKT** **KEK-------** **YPMGGLMPGQ** **TVGGGAPGEP**

**HSASox1**  **PKMHNSEISK** **RLGAEWKVMS** **EAEKRPFIDE** **AKRLRALHMK** **EHPDYKYRP-** **RRKTKTLLKK** **-DK-------** **YSLAGGLLAA** **GAGGGGAAVA**

**CelSoxB1**  **PKMHNSEISK** **RLGTEWKMLS** **EQEKRPFIDE** **AKRLRAIHMK** **EHPDYKYRP-** **RRKTKSINKK** **NGA-------** **----------** **----------**

**HSASox3**  **PKMHNSEISK** **RLGADWKLLT** **DAEKRPFIDE** **AKRLRAVHMK** **EYPDYKYRP-** **RRKTKTLLKK** **-DK-------** **YSLPSGLLPP** **GAAAAAAAA-**

**HSASry**  **PRMRNSEISK** **QLGYQWKMLT** **EAEKWPFFQE** **AQKLQAMHRE** **KYPNYKYRP-** **RRKAKMLPKN** **CSL-------** **----------** **----------**

**PpiSox3**  **PKMHNSEISK** **RLGLDWKMLT** **EDEKKPFIEE** **AKRLRALHMK** **EFPDYKYRP-** **RRKAKAALKK** **-DR-------** **----------** **----------**

**HSASox15**  **PKMHNSEISK** **RLGAQWKLLD** **EDEKRPFVEE** **AKRLRARHLR** **DYPDYKYRP-** **RRKAKSSGAG** **PSR-------** **----------** **----------**

**HSASox2**  **PKMHNSEISK** **RLGAEWKLLS** **ETEKRPFIDE** **AKRLRALHMK** **EHPDYKYRP-** **RRKTKTLMKK** **-DK-------** **YTLPGGLLAP** **GGNSMA----**

**HSASox14**  **PKMHNSEISK** **RLGAEWKLLS** **EAEKRPYIDE** **AKRLRAQHMK** **EHPDYKYRP-** **RRKPKNLLKK** **-DR-------** **YVFP----LP** **YL--------**

**DmeSox21b**  **PKMHNSEISK** **RLGAEWKLLT** **EEEKRPFIDE** **AKRLRAMHMK** **EHPDYKYRP-** **RRKPKAL-RR** **-DG-------** **--YPYPMPYP** **SVPVEALRAG**

**DmeSoxdich** **PKMHNSEISK** **RLGAEWKLLA** **ESEKRPFIDE** **AKRLRALHMK** **EHPDYKYRP-** **RRKPKNPLTA** **GPQ-------** **----------** **-GGLQM----**

**DmeSox21a**  **PKMHNSEISK** **RLGAEWKLLT** **EGQKRPFIDE** **AKRLRALHMK** **EHPDYKYRP-** **RRKPKTLNKS** **PVP-------** **----------** **GGGGGG----**

**HSASox21**  **PKMHNSEISK** **RLGAEWKLLT** **ESEKRPFIDE** **AKRLRAMHMK** **EHPDYKYRP-** **RRKPKTLLKK** **-DK-------** **FAFP----VP** **YGLGGVADA-**

**CinSoxB2**  **PKMHNSEISK** **RLGASWKLLN** **ECEKRPFIDE** **AKRLRALHMK** **EHPDYKYRP-** **RRKPKSLIKP** **KDR-------** **FAFPLFPSSG** **MSSP------**

**CelSoxB2**  **PKMHNSEISK** **RLGAEWKQLS** **EQEKRPFIDE** **AKRLRALHMK** **EHPDYKYRP-** **RRKPKSSNLK** **QQP-------** **----------** **----------**

**AmiSoxB1**  **PKMHNSEISK** **RLGAEWKQLS** **DPEKRPYVDE** **AKRLRAVHMK** **DHPDYKYRP-** **RRKSKTLLKK** **-DN------K** **YTLS-MLGAQ** **GGPPVQRSM-**

**NveSoxB1**  **PKMHNSEISK** **RLGSEWKLLS** **EQEKRPYIDE** **ARRLRAVHMK** **EHPDYKYRP-** **RRKSKTLLKK** **-DN------K** **YALSMLSAGQ** **AGGQVQRTV-**

**AmiSoxBa**  **PRMHNSEISK** **RLGAEWKMLS** **QDEKEPFVAE** **AKRLQAIHIQ** **EHPDYKYKPK** **RRKPKSLQKK** **-EL-------** **---SGPMFSP** **YSSPMMAV--**

**NveSoxB2**  **PRMHNSEISK** **RLGAEWKMLT** **AEEKEPFIAE** **AKRLQALHIQ** **EHPDYKYKPK** **RRKPKSVQKK** **-DL-------** **---ASPVFSP** **YAASMMAVDK**

**HmaSox10**  **PRMHNSEISK** **RLGAEWKCLT** **QQEKQPFIDE** **AKRLRAVHIQ** **EHPDYKYKPK** **RRKQKTT-KK** **-DI-------** **-----YTPYP** **NIGQGMVP--**

**CheSox10**  **PRMHNSEISK** **RLGAQWKVLT** **PEEKQPFIDE** **AKRLRAVHIQ** **EHPDYKYKPK** **RRKPKQM-KK** **-DM-------** **--------YP** **AYSNMAASTT**

**CheSox3**  **PKMHNSEISK** **RLGAEWKVLT** **EEEKAPFVYE** **AKRLRAEHMK** **THPDYKYRP-** **RRKNKNAPKK** **TEH------K** **ITMPTAVIGA** **DGKQIFMPAQ**

**HmaSoxB1**  **PKMHNSEISK** **RLGAEWKVLT** **EDEKAPFVFE** **AKRLRAEHMK** **SHPDYKYRP-** **RRKAKTSSKK** **NEQ-------** **-KMPHVITTD** **GKQIF-----**

**NveSox1**  **PKMHNSEISK** **RLGSEWKQLA** **DDDKKPFVEE** **AKKLRAQHMK** **EHPDYKYRP-** **RRMPKSLLKK** **TDR-------** **YPFPLPC---** **----------**

**CheSox2**  **PKLHNSEISK** **RLGASWKMLS** **EEERKPFAEE** **AKRLRQIHIQ** **EHPEYKYRP-** **RRKPKLSVPT** **QHH-------** **----------** **----------**

**HmaSox3**  **PKMHNSEISK** **QLGTSWKMLS** **EEDRAPYAEE** **AKRLRDLHMS** **EYPDYKYRP-** **KRKPKASSVN** **QEK-------** **----------** **----------**

**NveSox2**  **PRMLNSEISK** **RLGLEWNSLT** **LDEKQPYVEE** **AKRLRELHKK** **DHPDYKYQP-** **KRKPKTSPKL** **KTP-------** **----------** **----------**

**AquSOXB1**  **PKMHNSEISK** **RLGTQWKALS** **EEDKRPFIDE** **AKRLREAHMK** **KHPNYKYKPK** **RKKQTPTNTR** **IIPGIGSWPP** **YHQSRHIVHA** **GHIPSGGRWQ**

**EmuSox2**  **PKMHNSEISK** **RLGAQWKALS** **DEEKRPYIEE** **AKRLREAHMK** **KHPNYKYKPK** **RKKQQPLRRF** **PMD------M** **AASPYGPYFP** **QRPTGLAXPL**

**AquSoxB2**  **PRMHNSELSK** **KLGAEWKALS** **DTNKHRYIEE** **AKKIREQHMA** **EFPHYRYRP-** **RRKPKNPFKA** **GGG-----GG** **SGRMSVASTP** **YSLSSLSPGS**

**CheSox13**  **PRMHNSEISK** **ILGAEWKRMT** **EQEKGPYIEE** **AKRLQTQHSI** **EYPNYKYKPR** **RRKPKAMMKK** **-DK-------** **------MGYP** **YDA-------**

**HmaSox1**  **PRMHNSEISK** **ILGSEWKRMG** **ESEKGPYVLE** **AKRLQTQHSI** **EYPNYKYKPR** **RRKAKAMLKK** **-DK-------** **----IGFVYP** **GDV-------**

**CheSox14**  **PRMHNSEISK** **ILGAEWKRMS** **DYEKAPYIQE** **AKRLKEQHSI** **EYPNYKFKAN** **RRKPRQAVKK** **-ER-------** **------PAFP** **YATDL-----**

**HmaSoxBb**  **PRMHNSEISK** **ILGAEWKRMS** **DYEKSPYIQE** **AKRLKEQHSI** **EYPNYKFKAN** **RRKPRQAIKK** **-ER-------** **------PSFP** **YAADI-----**

**NveSox3**  **PRMHNSEISK** **ILGCEWKATK** **DELKQPYIEK** **AKELQAQHSR** **ENPGYKYKPR** **RRKPKQTLLK** **KAA-------** **------YPFP** **YTST------**

**AmiSoxBb**  **PKMHNSEISK** **ILGAQWKKMP** **DEEKAKYIEE** **AKRLQQEHSQ** **KHPDYKYKPR** **RRKQKQLI-K** **KAT-------** **------YSFP** **YTG-------**

**Group B motif**

**HMG domain**

**370 380 390 400 410 420 430 440 450**

**CinSoxB1**  **GQRSHM-SAA** **PIDYSQ-YYQ** **HSMMAG---S** **QDPMAYGAPS** **PHA------Y** **PAMANSGTAA** **N----RYDMY** **YPSYSTPTTL** **PSMSSL----**

**DmeSoxN**  **VTPTRVQGQP** **GQNQSLNGSG** **GSAAAAAAAA** **AAAAQQARQD** **MYQMNAPNGY** **MPNGYMMHAD** **PAGAAAYQTS** **YMGQHYAAQR** **YDMGHMYNNG**

**HSASox1**  **MGVGVGVGAA** **PVGQRL-ESP** **GGAAGG---A** **YAHVNGWANG** **AYPGSVAAAA** **AAAAMMQEAQ** **L----AYGQH** **PGAGGAHPHR** **TPAHPHPHHP**

**CelSoxB1**  **---------P** **IPFGNL-DTK** **TPSYPTLTTN** **WNATNQYIDQ** **F---------** **----------** **-----RFAPY** **PTTTVMDQIP** **FSL-------**

**HSASox3**  **-------AAA** **AAAASS-PVG** **VGQRLD---T** **YTHVNGWANG** **AYS-------** **-----LVQEQ** **L----GYAQP** **PSMSSPPP--** **----------**

**HSASry**  **---------L** **PAD-------** **----------** **----------** **----------** **----------** **-----PASVL** **CSEVQL----** **----------**

**PpiSox3**  **----------** **------YGVP** **CGTDRG---L** **LMDRSSYIQS** **SIP-------** **----------** **-----TNSGY** **IPTSTYKAMM** **DAYQ------**

**HSASox15**  **----------** **---CGQ-GRG** **NLASGG--PL** **WGPGYATTQP** **SR--------** **----------** **-----GFGYR** **PPSYST----** **----------**

**HSASox2**  **-------SGV** **GVGAGL-GAG** **VNQRMD---S** **YAHMNGWSNG** **SYS-------** **-----MMQDQ** **L----GYPQH** **PGLNAHGAAQ** **----------**

**HSASox14**  **-------GDT** **DPLKAA-GLP** **VGASDGLLSA** **PEKARAFLPP** **ASA-------** **----------** **-----PYSLL** **DPAQFSSSAI** **QKM-------**

**DmeSox21b**  **ITPG---YFA** **PGPTAA-AYH** **LGSHLGQTST** **PTTTQATLSG** **QMDKFALERS** **SYLSNSSQAS** **-----AYSAY** **LDPSVLTKAY** **FDSKMYQDRA**

**DmeSoxdich** **-------QAG** **MGQQKL-GAG** **PGAGAGGYNP** **FHQLPPYFAP** **SHHLDQ----** **----------** **-----GYPVP** **YFGGFDPLA-** **----------**

**DmeSox21a**  **-------GGG** **GANGGV-NAG** **GAGNSG---P** **SGPGSVGSPK** **DMQ-------** **-------PQL** **S----PLGQS** **LPHLHGHPHQ** **SPYQSH----**

**HSASox21**  **-------EHP** **ALKAGA-GLH** **AGAGGG---L** **VPESLLANPE** **KAA-------** **-----AAAAA** **A----AARVF** **FPQSAAAAAA** **AAAAAA----**

**CinSoxB2**  **-------THD** **ASTVSL-AIQ** **QAAAAAHLNM** **DPIVVEKARA** **A---------** **----------** **-----AAAAF** **YSTSQSLTTT** **SFPSLN----**

**CelSoxB2**  **----------** **--RLNI-AMP** **TIPPQS---L** **FNYSTAFDSL** **KTH-------** **----------** **-----DLSQY** **YSSFFQSPVL** **SGS-------**

**AmiSoxB1**  **-------VQN** **PADHFG-QMN** **GFAYSP---I** **TGYNQMNVND** **PYSN------** **----------** **-----IYAGH** **PLSPHTPTQI** **QPSNGLHHTA**

**NveSoxB1**  **-------GQN** **PGDHFVQMAG** **YGYSQG----** **---LSYNPND** **PYGAMYGSNA** **MSPHTPTQLQ** **P----PMNGL** **PPHGSYSNMS** **ASAQIYPS--**

**AmiSoxBa**  **-------DKF** **PTNQLPQTIA** **HSTALSADPM** **YSKINGAAAA** **FHHSVSP---** **----------** **-----GYPVI** **YPNVTAVNSH** **HSVT------**

**NveSoxB2**  **FST----NQL** **PQTIAH-SVA** **LSQDP----M** **YSKINGGPPF** **HHS-------** **--------VS** **P----GYPVI** **YPSVSNGGNV** **HSGS------**

**HmaSox10**  **-------NID** **SKYASI-GYQ** **PTLSYG---M** **SSDMYNKLNG** **GYG-------** **----YQTTIS** **T----GYPLM** **YSNYSVGPSM** **VGS-------**

**CheSox10**  **IIP----GMD** **PKYGGM-AYQ** **QSMAYGISTM** **SPDLYGKMNA** **AYA-------** **----YPAAIS** **P----GYPVM** **YSNYPMTTMG** **TTAPSAGSGS**

**CheSox3**  **-------YAA** **APGYAI-ASA** **GGMNYPVPLN** **AAYMSALVNG** **EAA-------** **-----AYPAG** **T----AMYGI** **PPGTAIAVAA** **TTVAAQTTQT**

**HmaSoxB1**  **-------MPT** **QYTSGY-AIT** **GGINYP-FNG** **YLSALVNGHE** **AYA-------** **-----SNTSP** **S----AVYGF** **PHGATIAVTS** **TISQ------**

**NveSox1**  **-------IPT** **PDELSK-CV-** **---------S** **LSSTSSLMSD** **SYS-------** **----------** **-----KARSY** **MQVSS-----** **----------**

**CheSox2**  **---------V** **RKHQST-AAG** **KTVPIPIPEY** **IPSRSGSRPA** **----------** **----------** **-----VVPVY** **YHSPHY----** **----------**

**HmaSox3**  **---------L** **PS--------** **----------** **----------** **----------** **----------** **----------** **----------** **----------**

**NveSox2**  **---------G** **LNPFMH-GYG** **EMPGIG---M** **PPPTNLCQPM** **ASH-------** **----------** **-----MGPMV** **NMASCPGSCT** **LPE-------**

**AquSOXB1**  **YPQESGYYYT** **PGNGQH-SYY** **TGYSGG----** **YTRSPTVPSP** **SYHSWNNMTA** **PYPNTGQQHS** **P----LPTDY** **ATGSSVPVTM** **GPGACAQQST**

**EmuSox2**  **QAFMQADRYG** **RDKLHS-TRS** **SEXQCVRKPI** **LLRFVSTRPV** **LLRRRLLLLE** **PDQRSLCGQS** **WKLHLGHRAV** **GXLTVTIAVT** **VTGCKTVTST**

**AquSoxB2**  **TGSTSSNCSS** **STNTAS-DAA** **GFVGPHQVQI** **LPQQVVTSHG** **LHHSPIATTT** **NFIQSLQPAA** **SIA--GGGTT** **YLIQRQPLLP** **AGTQIIQTAT**

**CheSox13**  **-------NGV** **PAGMKF-PY-** **---------P** **YANIATFPQD** **A---------** **----------** **-----MYTPI** **YQMPGAPA--** **----------**

**HmaSox1**  **-------NGI** **PANMKF-PYP** **TSAYSQDAMY** **GPPIYQIPNA** **P---------** **----------** **-----GYAMY** **ADYSQSIH--** **----------**

**CheSox14**  **-------TAL** **PTGMKY-PYP** **SPFFQE--SI** **YGSMFPGMPS** **GSHP------** **----------** **-----AYTTM** **YSD-------** **----------**

**HmaSoxBb**  **-------NTI** **PPAMKF-PYP** **SPFFQD--SM** **YGTMYPVMGN** **SNHP------** **----------** **-----AYSSM** **YSDFARQSMS** **STGVLRTG--**

**NveSox3**  **-------EMA** **PXAMKM-GYA** **GGMPTP-ESM** **YQQFYPMQGQ** **P---------** **----------** **-----AYSVY** **DMSGVHAARQ** **TYAG------**

**AmiSoxBb**  **-------TEN** **AAHAAA-AMK** **LSAYPP---S** **MAPDSMHYQQ** **YYQ-------** **-----MSQHA** **P-----YPTM** **YDMAAVHAQR** **QTHSFS----**

**460 470 480 490 500 510 520 530 540**

**CinSoxB1**  **------TSQH** **NSYAQSAYSV** **GGSPAYSVAQ** **AH------TP** **TNSLHSMAGS** **HHGSSGVHSP** **ANTSPGA---** **----------** **----------**

**DmeSoxN**  **YAMYQTVSGG** **QTSPYGSSLQ** **QPGSPSPYGG** **SSLQQQPGSP** **TPYGGGGGGG** **GQVSCQSHSP** **SDSSIKS---** **----------** **------EPVS**

**HSASox1**  **HAHPHNPQPM** **HRYDMGALQY** **SPISNSQGYM** **SA------SP** **SGYGGLPYGA** **AAAAAAAHQN** **SAVAAAA---** **----------** **----------**

**CelSoxB1**  **------TYPV** **HSVPTDNSSP** **SQFQPSPMST** **----------** **-----NFAGS** **YLTPKSESSP** **VGSDSTV---** **----------** **----------**

**HSASox3**  **------PPAL** **HRYDMAGLQY** **SPMMPPGAQS** **--------YM** **NVAAAAAAAS** **GYGGMAPSAT** **AAAAAAY---** **----------** **----------**

**HSASry**  **------DNRL** **YRDDCTKATH** **SRMEHQLGHL** **----------** **----------** **----------** **----------** **----------** **----------**

**PpiSox3**  **------SQVS** **TLHRSASAVQ** **YGTTAAELGT** **----------** **-RYPEFVSNP** **YHGYMSPVNP** **CTSPYGS---** **----------** **----------**

**HSASox15**  **------AYLP** **GSYGSSHCKL** **EAPSPCSLPQ** **SD--------** **----PRLQGE** **LLPTYTHYLP** **PGSPTPY---** **----------** **----------**

**HSASox2**  **------MQPM** **HRYDVSALQY** **NSMTSSQTYM** **NG------SP** **TYSMSYSQQG** **TPGMALGSMG** **SVVKSEA---** **----------** **----------**

**HSASox14**  **------GEVP** **HTLATGALPY** **ASTLGYQNGA** **----------** **-----FGSLS** **CPSQHTHTHP** **SPTNPGY---** **----------** **----------**

**DmeSox21b**  **ANYAFDISKI** **YGAQQHAAAH** **HQQQQQQQQQ** **QQ--------** **----QQQQQL** **LLSGGGGSGG** **GGSASSH---** **----------** **----------**

**DmeSoxdich** **------LSKL** **HQSQAAAAAA** **VNNQGQQQGQ** **--------AP** **PQLPPTSLSS** **FYSGIYSGIS** **APSLYAA---** **----------** **----------**

**DmeSox21a**  **------PHHP** **HPHPHHVQLA** **AATLSAKYGF** **G-------SP** **LELSLPRLPN** **AFPGLAHYPL** **DPTLALD---** **----------** **----------**

**HSASox21**  **------AGSP** **YSLLDLGSKM** **AEISSSSSGL** **PY-----ASS** **LGYPTAGAGA** **FHGAAAAAAA** **AAAAAGG---** **----------** **----------**

**CinSoxB2**  **------PLSM** **TSQSNAVSLA** **AAITQSRLAA** **VAAARDLGTP** **NSMYSPSNGL** **YSPPATTGAN** **KIKSVLY---** **----------** **----------**

**CelSoxB2**  **------TYAP** **YNMMAAYARQ** **AAAVAAASQV** **----------** **----------** **------SAST** **TPTAPAT---** **----------** **----------**

**AmiSoxB1**  **YSHMGGSGQI** **YSTVSTQGQV** **YALNGTQSMN** **SI------SP** **LSYSQPSNQV** **LIPNIKQEMP** **SPGSQSS---** **----------** **----------**

**NveSoxB1**  **------VSVM** **TSQPVGAASY** **SINGTSGVMS** **--------SI** **TPPYTQHSPL** **ILPNIKQEMP** **SPTGNMN---** **----------** **----------**

**AmiSoxBa**  **------QPSR** **QIFTGSLDSS** **HSFRAADVMN** **HN------RA** **LYTSQAFQPT** **LPSQIQQRIS** **SVDEPRG---** **----------** **----------**

**NveSoxB2**  **------PSSR** **QIFAGAMDST** **HSFRASDMMA** **G-------RP** **VYSSQGYQGA** **LHSQVQQRLS** **QVEDSRG---** **----------** **----------**

**HmaSox10**  **------HSQS** **SPTGAHQSYP** **SSTITSQIGT** **PV--------** **-----ITDST** **YRVSTSDYIN** **SKNYFSN---** **----------** **----------**

**CheSox10**  **GG----APSP** **TSSNGTRGYN** **TASLGSPNGS** **ST------GA** **TMADSNANTT** **YRPTSTEYMT** **SKSYYIN---** **----------** **----------**

**CheSox3**  **STTAATAVVA** **STAPPTAVTA** **AQFSVFPPGA** **AT------YM** **YSPFSFPYGA** **TIAGGAAAAY** **SPQAAAA---** **----------** **----------**

**HmaSoxB1**  **------SQLP** **SSSSSSVTAA** **TGLSAQYSVV** **HS-----GTP** **TYMYSPLGFP** **YLNGAAMGTY** **GGQPYSH---** **----------** **----------**

**NveSox1**  **------SYPY** **DISSLTAASH** **SSLTGSRLER** **GL-----EIP** **SAVRPDISSM** **YTHGMYPTVP** **TSSALLN---** **----------** **----------**

**CheSox2**  **------IQHP** **GSTREGHPVH** **YPTTAREV--** **----------** **-RYYRPRSPD** **YNGRSRSPVD** **RDREYRH---** **----------** **----------**

**HmaSox3**  **------IAPK** **RPSSIPLADY** **TQSRSVPT--** **----------** **-------AVP** **VYTGFHRRTP** **EYRPHA----** **----------** **----------**

**NveSox2**  **------PPPP** **YHFSPHYSFV** **QNISDYKNQC** **----------** **--------GS** **HLSLMSRDLP** **YPSPIGY---** **----------** **----------**

**AquSOXB1**  **TGPLQQSCIV** **NSYNDPLNTY** **SAAMSVRSNS** **--------SL** **ASVLTSPTSL** **VFSGMDSALG** **SPPKASS---** **----------** **----------**

**EmuSox2**  **LCATETLQRL** **GTDPIPTYRC** **LSASALPTWI** **RGLVVXRKAW** **VXPVESVDPC** **TMLGKDDSVA** **SADSSGE---** **----------** **----------**

**AquSoxB2**  **PIIQLAHPSH** **LTSSPHQLVP** **LIQAASGTHA** **GM------HP** **TDGAKQGGQT** **ILIKMDAGTP** **HQVISPSYIT** **AAAAAGNRPS** **EQHCEVVEYP**

**CheSox13**  **------GYAM** **YAAPAPTAVS** **TADQSRPS--** **----------** **----------** **----------** **----------** **----------** **----------**

**HmaSox1**  **------GRQP** **MISPHSQMRH** **SPLSVGSPGN** **SH--------** **-DFYTLGSMQ** **TPRSASDMIP** **MSDRYSI---** **----------** **----------**

**CheSox14**  **----------** **----------** **----------** **----------** **----------** **YGSIARQAMT** **APTVVRG---** **----------** **----------**

**HmaSoxBb**  **------SAAP** **TTTPSAADYY** **SLNKNASPIA** **AG------ND** **YYGAVSSKNE** **YYSGLHSSTA** **ETRAERS---** **----------** **----------**

**NveSox3**  **------PPPP** **HSPPVHELAY** **SSVRSADMLV** **----------** **----SPNAHI** **YGGPMDPSTP** **TTSSMSA---** **----------** **----------**

**AmiSoxBb**  **------TPPS** **HANSVHELPY** **PVRPSEMMIP** **--------TP** **TGPHGHPSHI** **YGSTMESGPT** **TSGVSAF---** **----------** **----------**

**550 560 570 580 590 600 610 620 630**

**CinSoxB1**  **----------** **----------** **---SSVTSAA** **ASSGGSPLHQ** **QLQSMLPLHS** **H--------Q** **LPISQMYLPI** **SEQQPASATT** **PTDPSHSPGL**

**DmeSoxN**  **PSPSAIALNN** **NNNINNNHIM** **KREYSSAAAA** **AAAAAAAAAA** **GGGELNHLMN** **MYHLPDEQRH** **LLHYQTDSPD** **LQQQHQSMQQ** **QQQHLPQQHL**

**HSASox1**  **----------** **----------** **----------** **-----AAAAA** **SSGALGALGS** **LVKSEPSGSP** **PAPAHSRAPC** **PGDLREMISM** **YLPAGEGGDP**

**CelSoxB1**  **----------** **----------** **----------** **----------** **----------** **----------** **----------** **---GTVDSSQ** **FRAYYDHTKD**

**HSASox3**  **----------** **---GQQPATA** **AAAAAAAAAM** **SLGPMGSVVK** **SEPSSPPPAI** **ASHSQRACLG** **DLRDMISMYL** **PPGGDAADAA** **SPLPGGR---**

**HSASry**  **----------** **----------** **----------** **----------** **----------** **----------** **----------** **-----PPINA** **ASSPQQR---**

**PpiSox3**  **----------** **----------** **----------** **----------** **----------** **----------** **----------** **---------Q** **DLSPMYR--P**

**HSASox15**  **----------** **----------** **----------** **----------** **----------** **----------** **----------** **----NPPLAG** **APMPLTHL--**

**HSASox2**  **----------** **----------** **SSSPPVVTSS** **SHSRAPCQAG** **DLRDMIS---** **----------** **----------** **---MYLPGAE** **VPEPAAPSRL**

**HSASox14**  **----------** **----------** **----------** **----------** **----------** **----------** **----------** **--VVPCNCTA** **WSASTLQ--P**

**DmeSox21b**  **----------** **----------** **--------NN** **NSSSGLDDRD** **ATPQLEAVES** **KPHLHSPSDV** **GLDYAQYAQQ** **YGGQLAAAAG** **GAVGGGAAGA**

**DmeSoxdich** **----------** **----------** **----------** **----------** **----------** **----------** **-------HSA** **NAAGLYPSSS** **TSSPGSSPGT**

**DmeSox21a**  **----------** **----------** **----------** **-------LQA** **RLQAMYA---** **----------** **---GSIYHPW** **RYLPLISPET** **PPSPPSSSGT**

**HSASox21**  **----------** **----------** **----------** **-HTHSHPSPG** **NPGYMIP---** **----------** **----------** **-----CNCSA** **WPSPGLQ--P**

**CinSoxB2**  **----------** **---PNLLYTF** **NETEEKYETH** **NFRRRLPMWI** **TTSLASVSRS** **SSHGSRCYEQ** **RLCDEHTRCW** **LVVAFPAKPN** **PINAESLCLH**

**CelSoxB2**  **----------** **----------** **----------** **----------** **----------** **----------** **----------** **----------** **----------**

**AmiSoxB1**  **----------** **----------** **----------** **---RGRSCTD** **QLGDMIN---** **----------** **-----TYLPG** **ESAAASANAN** **HHPVTAS--H**

**NveSoxB1**  **----------** **----------** **----------** **VGGVTRNCAD** **QLGDMIN---** **----------** **-----TYLPP** **ADTANPVSTV** **NGLPSQT--N**

**AmiSoxBa**  **----------** **----------** **----------** **----------** **----------** **----------** **----------** **--GSLTNGSP** **SPTASSSEAP**

**NveSoxB2**  **----------** **----------** **----------** **----------** **----------** **----------** **-------MKS** **MNATPSPPVS** **SPDPMSKAYS**

**HmaSox10**  **----------** **----------** **-------MNS** **PYSPVDSAAT** **STHSQNR---** **----------** **-------YPT** **TDENRNIVNH** **AHSVGNGMVS**

**CheSox10**  **----------** **----GGQYSP** **LPAANATQGH** **SQQPQAARYP** **SPDDESRISQ** **SVVSQSNSDA** **ATTGSILMPK** **SVNGSAEEHS** **PSPPTYPSTN**

**CheSox3**  **-----YPHTI** **AVKQESAVSP** **EKTSGEASRT** **TFATAASIAA** **HYPGIYSVPI** **AIDQNSNPIG** **FDPKMHYATM** **AVAQERVAAT** **GSHPGSPARS**

**HmaSoxB1**  **----------** **---ALTLKPD** **TSSITVSTEK** **ATSHLSMHHD** **AVRSTIAPSV** **YYPIGIYPTA** **VDHNCNLTYD** **PKIPYATMTL** **LEQPHGNVKR**

**NveSox1**  **----------** **----------** **----------** **----------** **----------** **----------** **----------** **ASAVPVPGAG** **SPHPHYHAVQ**

**CheSox2**  **----------** **----------** **----------** **----------** **----------** **----------** **---------S** **PGVVYRSYSP** **PPQPREAYYY**

**HmaSox3**  **----------** **----------** **----------** **----------** **----------** **----------** **----------** **----------** **----------**

**NveSox2**  **----------** **----------** **----------** **----------** **----------** **----------** **----------** **------PSHG** **ASHPVQFVHR**

**AquSOXB1**  **------PVES** **LDSYSEVILS** **NCKVSDDCTS** **IHSNDSGAES** **DLRNMIS---** **---------T** **YLEESNSXPG** **PTETPPPSGS** **SRPPTAEFKL**

**EmuSox2**  **--------ND** **INSFINVYXR** **RHDRSRWTGG** **TVFQTACIVN** **QLRFFICSDV** **PREQRDLAGL** **GWEHCPFAAS**

**AquSoxB2**  **HHKQDIIDQD** **ESSSSSLSAQ** **STPTSKTTPV** **IVSEIKSHST** **PQVNSPSSSL** **LPPLLIPGGQ** **AGVNVLMQHA** **GGTQALQGQQ** **PGVVTVG--T**

**CheSox13**  **----------** **----------** **----------** **----------** **----------** **----------** **----------** **----------** **----------**

**HmaSox1**  **----------** **----------** **----------** **----------** **----------** **----------** **----------** **---MNIAVTQ** **GNMPSYSSSS**

**CheSox14**  **----------** **----------** **----------** **----------** **----------** **----------** **----------** **--------TQ** **PPPSTTPFRI**

**HmaSoxBb**  **----------** **----------** **----------** **-SSGSGADII** **NLSDRYP---** **----------** **-----SSESR** **QFENRYASSF** **IGGPGVSGSS**

**NveSox3**  **----------** **----------** **----------** **----------** **----------** **----------** **----------** **---FASSQHM** **QQPPSNRDKI**

**AmiSoxBb**  **----------** **----------** **----------** **----------** **----------** **----------** **----------** **------TNAT** **QNIHAQQ--I**

**I**

**640 650 660 670 680 690**

**CinSoxB1**  **SRLNSIGP--** **----------** **-------GTH** **YPISTSMGQL** **PMHMPAHM--** **---------**

**DmeSoxN**  **SQQHQQIP--** **--------QQ** **HHTMQQQQQQ** **HHLQHQQSLR** **AMAPLAHM--** **---------**

**HSASox1**  **AAAAAAAA--** **----------** **-QSRLHSLPQ** **HYQGAGAGVN** **GTVPLTHI--** **----------**

**CelSoxB1**  **QMMYPYSI--** **----------** **--ELTHAQNI** **QNALSQSHVT** **S---------** **---------**

**HSASox3**  **--LHGVHQ--** **----------** **----------** **HYQGAGTAVN** **GTVPLTHI--** **----------**

**HSASry**  **----DRYS--** **----------** **----------** **HWTKL-----** **----------** **----------**

**PpiSox3**  **QPVTTTTT--** **---------P** **TLNLKVEPQQ** **HLPVK-----** **----------** **---------**

**HSASox15**  **----------** **----------** **----------** **----------** **----------** **---------**

**HSASox2**  **HMSQHYQS--** **----------** **----------** **GPVP-GTAIN** **GTLPLSHM--** **----------**

**HSASox14**  **PVAYILFP--** **----------** **----------** **GMTKTGIDPY** **SSAHATAM--** **----------**

**DmeSox21b**  **AGGSAGGG--** **----------** **----------** **SGGATAADFR** **RPLTVIF---** **----------**

**DmeSoxdich** **ITPNGMDG--** **----------** **----------** **SM---DSALR** **RPVPVLY---** **----------**

**DmeSox21a**  **GISSYGCV--** **----------** **------KSEK** **SSPNAVVASA** **ASPPNII---** **---------**

**HSASox21**  **PLAYILLP--** **----------** **------GM--** **-GKPQLDPYP** **AAYAAAL---** **---------**

**CinSoxB2**  **TGARVFALPS** **QPAKKWTRLL** **FPASLTGDV-** **GGRKSAEKTG** **LGR-------** **---------**

**CelSoxB2**  **----------** **----------** **----------** **----------** **----------** **---------**

**AmiSoxB1**  **HIPPNRYSQ-** **WQEQSSVANS** **NLPVHSGVPA** **PSISSVSGVS** **GTIPLTHMP-** **---------**

**NveSoxB1**  **TISS-RYSQH** **WQDQNTI--S** **NMPNHPTL-A** **GNISSISGVR** **GTMPLSHM--** **---------**

**AmiSoxBa**  **SKSTAGYTVS** **TAE---LSAA** **QRVWHLV---** **----------** **----------** **---------**

**NveSoxB2**  **SSELSNQRVW** **QPQQDLTRTV** **AYVPVLL---** **----------** **----------** **---------**

**HmaSox10**  **KLIQEHDVQS** **NFPDNAVNRN** **WSLNFSSV--** **----------** **----------** **---------**

**CheSox10**  **TPVGRHWTPS** **GGSGAPTQDM** **TSGNFH----** **----------** **----------** **---------**

**CheSox3**  **TPNSTTASEH** **DKINSQSNQN** **RLLYYIHV**

**HmaSoxB1**  **EQTREGTTRN** **ISDDHQSRRV** **NTPTYSISSN** **TATQ------** **----------** **---------**

**NveSox1**  **GANGQYSVPC** **NCTWQPQDLR** **RPVAYLLL--** **----------** **----------** **---------**

**CheSox2**  **QSRSEAKQNS** **YHDDEASDGE** **K---------** **----------** **----------** **---------**

**HmaSox3**  **----------** **----------** **----------** **----------** **----------** **---------**

**NveSox2**  **SLIPDSGSVV** **HATSPIDARS** **GLARHPLDCV** **VMRPEGY---** **----------** **---------**

**AquSOXB1**  **LNASAQCT--** **--------DF** **IASNSSNXTN** **SAESLLDGAG** **GTLPLQHLM-** **---------**

**EmuSox2**

**AquSoxB2**  **GGSPHHHIGA** **LRSAEIPYRV** **YPDLSSMPLC** **LVPVVDQASP** **GSNYSGSR--** **---------**

**CheSox13**  **----------** **----------** **----------** **----------** **----------** **---------**

**HmaSox1**  **PAIHIPHESG** **SPVGYQMYSS** **AERSL-----** **----------** **----------** **---------**

**CheSox14**  **HQIIPTQQ--** **----------** **----------** **----------** **----------** **---------**

**HmaSoxBb**  **VVVPTYTSIQ** **NIPHINTESP** **AAYSYSSLYE** **QRH-------** **----------** **---------**

**NveSox3**  **TTLRRIRKPY** **LHKDTCDFTV** **ANLLYSLSCV** **TNGFYKTTMT** **F---------** **---------**

**AmiSoxBb**  **AEASPQYPQL** **YTQRHV----** **----------** **----------** **----------** **---------**

**II**
