# Supplementary material for: Multiple Sox genes are expressed in stem cells or in differentiating neuro-sensory cells in the hydrozoan Clytia hemisphaerica
Source: EvoDevo. 2011 Jun 1;2:12. doi: 10.1186/2041-9139-2-12 (PMC3120710; doi:10.1186/2041-9139-2-12)
Supplement: Additional file 2 — Alignment of group C Sox amino-acid sequences. Legend as for Additional file 1. [file 2041-9139-2-12-S2.DOC]

Additional file 2

**10 20 30 40 50 60 70 80 90**

**CheSox12** **----------** **-MEVD-----** **----------** **---------V** **GMDFFLCETL** **-------PTP** **EPQDGDLNMM** **QHL-------** **----------**

**HmaSox4**  **----------** **-MELD-----** **----------** **---------F** **GLDYFMSGAL** **-------PTP** **EPQDGDI---** **KHL-------** **----------**

**NveSoxC**  **----------** **----------** **----------** **----------** **MMMVLQNSEF** **----RASPSP** **PHSLS-----** **----------** **----------**

**AmiSoxC**  **----------** **----------** **----------** **----------** **-MMVFHGSEL** **-----NSPSP** **PPQV------** **----------** **----------**

**PpiSOX12** **----------** **----------** **----------** **----------** **----------** **----------** **----------** **----------** **----------**

**HSASox4**  **MVQQTNNAEN** **TEALL-----** **----------** **---------A** **GESSDSGAGL** **ELGIASSPTP** **----------** **----------** **----------**

**HSASox11** **MVQQAESLEA** **ESNLP-----** **----------** **---------R** **EALDTEEGEF** **----------** **----------** **----------** **----------**

**HSASox12** **MVQQ------** **----------** **----------** **---------R** **GARAKRDGGP** **-------PPP** **GP--------** **----------** **----------**

**DmeSoxC**  **MIAKPNQATT** **EPPLSLRPGT** **VPTVPATTPA** **RPATITIQRR** **HPAPKADSTP** **HTLPPFSPSP** **SPASSPSPAP** **AQTPGAQKTQ** **SQAAITHPAA**

**CinSoxC**  **MASTSRLTKI** **SAELM-----** **-SLHAGQNLD** **GNPGSPESGL** **GSDWDFDSDE** **IVQSSSVATP** **RRSIDALHAS** **SPSPTLECIL** **DCPDVSGVDV**

**CelSoxC**  **----------** **-MDLQ-----** **----------** **---------K** **PPNFMLDCGM** **--APHMMPPI** **NWAAAAIAVA** **SSTSGATNAT** **SSNSVATSQQ**

**AquSoxC**  **--------MS** **AGDLQ-----** **----------** **---------Y** **TVILQQAPSP** **SCSSLSSPSS** **PYQSNLLDVC** **D---------** **----------**

**100 110 120 130 140 150 160 170 180**

**CheSox12** **----------** **----------** **----------** **-----NPLEL** **SVGDSNSQRP** **IK--------** **----------** **----------** **----RKKSGN**

**HmaSox4**  **----------** **----------** **----------** **-----NPLEL** **NI---DFQLG** **CG--------** **----------** **----------** **----NKKKSS**

**NveSoxC**  **----------** **----------** **----------** **-----GGMDD** **QVDATQATHE** **SK--------** **----------** **----------** **----KKSDM-**

**AmiSoxC**  **----------** **----------** **----------** **-----PSVDE** **AE---EEPKK** **SM--------** **----------** **----------** **----Q-----**

**PpiSOX12** **----------** **----------** **----------** **----------** **----------** **----------** **----------** **----------** **----------**

**HSASox4**  **----------** **----------** **----------** **-----GSTAS** **TGGKADDPSW** **CK--------** **----------** **----------** **----TPS---**

**HSASox11** **----------** **----------** **----------** **-----MACSP** **VALDESDPDW** **CK--------** **----------** **----------** **----TAS---**

**HSASox12** **----------** **----------** **----------** **-----GPAEE** **GA---REPGW** **CK--------** **----------** **----------** **----TPS---**

**DmeSoxC**  **VASPSAPVAA** **ASTEDPQDPG** **PRSTHTHTHS** **QHFSPPPRES** **EMDGERSPSH** **SGHEMTLSMD** **GIDSSLVFGS** **ARVPVNSSTP** **YSDATRTKKH**

**CinSoxC**  **FDSPIKKEAD** **FKDPKPRQSP** **NKNKRENILN** **ELKNLPATKI** **KL---PPPSR** **SS--------** **----------** **----------** **----QKKLAA**

**CelSoxC**  **LQHHPYGTAA** **GGYKHAQ---** **----------** **-----QAIPK** **SVTPYSDATN** **CK--------** **----------** **----------** **----KSS---**

**AquSoxC**  **----------** **----------** **----------** **-----VGTAT** **HVHHNGGSRG** **GG--------** **----------** **----------** **----NGGSAG**

**190 200 210 220 230 240 250 260 270**

**CheSox12** **MTDHVKRPMN** **AFMVWSQIER** **KKMAESYPDM** **HNAEISRRLG** **KQWKMLTDDD** **RRPYVIRSEK** **LREEHMRRHP** **DYKYRPKKKA** **K---------**

**HmaSox4**  **M-DHVKRPMN** **AFMVWSQIER** **KKMADIYPDM** **HNAEISRRLG** **KRWKLLSDAD** **RRPFVIRSEK** **LREEHMRRYP** **DYKYRPKKKA** **K---------**

**NveSoxC**  **--QHVKRPMN** **AFMVWSQIER** **RKMAEEHPDM** **HNAEISKRLG** **KRWKLLSESE** **KRPFVEESER** **LRIRHMQAYP** **DYKYRPRKKK** **Q--------P**

**AmiSoxC**  **---HVKRPMN** **AFMVWSQIER** **RKMAEEHPDM** **HNAEISKRLG** **KRWKLLSESE** **KRPFVEESER** **LRIRHMQAYP** **DYKYRPRKKK** **Q--------P**

**PpiSOX12** **----------** **---VWSQIQR** **AKIVEEQPNK** **HNAAISKQLG** **SEWKMLSDEA** **RMPYIHESQR** **LKRIHKQQYP** **DYKYRPRKRG** **K--------G**

**HSASox4**  **--GHIKRPMN** **AFMVWSQIER** **RKIMEQSPDM** **HNAEISKRLG** **KRWKLLKDSD** **KIPFIREAER** **LRLKHMADYP** **DYKYRPRKKV** **KSGNANSSSS**

**HSASox11** **--GHIKRPMN** **AFMVWSKIER** **RKIMEQSPDM** **HNAEISKRLG** **KRWKMLKDSE** **KIPFIREAER** **LRLKHMADYP** **DYKYRPRKKP** **K------MDP**

**HSASox12** **--GHIKRPMN** **AFMVWSQHER** **RKIMDQWPDM** **HNAEISKRLG** **RRWQLLQDSE** **KIPFVREAER** **LRLKHMADYP** **DYKYRPRKKS** **K------GAP**

**DmeSoxC**  **SPGHIKRPMN** **AFMVWSQMER** **RKICERTPDL** **HNAEISKELG** **RRWQLLSKDD** **KQPYIIEAEK** **LRKLHMIEYP** **NYKYRPQKKQ** **T------RSP**

**CinSoxC**  **RPGYIKRPMN** **AFMIWSQIER** **RKIMEKTPEL** **HNAEISRNLG** **RIWREQADSI** **KRPFLIEAER** **LRLQHMCDYP** **DYKYKPKKKA** **K---------**

**CelSoxC**  **--NHIKRPMN** **AFMVWSQMER** **RKICEHQPDM** **HNAEISKQLG** **SRWRSLTDEE** **KAPFVAEAER** **LRVCHMQEYP** **DYKYKPRKKP** **K------KNP**

**AquSoxC**  **KKEHIKRPMN** **AFMVWAQLER** **RKMTTEFPDM** **HNAEISRRLG** **KLWRLLSDRE** **KQPYIEESER** **LRIQHMKQYP** **DYKYRPRKKG** **G------KKP**

**HMG domain**

**280 290 300 310 320 330 340 350 360**

**CheSox12** **----------** **----------** **----------** **-------EME** **GKQGAATQTN** **QQRANNTKNN** **----------** **----------** **----------**

**HmaSox4**  **----------** **----------** **-------ELA** **AKELAAKELK** **NFSKVCEYNI** **VTLTNGRNDD** **----------** **------W---** **----------**

**NveSoxC**  **AKAKP-----** **----------** **-------GDA** **KPAASEQS--** **-----PRKNL** **TVTALGTKRE** **----------** **----------** **----------**

**AmiSoxC**  **AKQKN-----** **----------** **-------GGA** **QDSKTSSSNS** **DGHSHSRKHI** **AETVGTKRE-** **----------** **----------** **----------**

**PpiSOX12** **TTTTTTGVTT** **KENTAPSVNI** **TRXGPVQLPP** **RSTTPDVHHS** **KPQTTPRHQA** **VIVREQRSTA** **---------S** **YTVPFIK---** **----------**

**HSASox4**  **AAASSKPGEK** **GDKVGGSGGG** **GHGGGGGGGS** **SNAGGGGGGA** **SGGGANSKPA** **QKKSCGSKVA** **GGAGGGVSKP** **HAKLILA---** **----------**

**HSASox11** **S-AKPSASQS** **PEKSAAGGGG** **GSAGGGAGGA** **KTSKGSSKKC** **GKLKAPAAAG** **AKAGAGKAAQ** **SGDYGGAGDD** **YVLGSLRVSG** **SGGGGAGKTV**

**HSASox12** **AKARP-----** **----------** **-RPPGGSGGG** **SRLKPGPQLP** **GRGGRRAAGG** **PLGGGAAAPE** **----------** **----------** **----------**

**DmeSoxC**  **GSLKP-----** **----------** **----NQDADG** **CEARNDTTNN** **NNSLTTLAIN** **GTTTAGRKSK** **----------** **----------** **----------**

**CinSoxC**  **----------** **----------** **-------GKK** **CDSSENSTFS** **YLHNDPDQSM** **EIIETNTEAL** **----------** **----------** **----------**

**CelSoxC**  **DGTLQ-----** **----------** **-------QPA** **QPQAPQQQQA** **PPRGASPQAR** **QRKRPNTDQQ** **----------** **------S---** **----------**

**AquSoxC**  **KPVSN-----** **----------** **----TSYLGG** **NDSGSEEYYP** **TTMPTSSSNS** **CSCGAGIRRA** **-------PVP** **TCSIAVQ---** **----------**

**370 380 390 400 410 420 430 440 450**

**CheSox12** **----------** **----------** **--------TN** **TLLAIEGNS-** **----------** **-~~~~~~YDS** **TMIKLGPSTT** **LAYNALNSKH** **LL--------**

**HmaSox4**  **----------** **----------** **---DKSKYTN** **NVACCENNKA** **PPNKT-----** **-------FVT** **ISRQVGQIGS** **LSNITVSPQK** **CIDIPPS---**

**NveSoxC**  **----------** **----------** **----------** **---------A** **LPGAQMGS--** **-------YYG** **-SSSAKKFNS** **MSEPTYKKQR** **RDLG-P----**

**AmiSoxC**  **----------** **----------** **----------** **---------A** **LPGAH-----** **------GYPG** **LGSSKKQATS** **HGEAPFKKQR** **RDFASP----**

**PpiSOX12** **----------** **----------** **---RKRTLKI** **ERVTEEAAGR** **FEFSEPAVKK** **RPTSVKRF--** **VAVQLDSSKS** **TTGQGGVKRF** **RILTTTSNNT**

**HSASox4**  **----------** **----------** **---GGGGGGK** **AAAAAAASFA** **AEQAGAAALL** **PLGAAADHHS** **LYKARTPSAS** **ASASSAASAS** **AALAAPGKHL**

**HSASox11** **KCVFLDEDDD** **DDDDDDELQL** **QIKQEPDEED** **EEPPHQQLLQ** **PPGQQPSQLL** **RRYNVAKVPA** **SPTLSSSAES** **PEGASLYDEV** **RAGATSGAGG**

**HSASox12** **----------** **----------** **---DDDEDDD** **EELLEVRLVE** **TPGRELWR--** **------MVPA** **GRAARGQAER** **AQGPSGEGAA** **AAAA------**

**DmeSoxC**  **----------** **----------** **----------** **----RSTSTC** **QSGSA-----** **----------** **SKRLRNDSGD** **TSSKPKYEVK** **MESAEQL---**

**CinSoxC**  **----------** **----------** **----------** **KAEVKVSSKR** **LPKKRKLSQS** **KPSLPEIKPT** **VVASINPIQN** **NSGNSEPQAK** **EVTATTNNLL**

**CelSoxC**  **----------** **----------** **---ETQQFQN** **FKSVKVEQDW** **MGNAHMSHAQ** **KMPFHPSYPS** **-PSEFGHAPL** **TPESGFYDDY** **FTQQHHQQHF**

**AquSoxC**  **----------** **----------** **---CSMELGE** **HVIEREPSSP** **KQTAEISI--** **-QVGNGSAHL** **QQNRRRSFSS** **FAGDKRPRDL** **SLSCPPIKKR**

**460 470 480 490 500 510 520 530 540**

**CheSox12** **----------** **---KPIKQAN** **TTNAANVPLQ** **RIESAKLVNI** **KNGQC----V** **MTTGKPFLTL** **SRQVGKIGS~** **~~LSSSEGNF** **SISPQKNSLL**

**HmaSox4**  **----------** **---TLSPPSV** **VPDCCGTPPD** **EYNSESFYNY** **DSFSG-----** **----KNNSNS** **----------** **----------** **----------**

**NveSoxC**  **----------** **---ITPPPNV** **PDSIGVTPEE** **PIDQLSLYED** **FDQAF-----** **----KTEQNA** **------QG--** **--INSNHQQA** **AHNNHQIPSG**

**AmiSoxC**  **----------** **---ITPPPNV** **PDAIGVTPDD** **TIDQLSLYED** **FEHAF-----** **----KQDQQV** **------PS--** **--INHT----** **----QQMPNS**

**PpiSOX12** **TTNLFNNQ--** **---KQPFTLT** **PPSSLALSSQ** **PSKRPRLVDC** **SPALP-----** **----HEREII** **KELRGGGY--** **--LSQTCGST** **MKSEDILSSK**

**HSASox4**  **AEKKVKRVYL** **FG-GLGTSSS** **PVGGVGAGAD** **PSDPLGLYEE** **EGAGCSPDAP** **SLSGRSSAAS** **SPAAGRSP--** **--ADHRGYAS** **LRAASPAPSS**

**HSASox11** **GSRLYYSFKN** **ITKQHPPPLA** **QPALSPASSR** **SVSTSSSSSS** **GSSSG-----** **----SSGEDA** **DDLMFDLS--** **--LNFSQSAH** **SASEQQLGGG**

**HSASox12** **----------** **---ASPTPS-** **----------** **EDEEPEEEEE** **EAAAA-----** **----EEGEEE** **TVASGEES--** **--LGFL----** **----SRLPPG**

**DmeSoxC**  **----------** **---NSADIIL** **PSADNLISYQ** **SSEYLPLSTL** **SNADC-----** **------DEKL** **HSELSSGP--** **--LESRENLS** **EVVNRFLPLF**

**CinSoxC**  **TAVKVE----** **---PRVPPLP** **ITPATIVTAV** **PSKRGRFEVH** **TPSPC-----** **----DTNQRI** **GLVHGTVFKT** **VTEGNRQFII** **VSGANDVISD**

**CelSoxC**  **ASQ-------** **---HHNSAGS** **PLRMTNLGMD** **MGMPPQMMGH** **NSGFG-----** **----AGNHPF** **YLHTSPPS--** **--VDQDDMRS** **LSSGSSGYAD**

**AquSoxC**  **----------** **---ALIPVSS** **PPEVLPTSLD** **DIFPPSPPSS** **DSSPI-----** **----SHQHRE** **DTLPTLHF--** **--EEFMDPLM** **PYDPSAVDLA**

**I**

**550 560 570 580 590 600 610 620 630**

**CheSox12** **K--------- ---------- ---------- ---------- ---------- ---------- ---------- ---------- ----------**

**HmaSox4**  **----------** **----------** **----------** **----LYHEDM** **DLLINFARFT** **----------** **----------** **DGLPDLYTTP** **EVSEMLTGQW**

**NveSoxC**  **WPNLELST--** **----------** **----------** **----LNLNSL** **PVSPLFGLDG** **----------** **-----ANGGQ** **FDFPDLYTPP** **EVSELIQGQW**

**AmiSoxC**  **WPNLEFGN--** **----------** **----------** **----MNFNGL** **PVSPLLDLHG** **----------** **-----TSAGQ** **FDFPDLYTPP** **EVSELIQGQW**

**PpiSOX12** **VQ--------** **----------** **----------** **----LDNLYF** **DSHTDYFSGS** **----------** **-----LNEKD** **LSLSD-YTTE** **EVWNLIRVE-**

**HSASox4**  **APSHASSSAS** **SHSSSSSSSG** **SSSSDDEFED** **DLLDLNPSSN** **FESMSLGSFS** **SSSALDRDLD** **FNFEPGSGSH** **FEFPD-YCTP** **EVSEMISGDW**

**HSASox11** **AAAGN-----** **----------** **----------** **----LSLSLV** **DKDLDSFSEG** **----------** **-----SLGSH** **FEFPD-YCTP** **ELSEMIAGDW**

**HSASox12** **PAG-------** **----------** **----------** **----LDCSAL** **DRDPDLQPP-** **----------** **-----SGTSH** **FEFPD-YCTP** **EVTEMIAGDW**

**DmeSoxC**  **LGGNEDSQ--** **----------** **----------** **----LGVSSL** **TQSQHNQSDP** **----------** **-----TAGLM** **DNISDISPIN** **DREELTEEVM**

**CinSoxC**  **VTNSSLVSTQ** **RTSTPCQQKQ** **I---------** **----INANFV** **PITNDCGQEK** **VYN-------** **-----IVRGC** **IQVASQEPTN** **STTQKRVEPD**

**CelSoxC**  **CSASEQST--** **----------** **----------** **----SSPNSA** **GVVTMATAAT** **----------** **-----TTTHL** **DDLEQICPTV** **TTGELVNYPW**

**AquSoxC**  **LSGSSSAL--** **----------** **----------** **----SSSTAA** **SNVLSLNTFP** **LQSSAATSIF** **SPFNIDSCST** **FDFPEL--PS** **DFADIFAQNA**

**II**

**640**

**CheSox12** **----------** **--------**

**HmaSox4**  **LENDLGF---** **--------**

**NveSoxC**  **LENSLDQL--** **--------**

**AmiSoxC**  **LENSLGHL--** **--------**

**PpiSOX12** **APFTTTALTC** **CSCKILMF**

**HSASox4**  **LESSISNLVF** **TY------**

**HSASox11** **LEANFSDLVF** **TY------**

**HSASox12** **RPSSIADLVF** **TY------**

**DmeSoxC**  **VPALPGGESI** **ERRAHP--**

**CinSoxC**  **VPTTFANMGD** **DFRNEYLK**

**CelSoxC**  **SDALGIDINF** **S-------**

**AquSoxC**  **SEFDTSITTL** **LST-----**
