# Supplementary material for: Multiple Sox genes are expressed in stem cells or in differentiating neuro-sensory cells in the hydrozoan Clytia hemisphaerica
Source: EvoDevo. 2011 Jun 1;2:12. doi: 10.1186/2041-9139-2-12 (PMC3120710; doi:10.1186/2041-9139-2-12)
Supplement: Additional file 3 — Alignment of group F Sox amino-acid sequences. Legend as for Additional file 1. [file 2041-9139-2-12-S3.DOC]

Additional file 3

**10 20 30 40 50 60 70 80 90**

**CheSox11** **----------** **----------** **----------** **----------** **----------** **-----SKEMA** **SKHQTQTLKE** **LLQKPNMQTE** **FNKAYS----**

**AmiSoxF**  **----------** **----------** **----------** **----------** **---MHEVDRM** **L-------LS** **DRRTERNKRV** **PAPLSSMRVL** **SPSGFNNSI-**

**NveSoxF1** **----------** **----------** **-----MRAPE** **----------** **----------** **----------** **ELKLEPDEKR** **IAKGKRFVHL** **PPITIPNAT-**

**DmeSoxF**  **MEPSYDHEHP** **HRLLTNYNSK** **KYPHVSRTPE** **YSHSTGSDYP** **EHGGYLTDGR** **LMHESNSDAG** **IYHVRQGSEH** **SSPSLHSPAI** **QSSGYENEHL**

**HSASox7**  **----------** **----------** **----------** **----------** **----------** **----------** **----------** **-----MASLL** **GAYPWPEGL-**

**HSASox17** **----------** **----------** **-----MSSPD** **----------** **--AGYASDD-** **----------** **-----QSQTQ** **SALPAVMAGL** **GPCPWAES--**

**HSASox18** **----------** **----------** **----MQRSPP** **----------** **---GYGAQDD** **P---------** **----------** **-------PAR** **RDCAWAPGH-**

**CinSoxF**  **MTGLLHVVPP** **NNAGSGTLPY** **TIPSDEYSRS** **LTSFVDP-YS** **SFSGYSPQRD** **YL--ESMGLL** **TRAVSGSSPE** **SSKSVSTPSI** **FTAGLNSGLF**

**100 110 120 130 140 150 160 170 180**

**CheSox11** **----LSPNGH** **FKYV------** **----------** **----------** **----------** **--KRQTSKMF** **Y-----HGTV** **HRSR------** **----------**

**AmiSoxF**  **----GCHSVQ** **PEQ-------** **----------** **----------** **----------** **--NQSYVKLL** **R-----GSQE** **QDSNP-----** **----------**

**NveSoxF1** **----FTQAAQ** **MRDR------** **----------** **----------** **----------** **--DLPYTNWL** **GSLSGSEGRA** **RTE-------** **----------**

**DmeSoxF**  **NEAVLAAHSH** **SHSPMPMVSS** **AYVGGGTASG** **SLINSNIPLL** **GGGGNSVLNK** **FLSHPHAGMV** **G-----GGTG** **QMEDCTSHSP** **IEAASMWSYD**

**HSASox7**  **----ECPALD** **----------** **----------** **----------** **----------** **-------AEL** **S-----DGQS** **PPAVP-----** **----------**

**HSASox17** **----LSPIGD** **MKV-------** **----------** **----------** **----------** **----------** **------KGEA** **PANSG-----** **----------**

**HSASox18** **----GAAADT** **----------** **----------** **----------** **----------** **-------RGL** **A-----AGPA** **ALAAP-----** **----------**

**CinSoxF**  **YSQYSTVTSS** **TTSP------** **----------** **----------** **----------** **--YTSSSQWL** **SPQVTNAGHY** **QQYACPYNLT** **NASQPSLAVN**

**190 200 210 220 230 240 250 260 270**

**CheSox11** **----------** **----------** **----------** **---LMPTKDR** **IRRPLNCFMV** **FSHLERKRVA** **EEHPELHNAD** **LSKILGKRWK** **TLSPSEKQPY**

**AmiSoxF**  **-KKELS----** **----------** **----------** **---MDSDTDR** **IKRPMNAFMV** **WAQVERRRLA** **DANPELHNAE** **LSKILGQAWR** **ALNGLQKRPF**

**NveSoxF1** **----------** **----------** **----------** **---KDDETER** **IKRPMNAFMV** **WAQVERRRLA** **DANPELHNAE** **LSKMLGLTWR** **ALNSTQKRPF**

**DmeSoxF**  **YKGDLCAPNC** **GYLERHKPLP** **ADLKYRPGGT** **QS-KSAKESR** **IRRPMNAFMV** **WAKIERKKLA** **DENPDLHNAD** **LSKMLGKKWR** **SLTPQDRRPY**

**HSASox7**  **----------** **----------** **-----RPPG-** **---DKGSESR** **IRRPMNAFMV** **WAKDERKRLA** **VQNPDLHNAE** **LSKMLGKSWK** **ALTLSQKRPY**

**HSASox17** **----------** **----------** **-----APAGA** **AG-RAKGESR** **IRRPMNAFMV** **WAKDERKRLA** **QQNPDLHNAE** **LSKMLGKSWK** **ALTLAEKRPF**

**HSASox18** **-AAPASPPSP** **QRSPPRSPEP** **GRYGLSPAGR** **GERQAADESR** **IRRPMNAFMV** **WAKDERKRLA** **QQNPDLHNAV** **LSKMLGKAWK** **ELNAAEKRPF**

**CinSoxF**  **DQTSYLSSYP** **ELYADTITNN** **TSVRSRKSTE** **TAKAKKDEPR** **IRRPMNAFMC** **WAKTERKRMA** **AAFPDHHNAE** **LSKMLGKKWK** **EMSNEDKRPY**

**HMG domain**

**280 290 300 310 320 330 340 350 360**

**CheSox11** **IEEAERIRQL** **HTEIYPDYKY** **QPRRKNQPKK** **----------** **----PGEESH** **Q--SESNTEL** **SSPTNTRSET** **----------** **--HSPSNNAM**

**AmiSoxF**  **VEEAERLRQQ** **HIKDHPDYKY** **RPRRRKHPKR** **VI-----KKM** **SSTNPAVCAL** **V----QERKD** **ASPRVSRLNP** **-------TTD** **CGRLPFNPSD**

**NveSoxF1** **VDEAERLRLQ** **HMQDYPNYKY** **RPRRRKHSKR** **AA-----KRS** **TGAAAGSKVN** **G--TACQKSS** **GQETVSRFNC** **----------** **---LVESSFF**

**DmeSoxF**  **VEEAERLRVI** **HMTEHPNYKY** **RPRRRKQSKL** **RAMQPGGKEQ** **SESSPNPGTG** **G--SKSNPKL** **ATPPLATASS** **SYTTPTDEST** **CNSTNQNHGQ**

**HSASox7**  **VDEAERLRLQ** **HMQDYPNYKY** **RPRRKKQAKR** **LC-----KRV** **D---PGFLLS** **S----LSRDQ** **NALPEKRSGS** **--------RG** **ALGEKEDRGE**

**HSASox17** **VEEAERLRVQ** **HMQDHPNYKY** **RPRRRKQVKR** **L------KRV** **E----GGFLH** **G--------L** **AEPQAAALGP** **--------EG** **GRVAMDGLGL**

**HSASox18** **VEEAERLRVQ** **HLRDHPNYKY** **RPRRKKQARK** **A------RRL** **E---PGLLLP** **G--------L** **APPQ------** **----------** **----------**

**CinSoxF**  **ITEAEKLRMK** **HMQEHPDYKY** **RPRRKPKEPK** **S------RRG** **KTTAADDGVT** **GDHFGGSTNC** **GKTLNTKLGT** **----------** **TGKLVNGKSI**

**370 380 390 400 410 420 430 440 450**

**CheSox11** **RSASSSNDMN** **LPPIPQPLTT** **PINH------** **----------** **-----QNPPN** **TS---YQLP-** **SYEGNLSAPN** **----------** **----------**

**AmiSoxF**  **GNPIGFAPLP** **KQTGFPSGEM** **KLSH------** **----------** **-----LGSPP** **VL-----PK-** **LPEISLLTPE** **----------** **----------**

**NveSoxF1** **QVPGTPNSPI** **PSPEPKPGKS** **QFSA------** **----------** **-----LTDPE** **VSMLPAPFPN** **LEDIDLPTPE** **----------** **----------**

**DmeSoxF**  **STPGGLYEQP** **LKPTYSPSSV** **DCYSNADSTE** **QIESLAANCP** **PALLNESSPT** **GGGYDNSLL-** **LKKLTKPSPS** **RAAKSRQEKL** **AKSEEKNKGS**

**HSASox7**  **YSPGTAL---** **------PSLR** **GCYH------** **----------** **-----EGPAG** **GGGGGTPSS-** **VDTYPYGLPT** **PPEMSPLDVL** **E---------**

**HSASox17** **QFPEQGF-PA** **GPPLLPPHMG** **GHYR------** **----------** **-----DCQSL** **GA-----PP-** **LDGYPLPTPD** **---TSPLDGV** **D---------**

**HSASox18** **-------PPP** **EPFPAASGSA** **RAFR------** **----------** **-----ELPPL** **GA------E-** **FDGLGLPTPE** **---RSPLDGL** **E---------**

**CinSoxF**  **YSANSLFSSD** **QAHVRRNHSN** **NNSR------** **----------** **-----HGNKY** **GEGFSNSFS-** **FYDSTASFPY** **----------** **----------**

**460 470 480 490 500 510 520 530 540**

**CheSox11** **----------** **---------T** **PILTSGSHQE** **YPTNNNEYID** **YTEHPLPQSF** **PPTPTI----** **-SPAAVDHQR** **PMFVFENLQN** **AFPQSKQSSP**

**AmiSoxF**  **----------** **----------** **--PSPGSS--** **----------** **----EFGSEF** **NFPSGW--QD** **LPNAPVNLQP** **KTLQSTSPAS** **YVGVSTANST**

**NveSoxF1** **----------** **----------** **--SSPGRA--** **----------** **----ETSKVF** **TFPTAA----** **-VAAAVEFRS** **TLLGMLASQS** **ASVHSTHNTS**

**DmeSoxF**  **QSQGQSQQGI** **YAATYPLAPT** **SVAVVAARGM** **YVTCNNRGLL** **DHGHSVKGTF** **YPPVSVSEDD** **NSTSMRNSIS** **ALQQHCNVVT** **STPSSSGGTM**

**HSASox7**  **----------** **-----P-EQT** **FFSSPCQEEH** **GHPRRIPHLP** **GH--PYSPEY** **APSPLH-CSH** **PLGSLALGQS** **PGVSMMSPVP** **GCPPSPAYYS**

**HSASox17** **----------** **-----P-DPA** **FFAAPMPG--** **--DC------** **----PAAGTY** **SYAQVS---D** **YAGPPEPPAG** **PMHPRLGPEP** **AGPSIPGLLA**

**HSASox18** **----------** **-----PGEAA** **FFPPPAAPE-** **--DCALR---** **----PFRAPY** **APTELS----** **-RDPGGCYGA** **PLAEALRTAP** **PAAPLAGL--**

**CinSoxF**  **----------** **-----ASLES** **LFSNT-----** **----------** **----PNSSSY** **QIKHKS----** **----PGERMK** **SLFDSCSSTS** **KCDINFDGQA**

**550 560 570 580 590 600 610 620 630**

**CheSox11** **MTEQDYEANA** **----------** **----------** **----------** **-----IRWLA** **CDLQQNVDQV** **SSPQSYQSFN** **EPHNYQEPYH** **NGNPQQFYQQ**

**AmiSoxF**  **LASQLYFSNG** **SVNAMT----** **----------** **----------** **--------LN** **PSIAGSVNAA** **HLQNSVASSS** **QLFSSFHITE** **LIPEEDFNRE**

**NveSoxF1** **LFGTGNVSYS** **----------** **----------** **----------** **--------SQ** **SSIVNAQAST** **GPQQSIPLSD** **LLLDDL----** **-----NLNTN**

**DmeSoxF**  **PTSEMSSYTV** **SMADNCGNLR** **LSMNELSGNE** **YLPSANAYGM** **QYEDFLRYQS** **NDMDYSTSAV** **EHKETTSDSA** **SGQKCLKYPD** **TNQ--NYDDY**

**HSASox7**  **PAT----YHP** **----------** **----------** **----------** **--------LH** **SNLQAHLGQL** **SPPPEHPGFD** **-ALDQLSQVE** **LLG--DMDRN**

**HSASox17** **PPSALHVYYG** **AMGSPG----** **----------** **-AGGGRGFQM** **QPQHQHQHQH** **QHHPPGPGQP** **SPPPEALPCR** **DGTDPSQPAE** **LLG--EVDRT**

**HSASox18** **-------YYG** **TLGTPG----** **----------** **----------** **----------** **----PYPGPL** **SPPPEAPPLE** **SAEPLGPAAD** **LWA--DVDLT**

**CinSoxF**  **YTNRA-----** **----------** **----------** **----------** **--------NQ** **SSLFNYNTTV** **SSPQIRNPID** **--FSCFT---** **-----KYTAP**

**640 650 660 670 680 690 700 710 720**

**CheSox11** **NVQQQINMQ-** **----------** **--DYRNNIEN** **MNFQQQNFPL** **DANANNNM--** **----------** **----------** **----------** **----------**

**AmiSoxF**  **EFDQYLDGT-** **----------** **----------** **----------** **ETANISWM--** **----------** **----------** **----------** **----------**

**NveSoxF1** **ELDQYLDGT-** **----------** **----------** **----------** **ELDAFDYVI-** **----------** **----------** **----------** **----------**

**DmeSoxF**  **EAEAYSNAM-** **LPATAASYYT** **QLPYPPTSLA** **AFPLQLAVPF** **QQTTSGAYGA** **QPIQSGYLHY** **GNYGGYEGMA** **RVSHRTKRPS** **TISRHPTRLR**

**HSASox7**  **EFDQYLNTP-** **GHPDSATGAM** **ALSGHVPVSQ** **VTPTG---PT** **ETSLISV-LA** **DA-TATYYNS** **YSVS------** **----------** **----------**

**HSASox17** **EFEQYLHFV-** **CKPE----M-** **GLPYQGH---** **--DSGVNLPD** **SHGAISSVVS** **DASSAVYYCN** **YPDV------** **----------** **----------**

**HSASox18** **EFDQYLNCSR** **TRPD----AP** **GLPYHVALAK** **LGPRAMSCPE** **ESSLISA-LS** **DASSAVYYSA** **CISG------** **----------** **----------**

**CinSoxF**  **STNRYTDFT-** **----------** **----------** **----------** **AVTTCQSTGL** **EP--------** **----------** **----------** **----------**

**I**

**730**

**CheSox11** **----------**

**AmiSoxF**  **----------**

**NveSoxF1** **----------**

**DmeSoxF**  **PPCQCHPTRR**

**HSASox7**  **----------**

**HSASox17** **----------**

**HSASox18** **----------**

**CinSoxF**  **----------**
