# Supplementary material for: Multiple Sox genes are expressed in stem cells or in differentiating neuro-sensory cells in the hydrozoan Clytia hemisphaerica
Source: EvoDevo. 2011 Jun 1;2:12. doi: 10.1186/2041-9139-2-12 (PMC3120710; doi:10.1186/2041-9139-2-12)
Supplement: Additional file 4 — Alignment of group E Sox amino-acid sequences. Legend as for additional file 1. [file 2041-9139-2-12-S4.DOC]

Additional file 4

**10 20 30 40 50 60 70 80 90**

**CheSox1**  **----------** **----------** **----------** **----------** **----------** **----------** **----------** **----------** **----------**

**NveSoxE1** **----------** **----------** **----------** **----------** **----------** **----------** **----------** **----------** **----------**

**AmiSoxE1** **----------** **----------** **----------** **----------** **----------** **----------** **----------** **----------** **----------**

**HSASox8**  **----------** **----------** **----------** **----------** **----------** **----------** **----------** **----------** **----------**

**HSASox9**  **----------** **----------** **----------** **----------** **----------** **----------** **----------** **----------** **----------**

**HSASox10** **----------** **----------** **----------** **----------** **----------** **----------** **----------** **----------** **----------**

**CinSoxE**  **MVKTTGNTGV** **SSPHDRYVPQ** **VKAISSNCSL** **CTLQRESNNR** **RAPKYFMRQQ** **PRAVSIKCTC** **GASLTKAKTA** **SIPTKLDSVV** **MSVCFDAHQH**

**DmeSoxE**  **----------** **----------** **----------** **----------** **----------** **----------** **----------** **----------** **----------**

**PpiSox1**  **----------** **----------** **----------** **----------** **----------** **----------** **----------** **----------** **----------**

**PpiSox6**  **----------** **----------** **----------** **----------** **----------** **----------** **----------** **----------** **----------**

**HmaSox5**  **----------** **----------** **----------** **----------** **----------** **----------** **----------** **----------** **----------**

**CheSox5**  **----------** **----------** **----------** **----------** **----------** **----------** **----------** **----------** **----------**

**NveSoxE2** **----------** **----------** **----------** **----------** **----------** **----------** **----------** **----------** **----------**

**100 110 120 130 140 150 160 170 180**

**CheSox1**  **----------** **----------** **----------** **----------** **----------** **----------** **----------** **----------** **----------**

**NveSoxE1** **----------** **----------** **----------** **----------** **----------** **----------** **----------** **----------** **---------M**

**AmiSoxE1** **----------** **----------** **----------** **----------** **----------** **----------** **----------** **----------** **---------M**

**HSASox8**  **----------** **----------** **----------** **----------** **----------** **----------** **----------** **MLDMSEARSQ** **PPCSPSGTAS**

**HSASox9**  **----------** **----------** **----------** **----------** **--------MN** **LLDPFMKMTD** **EQEKGLSGAP** **SPTMSEDSAG** **SPCPSGSGSD**

**HSASox10** **----------** **----------** **----------** **----------** **----------** **--MAEEQDLS** **EVELSPVGSE** **EPRCLSPGSA** **PSLGPDGGGG**

**CinSoxE**  **HVATIMNNDM** **TLLSGHKQTM** **FNQQSGSQDK** **WSSVSPNSPD** **KDIMEHLRKG** **TDFSCFASSN** **DVEFSSGVEG** **KENGSKGKSG** **GPFSPEDLFK**

**DmeSoxE**  **----------** **----------** **----------** **----------** **----------** **----------** **----------** **----------** **MSDSSSSNCT**

**PpiSox1**  **----------** **----------** **----------** **----------** **----------** **----------** **----------** **-------TYL** **FSTHSTGAFS**

**PpiSox6**  **----------** **----------** **----------** **----------** **----------** **----------** **----------** **----------** **----------**

**HmaSox5**  **----------** **----------** **----------** **----------** **----------** **----------** **----------** **--------MM** **AASETNSNFA**

**CheSox5**  **----------** **----------** **----------** **----------** **----------** **----------** **----------** **---------M** **AATQPNLNVE**

**NveSoxE2** **----------** **----------** **----------** **----------** **----------** **-VEFATKMAA** **TPTTNDQAIA** **LNGPENPGEV** **KIEKVEQPKT**

**190 200 210 220 230 240 250 260 270**

**CheSox1**  **----------** **----------** **----------** **----------** **----------** **----------** **----------** **----------** **----------**

**NveSoxE1** **DKKVTEQQVQ** **AVL-------** **----------** **----------** **----------** **----------** **----------** **--GLDTDGSQ** **VRNHQLSNAI**

**AmiSoxE1** **TEEATNGEIT** **----------** **----------** **----------** **----------** **----------** **----------** **----NTDQRQ** **PKNMDLSSAI**

**HSASox8**  **SMSHVEDSDS** **DAP-------** **----------** **----------** **----------** **-----PSPAG** **SEGLGRAGVA** **VGGARGDPAE** **AADERFPACI**

**HSASox9**  **TENTRPQENT** **FPK-------** **----------** **----------** **----------** **----------** **----------** **--GEPDLKKE** **SEEDKFPVCI**

**HSASox10** **GSGLRASPGP** **GEL-------** **----------** **----------** **----------** **----------** **---------G** **KVKKEQQDGE** **ADDDKFPVCI**

**CinSoxE**  **PNQDENSTST** **SEIFSMSSPE** **SLSNFCNVKS** **AVAVARAAAT** **LLESKSSEEE** **NYDESLMNTG** **SSARSASPGT** **NDDLSDRDSN** **PEKDDMSKDI**

**DmeSoxE**  **KDRAKPVEAL** **VLA-------** **----------** **----------** **----------** **----------** **-------NYA** **LKAEQKEAQG** **QGGRKEDERI**

**PpiSox1**  **MHRHEPNSGA** **SDV-------** **----------** **----------** **----------** **----------** **----------** **----------** **----------**

**PpiSox6**  **----------** **----------** **----------** **----------** **----------** **----------** **----------** **----------** **----------**

**HmaSox5**  **THVIQNNNIT** **HAV-------** **----------** **----------** **----------** **----------** **----------** **----SQEQRK** **GEGYGSTTIS**

**CheSox5**  **THVHENESAS** **MVV-------** **----------** **----------** **----------** **----------** **----------** **--MQDTKPVI** **DVNHSSSSSI**

**NveSoxE2** **SDQNFNWKLS** **TTD-------** **----------** **----------** **----------** **----------** **----------** **--GVLVQSNA** **VHSTLGDPES**

**I**

**280 290 300 310 320 330 340 350 360**

**CheSox1**  **----------** **----------** **----------** **----------** **----------** **-----VWAQA** **ARRKLADQYP** **HLHNAELSKT** **LGRLWRMLSE**

**NveSoxE1** **ASAVNHVLD-** **-G-----YDW** **SLIPLPV-RV** **NGIK--TQ--** **---KPHVKRP** **MNAFMVWAQA** **VRRKLADQYP** **HLHNAELSKT** **LGKLWKLLND**

**AmiSoxE1** **ATAVNHVLD-** **-G-----YDW** **SLIPLPV-RV** **NGGH--KH--** **---KPHVKRP** **MNAFMVWAQA** **ARRKLADQYP** **HLHNAELSKT** **LGKLWKMLKD**

**HSASox8**  **RDAVSQVLK-** **-G-----YDW** **SLVPMPV-RG** **GGGGALKA--** **---KPHVKRP** **MNAFMVWAQA** **ARRKLADQYP** **HLHNAELSKT** **LGKLWRLLSE**

**HSASox9**  **REAVSQVLK-** **-G-----YDW** **TLVPMPV-RV** **NGSS--KN--** **---KPHVKRP** **MNAFMVWAQA** **ARRKLADQYP** **HLHNAELSKT** **LGKLWRLLNE**

**HSASox10** **REAVSQVLS-** **-G-----YDW** **TLVPMPV-RV** **NGAS--KS--** **---KPHVKRP** **MNAFMVWAQA** **ARRKLADQYP** **HLHNAELSKT** **LGKLWRLLNE**

**CinSoxE**  **KDAVSQVLK-** **-G-----YDW** **TLVPMPV-RM** **NGSQ--KT--** **---KPHVKRP** **MNAFMVWAQA** **ARRKLADQYP** **HLHNAELSKT** **LGKLWRLLSE**

**DmeSoxE**  **TTAVMKVLE-** **-G-----YDW** **NLVQASA-KA** **PSD---RK--** **---KEHIKRP** **MNAFMVWAQA** **ARRVMSKQYP** **HLQNSELSKS** **LGKLWKNLKD**

**PpiSox1**  **--NIMEIIK-** **-S-----FNW** **NITPDRV-DY** **TTLTD-DG--** **---TKRIKRP** **MNAFMVWAQA** **ARRKLAERHP** **YLHNAELSKT** **LGKVWKQLSE**

**PpiSox6**  **----------** **----------** **----------** **----------** **----SHVKRP** **MNAFMVWAQS** **ARRKLADQYP** **DLHNAELSKT** **LGKLWRMLSE**

**HmaSox5**  **LSEAAQCLKN** **LGEEGILCDD** **ALLRLTM-SS** **TSEHGETEFL** **DTKNSRVKRP** **MNSFMVWAQT** **ARKKLAEKYP** **HLHNAHLSKM** **LGKLWKMLSP**

**CheSox5**  **LSEAAEALR-** **-GDEGILSDE** **ALLRLTMTNT** **SGSPDVEPFL** **DTKNSRVKRP** **MNSFMVWAQT** **ARKKLAEKYP** **HLHNAHLSKM** **LGKLWKMLSP**

**NveSoxE2** **SNEVNSVVVS** **DCPQIDASLN** **VSGLPVKTQI** **MPGPYSEVQL** **PKKDPKVKRP** **MNSFMVWAQS** **ARRKLAEQYP** **HVHNAELSKM** **LGKLWRMLSA**

**HMG domain**

**370 380 390 400 410 420 430 440 450**

**CheSox1**  **EEKKPFMDEA** **ERLRLQHKKD** **HPDYKYQPRR** **KKQSKDGLPD** **NNEQEITASD** **LLRVIKGDKG** **ATLTKQ----** **----------** **----------**

**NveSoxE1** **SEKKPFIEEA** **ERLRIKHKRE** **HPDYKYQPRK** **KKQKGNGNGD** **AGDATISADD** **LLKVLKGDSK** **LVPNNGDA--** **----------** **----------**

**AmiSoxE1** **AEKKPFIEEA** **ERLRLKHKRE** **HPDYKYQPRR** **KKQKGNGGPD** **QPEATISADD** **LLKVLKGDPQ** **VVGKSMSQ--** **----------** **----------**

**HSASox8**  **SEKRPFVEEA** **ERLRVQHKKD** **HPDYKYQPRR** **RKSAKAGHSD** **SDSGAELGPH** **PGGGAVY---** **----------** **----------** **----------**

**HSASox9**  **SEKRPFVEEA** **ERLRVQHKKD** **HPDYKYQPRR** **RKSVKNGQAE** **AEEATEQTHI** **SPNAIFKALQ** **ADSP------** **----------** **----------**

**HSASox10** **SDKRPFIEEA** **ERLRMQHKKD** **HPDYKYQPRR** **RKNGKAAQGE** **AECPGGEAEQ** **GGTAAIQAHY** **KSAHLDHR--** **----------** **----------**

**CinSoxE**  **TEKKPFVDEA** **ERLRIKHKKD** **HPDYKYQPRR** **RKSSKTASGV** **GEGTQGAQNQ** **SLKQQSGKVR** **KQDSQSSDEC** **QGVQQALVAN** **PISGKQQSKQ**

**DmeSoxE**  **SDKKPFMEFA** **EKLRMTHKQE** **HPDYKYQPRR** **KKARVLPSQH** **SGDGGSPGPE** **MTLSATMGSS** **GKPRSSNSNG** **QRRAGKGNAA** **AD--------**

**PpiSox1**  **PDKRPFVEEA** **ERLRQQHRRE** **HPEYKYRPKR** **RKSSPIPPMR** **QGSEDQQQLA** **LKSEPSECSN** **QLNASQL---** **----------** **----------**

**PpiSox6**  **TDKHPYIKES** **ERLRMIHKKQ** **HPEYKYRPKK** **RKHLKRPTER** **LPHEIESLAK** **RALLNPNHEF** **TANTRALLSQ** **QET-------** **----------**

**HmaSox5**  **DEKQPYVLEA** **SRLDKLHKDE** **HPEYKYRPRR** **RPKGLKRGYS** **TPTMIVSPTT** **TSKPYTIPSN** **WARIVHAPE-** **----------** **----------**

**CheSox5**  **EEKQPYVEEA** **ARLDKRHKDE** **HPEYKYRPRR** **RPKGGKRGYN** **ATPTMMVSPS** **GAVGGKPYAV** **PTQWARVVQT** **AEG-------** **----------**

**NveSoxE2** **AEKQPYVDEA** **ARLDKRHKED** **HPDYKYRTRR** **RQKSLKRAYG** **QPPRVVPNWA** **IQHAQDHFKT** **QVI-------** **----------** **----------**

**460 470 480 490 500 510 520 530 540**

**CheSox1**  **-------RTE** **STSDYSEG-Q** **SPQMS-----** **------PYPE** **CSPKSSCSSS** **NPFSPNECGS** **N------KNS** **DNTGQTSVTE** **PIDLPNSSL-**

**NveSoxE1** **---------S** **ASCASPES-V** **SDGEVS----** **------SSES** **CSVPSPETPT** **AVPVKNEDVK** **N-----DEAL** **SAQPGFPSCS** **KKDDSNSHA-**

**AmiSoxE1** **---------D** **SSCASPDSSL** **SDEESS----** **------PGSS** **SPATSPPTTP** **TATIKVEGGS** **KCSEPSVPDP** **DIQAQSSPTF** **PLKKSENSTV**

**HSASox8**  **---------K** **AEAGLGDGHH** **HGDHT-----** **----------** **-GQTHGPPTP** **PTTPKTELQQ** **AGAK------** **PELKLEGRRP** **VDSGRQNIDF**

**HSASox9**  **---------H** **SSSGMSEVHS** **PGEHS-----** **----------** **-GQSQGPPTP** **PTTPKTDVQP** **GK--------** **ADLKREGRPL** **PEGGRQP-PI**

**HSASox10** **---------H** **PGEGSPMSDG** **NPEHPS----** **----------** **-GQSHGPPTP** **PTTPKTELQS** **GK--------** **ADPKRDGRSM** **GEGGKPHIDF**

**CinSoxE**  **QLSSHHSPQS** **VCHSPSNQSP** **PHQGSITNIY** **EVMHREQRGY** **HDSPDATVPC** **SPPTAINMDE** **SKKPHSTLHS** **RNSSFGSKQQ** **-RGSSIDLTS**

**DmeSoxE**  **--------LG** **SCAATISHAN** **VGSSS-----** **SDVFSNEAFM** **KSLNSACAAS** **LMEQSLIETG** **LDSPCSTASS** **MSSLTPPATP** **YNVAPSNAKA**

**PpiSox1**  **--------AE** **QASLPSFYQY** **PTQMS-----** **-------PFM** **EYGNPSNLYC** **EQQQTSTEQL** **IQHPFSPVTI** **QQHVQESIQQ** **---PIPTLPP**

**PpiSox6**  **---------Q** **NACVSPDSST** **MPGSSS----** **----------** **-DQRSSCQFR** **YDGPISSFPS** **NI--------** **-DTSTADRNR** **LFGGD-Q-TI**

**HmaSox5**  **---------N** **ENLISPRAEA** **AQIV------** **----------** **-YATDGMQYY** **AVQKGSGFTP** **TMAH------** **HHGNSVIVSP** **TNQPITLVQS**

**CheSox5**  **---------E** **TTITSPRPEA** **VTPQII----** **----------** **-YGADGTQYY** **AV-PKASFAP** **SMVA------** **PSATAAGGSV** **IVSPTQVAAP**

**NveSoxE2** **----------** **----------** **----------** **----------** **----------** **----------** **----------** **----------** **----------**

**II**

**550 560 570 580 590 600 610 620 630**

**CheSox1**  **-------ESM** **IKSNIQCIGS** **KPAINELDIN** **SLDQYLPTTE** **GFNFEQK---** **----------** **----------** **----------** **----------**

**NveSoxE1** **---------I** **DFDVGDLTTD** **LMAMGDVDST** **EFDQYLPTYS** **QALLDST---** **----------** **----------** **----------** **----------**

**AmiSoxE1** **PSTTN----N** **SGNSAIDFHV** **EMGDLMVDTT** **EFDQYLHSYT** **QPLQMPS---** **----------** **----------** **----------** **----------**

**HSASox8**  **SNVD------** **--ISELSSEV** **MGTMDAFDVH** **EFDQYLPLGG** **PAPPEPG---** **----QAYG--** **----------** **----------** **-------GAY**

**HSASox9**  **DFRDVD----** **--IGELSSDV** **ISNIETFDVN** **EFDQYLPPNG** **HPGVPATHGQ** **VTYTGSYG--** **----------** **-----ISSTA** **ATPASAGHVW**

**HSASox10** **GNVD------** **--IGEISHEV** **MSNMETFDVA** **ELDQYLPPNG** **HPGHVSS---** **-YSAAGYG--** **----------** **----------** **-----LGSAL**

**CinSoxE**  **ECSSHSMHG-** **--VMDTNTQD** **IMAKPGFDVT** **EFEQYMPGAC** **NPAVVRQHEE** **AFGYSCMGEP** **HAKQKRCNFT** **DTSQNMPSPV** **DCSVQQNPMF**

**DmeSoxE**  **SAANNPSLLL** **RQLSEPVANA** **GDGYGVLLEA** **GREYVAIGEV** **NYQGQSAGVQ** **SGAEGGG---** **----------** **--AGQEMDFL** **ENINGYGGYT**

**PpiSox1**  **NCSVDHLVSA** **FSSSYKADLG** **MTGHHHLQ-H** **PAEDIQQQST** **RAMRPLTHHD** **SRRSEPYP--** **----------** **----TNRTSV** **LSPVARSIRM**

**PpiSox6**  **YTTP----PS** **IL----AGYI** **RPSDLFRSEH** **MCL-----FP** **KRPYLNMPTT** **STA------T** **ERFH------** **----------** **----QPTVKV**

**HmaSox5**  **TSAP------** **----------** **----------** **-------TSN** **YPAYVTVPMP** **ATS------T** **AIFH------** **----------** **----PIAIPV**

**CheSox5**  **QQIP------** **------HQVA** **AAAPTASAGT** **ATATTTSSTN** **YPAFVTVPMP** **ATS------T** **AIFH------** **----------** **----PVAIPV**

**NveSoxE2** **----------** **----------** **----------** **----------** **----------** **----------** **----------** **----------** **----------**

**III**

**640 650 660 670 680 690 700 710 720**

**CheSox1**  **----TTQSSA** **TKTQAVYNKM** **SSYQY-----** **-----SPSIS** **RTEYSP----** **----------** **----------** **-------TYY** **QMNTIDSQ--**

**NveSoxE1** **-----LTKAI** **NTTQINTQSL** **SNSRF-----** **-----TTSQA** **VQSPPP----** **----------** **----------** **-------LPS** **SYREFMVQ--**

**AmiSoxE1** **-----SPPSV** **TYTSHGGVYT** **PTTHG-----** **------VSRF** **APNNNP----** **----------** **----------** **-------VTS** **SYTEFMEQ--**

**HSASox8**  **FHAGASPVWA** **HKSAPSASAS** **PTETG-----** **-----PPRPH** **IKTEQP----** **----------** **----------** **-------SPG** **HYGDQPRG--**

**HSASox9**  **MSKQQAPPPP** **PQQPPQAPPA** **PQAPP-----** **-----QPQAA** **PPQQPAAPPQ** **QPQAHTLTTL** **SSEPGQSQRT** **HIKTEQLSPS** **HYSEQQQH--**

**HSASox10** **AVASGHSAWI** **SKPPGVALPT** **VSPPG---VD** **AKAQVKTETA** **GPQGPP----** **----------** **----------** **----------** **HYTDQPS---**

**CinSoxE**  **SPNKQGTPQY** **RPSSWVEGYE** **TGVMTEASVS** **PNASMDQQFS** **QPENFSYDSV** **TSACSPLQSS** **SVTSQSGEFN** **TYSMNQSSPH** **SCAELPVK--**

**DmeSoxE**  **GSRVSYPAYS** **YPANGGHFAT** **EEQQ-----Q** **QQALQASEAL** **NYKPA-----** **---------A** **ADIDPKEIDQ** **YFMDQMLPMT** **QHHHPHH---**

**PpiSox1**  **ANEVTGLTPV** **EFINCYTGFY** **PSETG-----** **-----ILQEP** **LQMGQP----** **----------** **----------** **--------TT** **LLSDLFVD--**

**PpiSox6**  **EHKISFFSS-** **-QLPTVNPLP** **AFPGYKSPPQ** **S---------** **-VSPTTTPSI** **YP--------** **----------** **----------** **---GPGAYKT**

**HmaSox5**  **QGV-------** **-HTPAFVLRS** **SYPGSNILSP** **S---------** **----------** **----------** **----------** **----------** **--AIPIAYHN**

**CheSox5**  **QGV-------** **-HTPAYVLRP** **PYPGSTMYQP** **T---MVQSVA** **TTTTGSAATG** **VP--------** **----------** **----------** **AGTIPIAYHN**

**NveSoxE2** **----------** **----------** **----------** **----------** **----------** **----------** **----------** **----------** **----------**

**730 740 750 760 770 780 790 800 810**

**CheSox1**  **---VSASST-** **RHYRASNRYN** **P-YQYDSTKS** **YTDMMGVDCS** **NQNLNFNQYP** **STSPTINTNI** **PTTCKQDNFV** **LSGNTSTCLY** **SNVSNHQQHY**

**NveSoxE1** **---LQKLP--** **SEGSFPPNRV** **APSATMQQRN** **QPDSNSF-FP** **FSESEVNVCS** **S------LAA** **TRQPAFLSSP** **STSGTLSSSS** **NSGRTHTLIW**

**AmiSoxE1** **---LQKLP--** **--SSYPPNQV** **APSA-LHQRN** **QSENGSFPFS** **FNEPADVANG** **SSPRSFGISS** **VPSLAIPASS** **PANGTQNPQS** **SPSRLHTLMW**

**HSASox8**  **---SPDYGS-** **CSGQSSATPA** **APAGPFAGSQ** **GDYGDLQASS** **YYGAYPGYAP** **GLYQYPCFHS** **--PRRPYASP** **LLNG---LAL** **PPAHSPTSHW**

**HSASox9**  **---SPQQIA-** **YSPFNLPHYS** **PSYPPITRSQ** **YDYTDHQNSS** **SYYSHAAGQG** **TGLYSTFTYM** **NPAQRPMYTP** **IADTSGVPSI** **PQTHSPQ-HW**

**HSASox10** **---TSQIA--** **YTSLSLPHYG** **SAFPSISRPQ** **FDYSDHQPSG** **PYYGH-SGQA** **SGLYSAFSYM** **GPSQRPLYTA** **ISD--PSPSG** **PQSHSPT-HW**

**CinSoxE**  **--KEQISP-I** **QQHFFPHVPP** **ATRFQCSELH** **KAADVAPQPY** **QAYFDANIQR** **APMEDTMVDQ** **RADALYHHNA** **YDHSGPFPNM** **HGRFDACSAK**

**DmeSoxE**  **--THPLHH-P** **LHHSPPLNSS** **AS--LSSACS** **SASSQQPVAE** **YYEHLGYSPA** **ASSASQNPNF** **GPQQPYANGA** **ASMTPTLGDP** **APQQELQSQQ**

**PpiSox1**  **---SNELDR-** **YLPGDPAGET** **TPLSHLPLL-** **----------** **-------GSP** **AQLWTNQLSV** **NQTNRPPCNI** **KPTQFVSPED** **QTDSIVRRV**

**PpiSox6**  **SPXT-----V** **SNHS--AAMQ** **IPSLQNPPLG** **ARRAVITQLR** **VA-LLPVRVK** **S-TPCLLMTS** **KITLKSLICI** **CPRARSEGQQ** **EIELGISLLA**

**HmaSox5**  **IDHRTIESHM** **QPNQHIKTES** **TNTIVKTPDG** **ETIVIIQRPI** **DGHVVPFQLR** **E---------** **----------** **-VPSGATNTI** **TVVPQSQ---**

**CheSox5**  **IADTRIDAAT** **HAAIS-KAEG** **TNTIVKTPDG** **ETIVIIQRPM** **EG-AVPMQLN** **KDGTVAAHPQ** **YYQQHVVHTG** **QPHQEIKHQI** **IIDPSQTPTA**

**NveSoxE2** **----------** **----------** **----------** **----------** **----------** **----------** **----------** **----------** **----------**

**820 830 840 850 860 870 880 890 900**

**CheSox1**  **N---------** **----------** **----------** **----------** **----------** **----------** **----------** **----------** **----------**

**NveSoxE1** **K---------** **----------** **----------** **----------** **----------** **----------** **----------** **----------** **----------**

**AmiSoxE1** **K---------** **----------** **----------** **----------** **----------** **----------** **----------** **----------** **----------**

**HSASox8**  **DQPVYTTLTR** **P---------** **----------** **----------** **----------** **----------** **----------** **----------** **----------**

**HSASox9**  **EQPVYTQLTR** **P---------** **----------** **----------** **----------** **----------** **----------** **----------** **----------**

**HSASox10** **EQPVYTTLSR** **P---------** **----------** **----------** **----------** **----------** **----------** **----------** **----------**

**CinSoxE**  **QQEMNLDSPT** **RCSPDPPRPF** **EHAAYDSNIT** **NPLQRRFSLP** **LIPQNQQQGG** **ANPAYRHFGH** **SHNQSLASHY** **PKPNERQQLY** **SAPEGFSYPH**

**DmeSoxE**  **QEQQHQNPSQ** **HHLWGTYTYV** **NP--------** **----------** **----------** **----------** **----**

**PpiSox1**  **----------** **----------** **----------** **----------** **----------** **----------** **----------** **----------** **----------**

**PpiSox6**  **KCIMKYH---** **----------** **----------** **----------** **----------** **----------** **----------** **----------** **----------**

**HmaSox5**  **----------** **----------** **----------** **----------** **----------** **----------** **----------** **----------** **----------**

**CheSox5**  **TMY-KFKRLF** **HYITSFQGYD** **YPFLLCHAPF** **TIVLSLIFSF** **IKFYCLPLYQ** **PLNLKCTFCS** **NQIHYNNSVQ** **SHIMINYIRK** **KNTRFYKLYI**

**NveSoxE2** **----------** **----------** **----------** **----------** **----------** **----------** **----------** **----------** **----------**

**910 920 930**

**CheSox1**  **----------** **----------** **----------** **-**

**NveSoxE1** **----------** **----------** **----------** **-**

**AmiSoxE1** **----------** **----------** **----------** **-**

**HSASox8**  **----------** **----------** **----------** **-**

**HSASox9**  **----------** **----------** **----------** **-**

**HSASox10** **----------** **----------** **----------** **-**

**CinSoxE**  **NQHYNMAQQQ** **QNWPLPSTSA** **EVFSPPH---** **-**

**DmeSoxE**  **----------** **----------** **----------** **-**

**PpiSox1**  **----------** **----------** **----------** **-**

**PpiSox6**  **----------** **----------** **----------** **-**

**HmaSox5**  **----------** **----------** **----------** **-**

**CheSox5**  **FIEKLSTLGN** **KKN-------** **----------** **-**

**NveSoxE2** **----------** **----------** **----------** **-**
