# Supplementary figures and images for: Multiple Sox genes are expressed in stem cells or in differentiating neuro-sensory cells in the hydrozoan Clytia hemisphaerica
Source: EvoDevo. 2011 Jun 1;2:12. doi: 10.1186/2041-9139-2-12 (PMC3120710; doi:10.1186/2041-9139-2-12)

Additional file 5


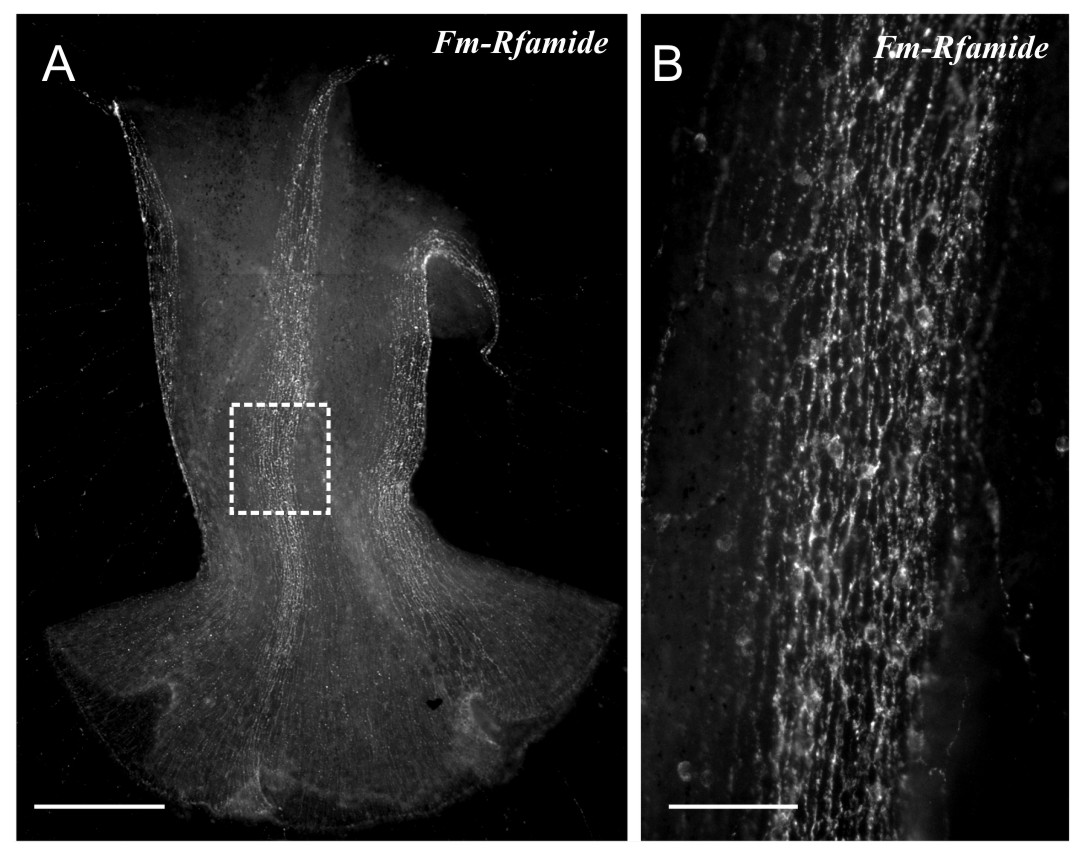

Supplement: Additional file 5 — Dense nerve net on the manubrium ridges revealed by FMRFamide immuno-staining. (A) FMRFamide immunofluorescence staining of a manubrium showing marked/strong condensation of the nerve net along the four longitudinal ridges. (B) Higher magnification view of the region indicated by the box in A, showing the aspect of the nerve net. Scale bars: A, 50 μm; B-C, 20 μm. [file 2041-9139-2-12-S5.DOC]

Additional file 6


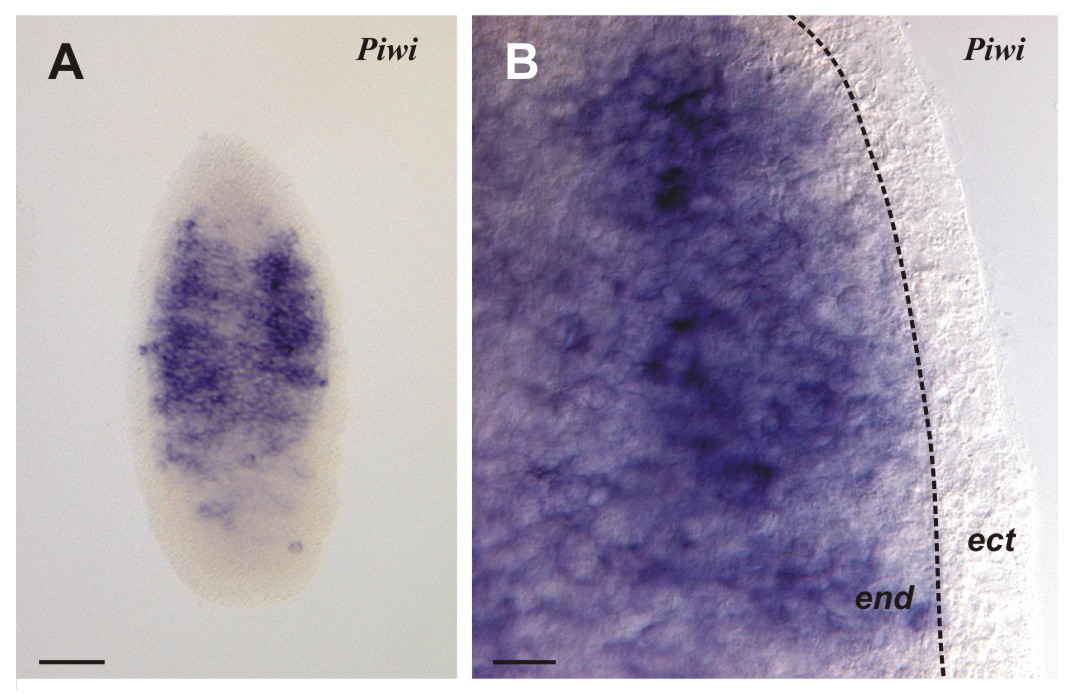

Supplement: Additional file 6 — Piwi expression in the planula larva. (A) Expression pattern of Piwi in a two-day-old planula (oral pole on the top). (B) Higher magnification view showing the distribution and aspect of the interstitial stem cells. ect: ectoderm; end: endoderm. Scale bars: A, 50 μm; B, 10 μm. [file 2041-9139-2-12-S6.DOC]

Additional file 7


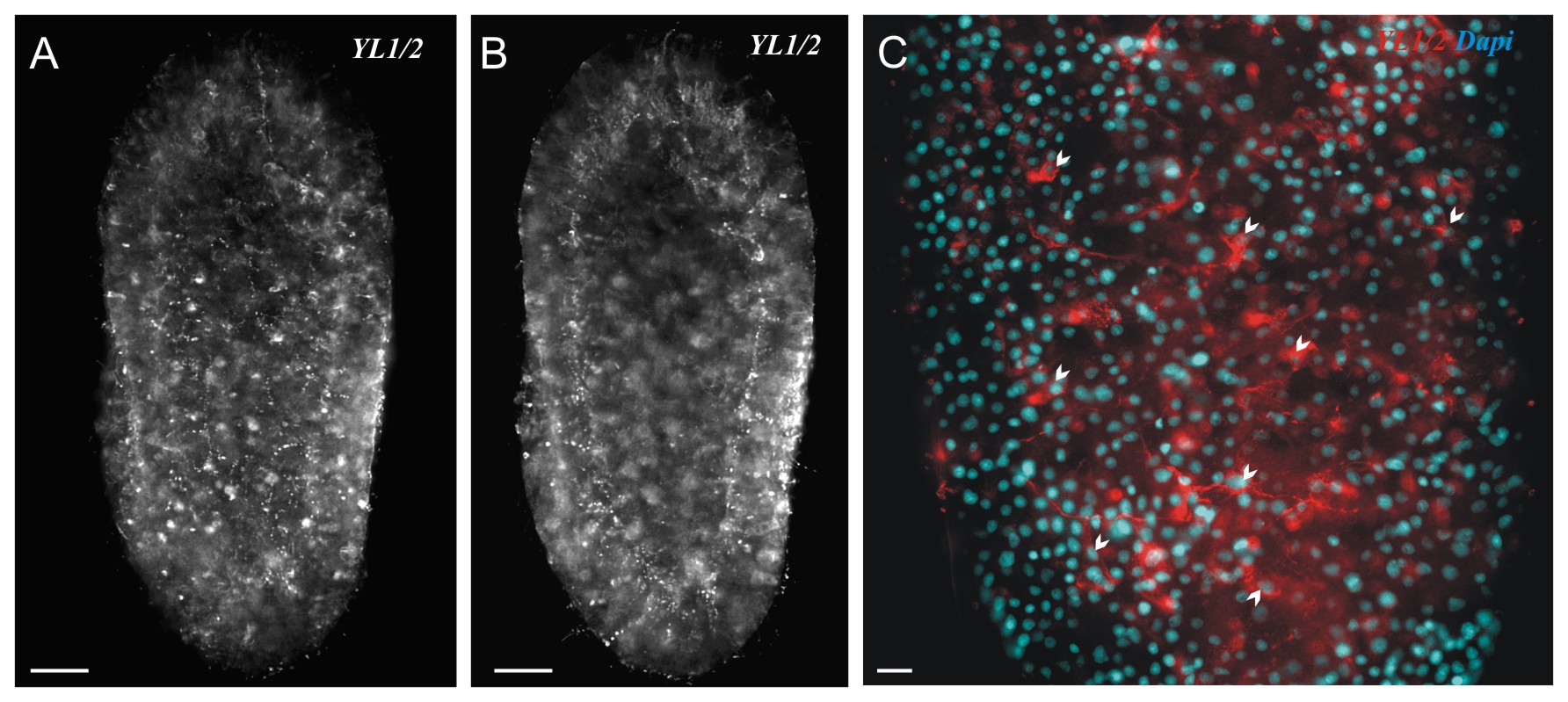

Supplement: Additional file 7 — Distribution of the nerve net in two-day-old planula detected using YL1/2 antibody. (A) Superficial view (optical plane on the basal part of the ectodermal epithelium). (B) Deeper view of the same specimen (optical plane crossing the larval endoderm and cavity). (C) Higher magnification view of the YL1/2 staining (in red) with Dapi counter-staining (in blue) showing the distribution and aspect of nerve cell bodies (white arrowheads) and neurites. Oral pole is on the top for all pictures. Scale bars: A-B, 50 μm; C, 10 μm. [file 2041-9139-2-12-S7.DOC]

Additional file 8


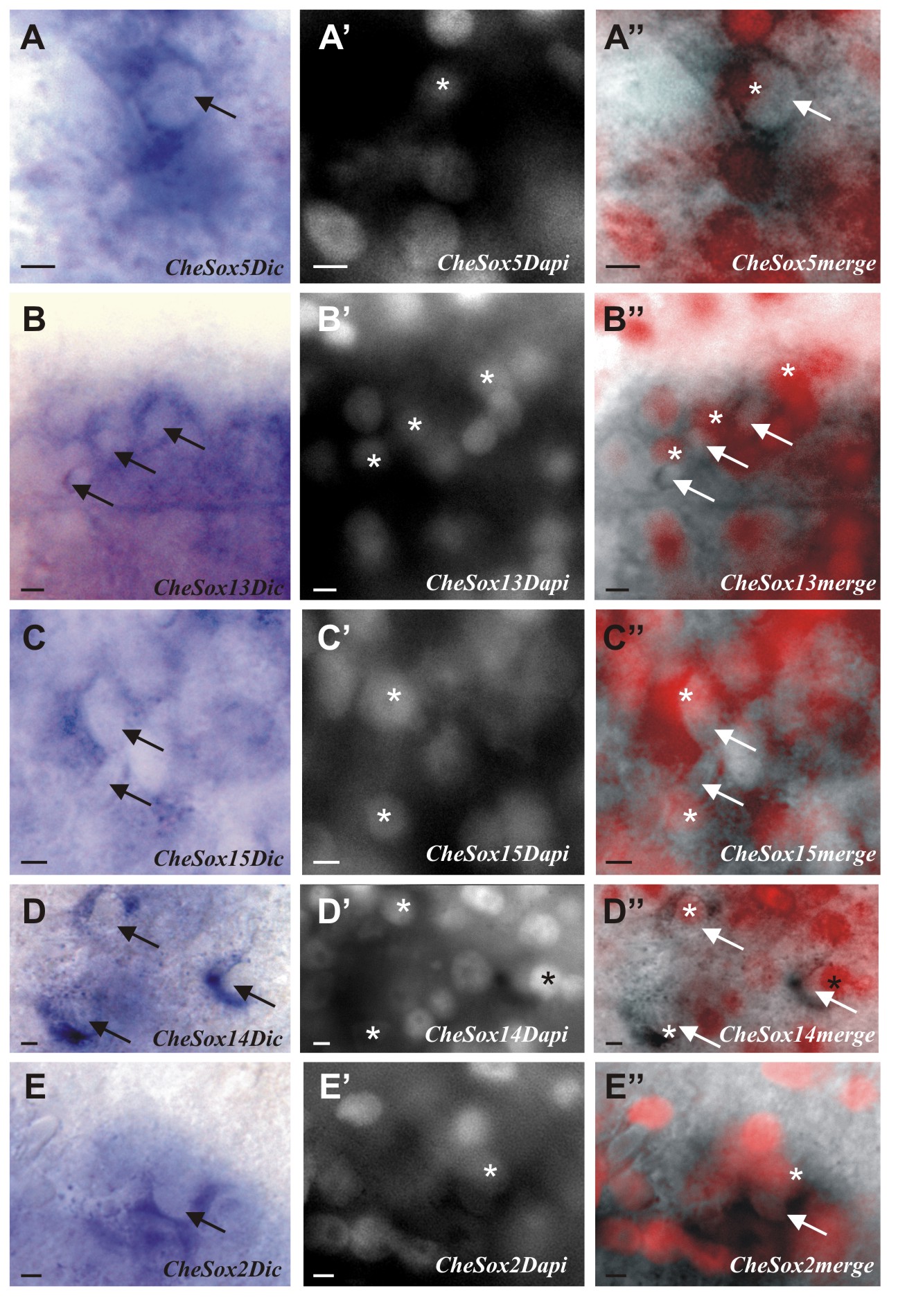

Supplement: Additional file 8 — Detailed views of the capsules and DAPI-stained nuclei in CheSox5, CheSox13, CheSox15, CheSox14 and CheSox2 expressing nematoblasts. (A-E) Transcript distribution after ISH, viewed at high magnification, for the five Sox genes cited above. The signal is concentrated around unstained maturating capsules (black arrows). (A'-E') Dapi counter-staining of (A-E). The white stars indicate DAPI-stained nuclei of the cells containing ISH signal in (A-E). (A"-E") Merged pictures combining the ISH signal (in black and white, pictures A-E) and the DAPI signal (in red, pictures A'-E'). Maturating nematoblast capsules are indicated by white arrows and nuclei of the corresponding cells by white stars. Scale bars: A-E": 2 μm. [file 2041-9139-2-12-S8.DOC]
