# Supplementary material for: Multiple Sox genes are expressed in stem cells or in differentiating neuro-sensory cells in the hydrozoan Clytia hemisphaerica
Source: EvoDevo. 2011 Jun 1;2:12. doi: 10.1186/2041-9139-2-12 (PMC3120710; doi:10.1186/2041-9139-2-12)
Supplement: Additional file 9 — Alignment of Sox HMG domain sequences used for the phylogenetic analyses (in Fasta format). [file 2041-9139-2-12-S9.DOC]

Additional file 9:

>DmeSoxE

EHIKRPMNAFMVWAQAARRVMSKQYPHLQNSELSKSLGKLWKNLKDSDKKPFMEFAEKLRMTHKQEHPDYKYQP-RRKKA

>AmiSoxE1

PHVKRPMNAFMVWAQAARRKLADQYPHLHNAELSKTLGKLWKMLKDAEKKPFIEEAERLRLKHKREHPDYKYQP-RRKKQ

>NveSoxE1

PHVKRPMNAFMVWAQAVRRKLADQYPHLHNAELSKTLGKLWKLLNDSEKKPFIEEAERLRIKHKREHPDYKYQP-RKKKQ

>CheSox1

-----------VWAQAARRKLADQYPHLHNAELSKTLGRLWRMLSEEEKKPFMDEAERLRLQHKKDHPDYKYQP-RRKKQ

>HSASox8

PHVKRPMNAFMVWAQAARRKLADQYPHLHNAELSKTLGKLWRLLSESEKRPFVEEAERLRVQHKKDHPDYKYQP-RRRKS

>HSASox9

PHVKRPMNAFMVWAQAARRKLADQYPHLHNAELSKTLGKLWRLLNESEKRPFVEEAERLRVQHKKDHPDYKYQP-RRRKS

>HSASox10

PHVKRPMNAFMVWAQAARRKLADQYPHLHNAELSKTLGKLWRLLNESDKRPFIEEAERLRMQHKKDHPDYKYQP-RRRKN

>CinSoxE

PHVKRPMNAFMVWAQAARRKLADQYPHLHNAELSKTLGKLWRLLSETEKKPFVDEAERLRIKHKKDHPDYKYQP-RRRKS

>PpiSox1

KRIKRPMNAFMVWAQAARRKLAERHPYLHNAELSKTLGKVWKQLSEPDKRPFVEEAERLRQQHRREHPEYKYRP-KRRKS

>PpiSox6

SHVKRPMNAFMVWAQSARRKLADQYPDLHNAELSKTLGKLWRMLSETDKHPYIKESERLRMIHKKQHPEYKYRP-KKRKH

>CheSox5

SRVKRPMNSFMVWAQTARKKLAEKYPHLHNAHLSKMLGKLWKMLSPEEKQPYVEEAARLDKRHKDEHPEYKYRP-RRRPK

>HmaSox5

SRVKRPMNSFMVWAQTARKKLAEKYPHLHNAHLSKMLGKLWKMLSPDEKQPYVLEASRLDKLHKDEHPEYKYRP-RRRPK

>NveSoxE2

PKVKRPMNSFMVWAQSARRKLAEQYPHVHNAELSKMLGKLWRMLSAAEKQPYVDEAARLDKRHKEDHPDYKYRT-RRRQK

>PpiSox4

YHVKRPMNAFMVWSQIERKKLADQHPDLHNSELSKMLGHLWRMLSEDEKRPFVEKAEKLRAQHMEEHPGYKYRP-RRRQQ

>EmuSox1

-----------VWSSIERKKLAEREPRLHNTELSKRLGQMWKCMTEEDKKPFRLEAEKLKTKLLEEHPDYKYRP-RRKKF

>AquSoxF

GRIKRPMNAFMVWSSLERKKLAEKEPNLHNTELSKRLGQMWKEMTEEDKTPYRQEATRLKDKLMEDHPEYKYKP-RRRKD

>HSASox7

SRIRRPMNAFMVWAKDERKRLAVQNPDLHNAELSKMLGKSWKALTLSQKRPYVDEAERLRLQHMQDYPNYKYRP-RRKKQ

>HSASox17

SRIRRPMNAFMVWAKDERKRLAQQNPDLHNAELSKMLGKSWKALTLAEKRPFVEEAERLRVQHMQDHPNYKYRP-RRRKQ

>HSASox18

SRIRRPMNAFMVWAKDERKRLAQQNPDLHNAVLSKMLGKAWKELNAAEKRPFVEEAERLRVQHLRDHPNYKYRP-RRKKQ

>DmeSoxF

SRIRRPMNAFMVWAKIERKKLADENPDLHNADLSKMLGKKWRSLTPQDRRPYVEEAERLRVIHMTEHPNYKYRP-RRRKQ

>CinSoxF

PRIRRPMNAFMCWAKTERKRMAAAFPDHHNAELSKMLGKKWKEMSNEDKRPYITEAEKLRMKHMQEHPDYKYRP-RRKPK

>AmiSoxF

DRIKRPMNAFMVWAQVERRRLADANPELHNAELSKILGQAWRALNGLQKRPFVEEAERLRQQHIKDHPDYKYRP-RRRKH

>NveSoxF1

ERIKRPMNAFMVWAQVERRRLADANPELHNAELSKMLGLTWRALNSTQKRPFVDEAERLRLQHMQDYPNYKYRP-RRRKH

>PpiSox8

IHIKRPMNAFMCWAQIARRRLADERPELRNTDLSKILGRMWKELGDEQKRPYIKKAEKLRLQHKSQYPNYKYRP-KRKRH

>CheSox11

DRIRRPLNCFMVFSHLERKRVAEEHPELHNADLSKILGKRWKTLSPSEKQPYIEEAERIRQLHTEIYPDYKYQP-RRKNQ

>CheSox12

DHVKRPMNAFMVWSQIERKKMAESYPDMHNAEISRRLGKQWKMLTDDDRRPYVIRSEKLREEHMRRHPDYKYRP-KKKAK

>HmaSox4

DHVKRPMNAFMVWSQIERKKMADIYPDMHNAEISRRLGKRWKLLSDADRRPFVIRSEKLREEHMRRYPDYKYRP-KKKAK

>AquSoxXC

EHIKRPMNAFMVWAQLERRKMTTEFPDMHNAEISRRLGKLWRLLSDREKQPYIEESERLRIQHMKQYPDYKYRP-RKKGG

>EmuSox3

-----------VWAQLERRKMTLEYPDMHNAEISRRLGKLWRLLTDAEKQPYVDESERLRVMHMKQYPDYKYRP-RKRDQ

>AmiSoxC

QHVKRPMNAFMVWSQIERRKMAEEHPDMHNAEISKRLGKRWKLLSESEKRPFVEESERLRIRHMQAYPDYKYRP-RKKKQ

>NveSoxC

QHVKRPMNAFMVWSQIERRKMAEEHPDMHNAEISKRLGKRWKLLSESEKRPFVEESERLRIRHMQAYPDYKYRP-RKKKQ

>PpiSox2

-----------VWSQIQRAKIVKEQPNKHNAAISKQLGSEWKMLSDEARMPYIHESQRLKRIHKQQYPDYKYRP-RKRGK

>PpiSox12

-----------VWSQIQRAKIVEEQPNKHNAAISKQLGSEWKMLSDEARMPYIHESQRLKRIHKQQYPDYKYRP-RKRGK

>HSASox4

GHIKRPMNAFMVWSQIERRKIMEQSPDMHNAEISKRLGKRWKLLKDSDKIPFIREAERLRLKHMADYPDYKYRP-RKKVK

>HSASox11

GHIKRPMNAFMVWSKIERRKIMEQSPDMHNAEISKRLGKRWKMLKDSEKIPFIREAERLRLKHMADYPDYKYRP-RKKPK

>HSASox12

GHIKRPMNAFMVWSQHERRKIMDQWPDMHNAEISKRLGRRWQLLQDSEKIPFVREAERLRLKHMADYPDYKYRP-RKKSK

>CinSoxC

GYIKRPMNAFMIWSQIERRKIMEKTPELHNAEISRNLGRIWREQADSIKRPFLIEAERLRLQHMCDYPDYKYKP-KKKAK

>DmeSoxC

GHIKRPMNAFMVWSQMERRKICERTPDLHNAEISKELGRRWQLLSKDDKQPYIIEAEKLRKLHMIEYPNYKYRP-QKKQT

>CelSoxC

NHIKRPMNAFMVWSQMERRKICEHQPDMHNAEISKQLGSRWRSLTDEEKAPFVAEAERLRVCHMQEYPDYKYKP-RKKPK

>HSASox5

PHIKRPMNAFMVWAKDERRKILQAFPDMHNSNISKILGSRWKAMTNLEKQPYYEEQARLSKQHLEKYPDYKYKP-RPKRT

>HSASox6

PHIKRPMNAFMVWAKDERRKILQAFPDMHNSNISKILGSRWKSMSNQEKQPYYEEQARLSKIHLEKYPNYKYKP-RPKRT

>HSASox13

SHIKRPMNAFMVWAKDERRKILQAFPDMHNSSISKILGSRWKSMTNQEKQPYYEEQARLSRQHLEKYPDYKYKP-RPKRT

>CinSoxD

NHIKRPMNAFMVWAKDERRRILQAFPDMHNSNISKILGAKWKEMSSNEKRPFYDEQTRLNKLHLEKYPDYKYKP-RPKRT

>CelSoxD

NHIKRPMNAFMVWARDERRKILKAYPDMHNSNISKILGSRWKGMSNSEKQPYYEEQSRLSKLHMEQHPDYRYRP-RPKRT

>DmeSoxD

PHIKRPMNAFMVWAKDERRKILKACPDMHNSNISKILGARWKAMSNADKQPYYEEQSRLSKLHMEQHPDYRYRP-RPKRT

>NveSoxJ

QHIKRPMNAFMVWSRTERRKLALKYPNMLNCEISKLLGAEWSRLSEEEKRPFVTEAKRLRTIHNQKYPDYSYKPRRRKSK

>HmaSox2

DHVKRPMNAFMVWSRTERRKLALKYPNMLNCEISKLLGAEWSRMGEEEKSPYIQESKRLRTIHSQKYPDYSYKPRRRKRK

>CinSoxB1

QRVKRPMNAFMVWSRGQRRKMAQDNPKMHNSEISKRLGAEWKTLSDSDKRPFIDEAKRLRALHMKEHPDYKYRP-RRKTK

>DmeSoxN

DRVKRPMNAFMVWSRGQRRKMASDNPKMHNSEISKRLGAQWKDLSESEKRPFIDEAKRLRAVHMKEHPDYKYRP-RRKTK

>HSASox1

DRVKRPMNAFMVWSRGQRRKMAQENPKMHNSEISKRLGAEWKVMSEAEKRPFIDEAKRLRALHMKEHPDYKYRP-RRKTK

>CelSoxB1

DRVKRPMNAFMVWSRGQRKKMALENPKMHNSEISKRLGTEWKMLSEQEKRPFIDEAKRLRAIHMKEHPDYKYRP-RRKTK

>HSASox3

DRVKRPMNAFMVWSRGQRRKMALENPKMHNSEISKRLGADWKLLTDAEKRPFIDEAKRLRAVHMKEYPDYKYRP-RRKTK

>HSASry

DRVKRPMNAFIVWSRDQRRKMALENPRMRNSEISKQLGYQWKMLTEAEKWPFFQEAQKLQAMHREKYPNYKYRP-RRKAK

>PpiSox3

-----------VWSRGQRRKMAQENPKMHNSEISKRLGLDWKMLTEDEKKPFIEEAKRLRALHMKEFPDYKYRP-RRKAK

>HSASox15

EKVKRPMNAFMVWSSAQRRQMAQQNPKMHNSEISKRLGAQWKLLDEDEKRPFVEEAKRLRARHLRDYPDYKYRP-RRKAK

>HSASox2

DRVKRPMNAFMVWSRGQRRKMAQENPKMHNSEISKRLGAEWKLLSETEKRPFIDEAKRLRALHMKEHPDYKYRP-RRKTK

>HSASox14

DHIKRPMNAFMVWSRGQRRKMAQENPKMHNSEISKRLGAEWKLLSEAEKRPYIDEAKRLRAQHMKEHPDYKYRP-RRKPK

>DmeSox21b

EHIKRPMNAFMVWSRLQRRKIAQDNPKMHNSEISKRLGAEWKLLTEEEKRPFIDEAKRLRAMHMKEHPDYKYRP-RRKPK

>DmeSox21a

DHIKRPMNAFMVWSRGQRRKMAQDNPKMHNSEISKRLGAEWKLLTEGQKRPFIDEAKRLRALHMKEHPDYKYRP-RRKPK

>DmeSoxdich

GHIKRPMNAFMVWSRLQRRQIAKDNPKMHNSEISKRLGAEWKLLAESEKRPFIDEAKRLRALHMKEHPDYKYRP-RRKPK

>HSASox21

DHVKRPMNAFMVWSRAQRRKMAQENPKMHNSEISKRLGAEWKLLTESEKRPFIDEAKRLRAMHMKEHPDYKYRP-RRKPK

>CinSoxB2

GHVKRPMNAFMVWSRGQRRKMAQENPKMHNSEISKRLGASWKLLNECEKRPFIDEAKRLRALHMKEHPDYKYRP-RRKPK

>CelSoxB2

DHVKRPMNAFMVWSRGQRRKMAQDNPKMHNSEISKRLGAEWKQLSEQEKRPFIDEAKRLRALHMKEHPDYKYRP-RRKPK

>AmiSoxB1

DRVKRPMNAFMVWSRERRRRMAQENPKMHNSEISKRLGAEWKQLSDPEKRPYVDEAKRLRAVHMKDHPDYKYRP-RRKSK

>NveSoxB1

DRVKRPMNAFMVWSRERRRKMAQDNPKMHNSEISKRLGSEWKLLSEQEKRPYIDEARRLRAVHMKEHPDYKYRP-RRKSK

>AmiSoxBa

GHIKRPMNAFMVWSRGKRKQYAAINPRMHNSEISKRLGAEWKMLSQDEKEPFVAEAKRLQAIHIQEHPDYKYKPKRRKPK

>NveSoxB2

GHVKRPMNAFMVWSRGKRKHYASINPRMHNSEISKRLGAEWKMLTAEEKEPFIAEAKRLQALHIQEHPDYKYKPKRRKPK

>HmaSox10

SHVKRPMNAFMVWSRGKRRQMAQDNPRMHNSEISKRLGAEWKCLTQQEKQPFIDEAKRLRAVHIQEHPDYKYKPKRRKQK

>CheSox10

NHVKRPMNAFMVWSRGKRRQMAQEHPRMHNSEISKRLGAQWKVLTPEEKQPFIDEAKRLRAVHIQEHPDYKYKPKRRKPK

>CheSox3

DRIKRPMNSFMVWSREKRRKLAQENPKMHNSEISKRLGAEWKVLTEEEKAPFVYEAKRLRAEHMKTHPDYKYRP-RRKNK

>HmaSoxB1

DKVKRPMNSFMVWSREKRRRLAHENPKMHNSEISKRLGAEWKVLTEDEKAPFVFEAKRLRAEHMKSHPDYKYRP-RRKLD

>NveSox1

EHVKRPMNAFMVWSREERRKIAQENPKMHNSEISKRLGSEWKQLADDDKKPFVEEAKKLRAQHMKEHPDYKYRP-RRMPK

>CheSox2

NHIKRPMNSFMVWSRMERKRISEENPKLHNSEISKRLGASWKMLSEEERKPFAEEAKRLRQIHIQEHPEYKYRP-RRKPK

>HmaSox3

GHIKRPMNSFMVWSRMERKRISEANPKMHNSEISKQLGTSWKMLSEEDRAPYAEEAKRLRDLHMSEYPDYKYRP-KRKPK

>NveSox2

DHIKRPMNAYMVWSRKERRRIAEECPRMLNSEISKRLGLEWNSLTLDEKQPYVEEAKRLRELHKKDHPDYKYQP-KRKPK

>AquSoxB1

DKVKRPMNAFMVWSRKMRKKIADENPKMHNSEISKRLGTQWKALSEEDKRPFIDEAKRLREAHMKKHPNYKYKP-KRKKQ

>EmuSox2

-----------VWSRKMRKKIADENPKMHNSEISKRLGAQWKALSDEEKRPYIEEAKRLREAHMKKHPNYKYKP-KRKKQ

>AquSoxB2

DHIKRPMNAFMVWSKERRKELAQENPRMHNSELSKKLGAEWKALSDTNKHRYIEEAKKIREQHMAEFPHYRYRP-RRKPK

>CheSox13

DHVKRPMNAFMVWSREKRKKMSQINPRMHNSEISKILGAEWKRMTEQEKGPYIEEAKRLQTQHSIEYPNYKYKPRRRKPK

>HmaSox1

DHVKRPMNAFMVWSREKRRKMAQINPRMHNSEISKILGSEWKRMGESEKGPYVLEAKRLQTQHSIEYPNYKYKPRRRKAK

>CheSox14

PKIKRPMNPFMIFGCEKRRKLAQVHPRMHNSEISKILGAEWKRMSDYEKAPYIQEAKRLKEQHSIEYPNYKFKANRRKPR

>HmaSoxBb

TKIKRPMNPFMIFGCEKRRKLAQVHPRMHNSEISKILGAEWKRMSDYEKSPYIQEAKRLKEQHSIEYPNYKFKANRRKPR

>NveSox3

NHVKRPMNAFMVWSKERRRIKSQECPRMHNSEISKILGCEWKATKDELKQPYIEKAKELQAQHSRENPGYKYKPRRRKPK

>AmiSoxBb

DHIKRPMNAFMVWSKEKRRTMSQKNPKMHNSEISKILGAQWKKMPDEEKAKYIEEAKRLQQEHSQKHPDYKYKPRRRKQK

>CelSoxJ

WKIRKSNDYIKVWSQQRRQQIAATGQKFHNSDISKMLGAEWRKMEEHEKVPFVERAKQLREEHFNAHPDYVYRP-RRRKR

>NveSoxA

SHIKRPMNAFMIWSSKKRRQLAAENPKLHNSQISKMLGTEWRKLTVEEKQKFFAEAKLLNELHMIEHPDYKYRP-RRRVK

>NveSox4

THVKRPMNCFMVWSREKRCQILQENPGINNARLSKLLGMAWKKLSVEEKEPYIEKAKHLTEMHKEKHPDYKYQPKRRKSK

>HSASox30

GHVKRPMNAFMVWARIHRPALAKANPAANNAEISVQLGLEWNKLSEEQKKPYYDEAQKIKEKHREEFPGWVYQP-RPGKR

>NveSox5

DHVKRPLNSFMVWAKEKRRAMNRENPKMRNAEISKILGDEWRKMPESEKLPYTEEALRLRRQHKVDHPNYRYKP-RRKHK

>HmaSox6

KTAKRPMNSFLLWAKSVRKIYASENPNLSNTEISRLLGKIWKEMSEVEKLPFIQSAKCLRTKFIQDYPNYHYFL-KKKKF

>HmaSox7

KTAKRPMNSFLLWAKSVRKNFSNDNPNLTNTEISRVLGKVWKEMSEVEKLPFIQSAKCLRTKFLNDYPNYQYLC-KKRKF

>HmaSox8

KNKKRSISSFSIWAETARKVFSQENPNKSRKEISKHLGIVWKNMSHNEKIPYVEKAKSLQKDYFMHHPNNHFLI-LERLN

>HmaSox9

EKIKRPMSSFLLWAKVTRKNYLKKNPHMHNSEVSKLLGYTWNQMSAIEKLPFTTQAKNLRTVHMTNHPKYCYST-KKYKH

>HmaSox11

DKVKRPMSSFLLWARIKRKNYSKKNPKMLNSEISKLLGYTWNKMSAIEKLPFTTQAKNLRTAYMTNHPKYSTKK-CEHNK

>NveSoxF2

SHVKRPMNSFMIWAKVMRRKFAEENPKLHNAEISKLLGKAWNELTTKDKRPFVEKAERLRIRHMKEHPNYRYTP-KRRGR

>CheSox15

DKIKRPMNAFMLWSKLRRREISKNDPTIHNAQISKLLGEEWKVLSTEDKQPFLRESQKLMAKHKKEHPNYRYKP-RKNKQ

>HmaSox12

KSVKRPMNAFMLWAKDRRRFLMENNPTLHNADISKILGSEWRDMPDEKKNPFWKEAHILMVQHKFDHPEYHYKP-RRQRQ

>CinCic

DHIRRPMNAFMIFSKMHRGLVHQRHPNQDNRTVSKILGEWWYALPSTEKQKYHNLAFQVKEAHFKAHPDWKWCS-KERKR

>HSACic

DHIRRPMNAFMIFSKRHRALVHQRHPNQDNRTVSKILGEWWYALGPKEKQKYHDLAFQVKEAHFKAHPDWKWCN-KDRKK

>DmeCic

KKIRRPMNAFMIFSKKHRKMVHKKHPNQDNRTVSKILGEWWYALKPEQKAQYHELASSVKDAHFKLHPEWKWCS-KDRRK

>Hma8806112

DHVRRPMNAFMIFSKKHRKDVHKRNPNQDNRTVSKILGEMWYNSSQDEQLKYKRLADDVKNAHYKNNPDWKWTT-KDKLK

>HSATcf1

PTIKKPLNAFMLYMKEMRAKVIAECTLKESAAINQILGRRWHALSREEQAKYYELARKERQLHMQLYPGWSARD-NYGKK

>HSATcf3

PHVKKPLNAFMLYMKEMRAKVVAECTLKESAAINQILGRKWHNLSREEQAKYYELARKERQLHSQLYPTWSARD-NYGKK

>HmaTcf

PHVKKPLNAFMLYMKEQRPKIAAEFTLKESAAINQILGKRWHALEKTEQAKYYEMARKERAIHMQLYPGWSARD-NYAQI

>NveTcf

QHIKKPLNAFMLYMKDMRPKVVAECTLKESAAINQILGKKWHALDRSEQAKYYEMARKERALHLQLYPGWSARD-NYAQQ

>NcrMATA1

AKIPRPPNAYILYRKDHHREIREQNPGLHNNEISVIVGNMWRDEQPHIREKYFNMSNEIKTRLLLENPDYRYNP-RRSQD

>SceIXRI

QGPKRPSSAYFLFSMSIRNELLQQFPEAKVPELSKLASARWKELTDDQKKPFYEEFRTNWEKYRVVRDAYEKTL-PPKRP

>SceMTHMG

QGPKRPTSAYFLYLQDHRSQFVKENPTLRPAEISKIAGEKWQNLEADIKEKYISERKKLYSEYQKAKKEFDEKL-PPKKP

>Hma9449115

QKKKQTMTAYLFFCKRYRPKIVARNPKLTFTQMSKLLSHMWRNASEHEKNTFRLKLKHHRTKVNAIVEKNLIEK-MKIVK

>Mbr6032738

NKPKNAQSAYMFFSQKVRPQFSKDNPDKKMTDVSKLIGAAWREMSDAAKKPYEEMARRDKQRYQHQMATYVPPP-TRELG

>GdoHMG2

NAPKRPPSAFFLFCSEHRPKIKNDHPGLSIGDTAKKLGEMWSEQSAKDKQPYEQKAAKLKEKYEKDIAAYRAKS-KSDAG

>HSAHMG2

NAPKRPPSAFFLFCSEHRPKIKSEHPGLSIGDTAKKLGEMWSEQSAKDKQPYEQKAAKLKEKYEKDIAAYRAKG-KSEAG

>HSAHMG1

NAPKRPPSAFFLFCSEYRPKIKGEHPGLSIGDVAKKLGEMWNNTAADDKQPYEKKAAKLKEKYEKDIAAYRAKG-KPDAA

>OmyHMGT

NAPKRPSSAFFIFCADFRPQVKGETPGLSIGDVAKKLGEKWNNLTAEDKVPYEKKASRLKEKYEKDITAYRNKG-KVPVS

>DmeHMG

NAPKRSLSAFFWFCNDERNKVKALNPEFGVGDIAKELGRKWSDVDPEVKQKYESMAERDKARYEREMTEYKTSG-KIAMS

>Hma133214

NAPKRPMSAFMLYMNEVREKIKADNPGIAFTDIAKKGGEQWKTLTDKTKWENMAKEAKNKYTIDFAAYSKTIKD-GGAAI

>CteHMG1b

DKPKRPLSAYMLWLNSARESIKRENPDFKVTEVAKKGGELWRGLKD--KSEWEAKAATAKQNYIRALQEYERNG-GGGDD

>DmeHMGd

DKPKRPLSAYMLWLNSARESIKRENPGIKVTEVAKRGGELWRAMKD--KSEWEAKAAKAKDDYDRAVKEF
